# An Atom-Precise Approach to Damp First-Order Phase Transitions and Its Implications for Neuromorphic Signal Processing

**DOI:** 10.1021/jacs.6c02370

**Published:** 2026-05-13

**Authors:** George Agbeworvi, Nitin Kumar, John D. Ponis, Shruti Hariyani, Nicholas Jerla, Fatme Jardali, Jialu Li, Wasif Zaheer, Joseph V. Handy, Jaime R. Ayala, Cherno Jaye, Conan Weiland, Daniel A. Fischer, Patrick J. Shamberger, Jinghua Guo, R. Stanley Williams, G. Sambandamurthy, Sarbajit Banerjee

**Affiliations:** † Department of Chemistry, 14736 Texas A&M University, College Station, Texas 77843, United States; ‡ Department of Physics, 12292 University at Buffalo, The State University of New York, Buffalo, New York 14260-1500, United States; § Laboratory for Inorganic Chemistry, Department of Chemistry and Applied Biosciences, ETH Zurich, Vladimir-Prelog-Weg 2, CH-8093 Zürich, Switzerland; ∥ Laboratory for Battery Science, PSI Center for Energy and Environmental Sciences, Paul Scherrer Institute, Forschungsstrasse 111, CH-5232 Villigen PSI, Switzerland; ⊥ Department of Materials Science and Engineering, Texas A&M University, College Station, Texas 77843, United States; # Advanced Light Source, 1666 Lawrence Berkeley National Laboratory, Berkeley, California 94720, United States; ∇ Material Measurement Laboratory, 10833 National Institute of Standards and Technology, Gaithersburg, Maryland 20899, United States; ¶ Department of Electrical Engineering, Texas A&M University, College Station, Texas 77843, United States

## Abstract

Neuromorphic computing inspired by mammalian intelligence aims to emulate the nonlinear dynamics of biological neurons and synapses to achieve fast, low-energy, and highly efficient information processing. Brain-inspired computing relies on the design and discovery of materials exhibiting nonlinear current–voltage profiles, frequently underpinned by electronic state transitions, to achieve spiking neurons and dynamically tunable synapses. A signature challenge in the design of artificial neurons is controlling the steepness of first-order transitions in active elements, as abrupt transitions are at risk of driving unstable voltage and temperature oscillations, which result in catastrophic device failure. A critical knowledge gap is the lack of structure–function correlations mapping the composition and atomistic structure of crystalline solids to nonlinear dynamical response characteristics. Here, we address the key question of how modification of atomistic structure correlates with alteration of neuron-like functionality. Constructing oscillator circuits from millimeter-scale single crystals enables high-resolution atomic structure solutions, which we use to demonstrate that the selective positioning of Pb cations modifies charge ordering along a one-dimensional Cu_*x*_V_2_O_5_ framework even at low insertion stoichiometries, thereby providing an atom-precise design parameter for damping first-order transitions. We use temperature-variant X-ray diffraction and X-ray spectroscopy to elucidate the suppression of Cu-ion shuttling based on the precise positioning of Pb ions in seven-coordinated tunnel interstitial sites as the mechanistic basis for transition broadening, thus bridging a critical gap between statistical mechanics and quantum chemical descriptions of phase transitions. Such mechanistic understanding thus paves the way to site-selective modification strategies for modulating the sharpness of first-order transitions, with an exemplary demonstration here in tuning neuronal signal processing.

## Graphical abstract

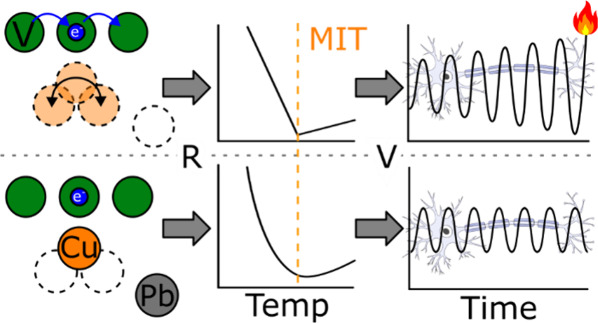

## Introduction

Since about 2010, computing energy efficiency improvements gained from semiconductor device miniaturization have plateaued, signaling an end to Moore’s Law. In conjunction with the enormous increase in computing power demand accompanying the rise of artificial intelligence and “big data”, the failure of Moore’s Law has spurred intense interest in alternative computing architectures.^1^ Neuromorphic computing takes inspiration from the highly efficient information processing performed by the human brain, leveraging materials with nonlinear conductance to emulate neuron and synapse function.^2,3^ The atomistic-scale mechanism of biological neuron spiking has been long understood to involve careful biochemical regulation of transmembrane ion fluxes and concentration gradients (particularly Na^+^ and K^+^).^4^ However, the analogous conductance nonlinearity mechanisms in strongly correlated artificial neuron active materials are not well understood in terms of atoms and electrons. Chemical design strategies for neuromorphic materials thus remain largely ad hoc and empirical.

Many neuromorphic computing materials leverage the conductivity change accompanying a first-order phase transition to emulate neuron-like spiking (Figure 1).^5–8^ Prominent examples include strongly correlated systems such as Peierls–Mott transitions in VO_2_, predominantly Peierls’ transformations of NbO_2_, and charge-transfer insulator transitions in rare-earth nickelates and cobaltates.^6,9–12^ The negative differential resistance that can result from such thermally triggered first-order transitions are particularly well suited for fabricating electrothermal oscillators that mimic the Class I and Class II excitatory spiking behavior of neurons (Figure 1A,B). Such electrothermal oscillators can serve as powerful computing primitives.^13,14^ Indeed, several such oscillators with wraparound circuitry can perform logic and memory functions such as thresholding, amplification, integration, and other complex information processing functions characteristic of neurons with much less entropy dissipation and at much accelerated rates as compared to conventional digital CMOS elements.^14–17^

**Figure 1:**
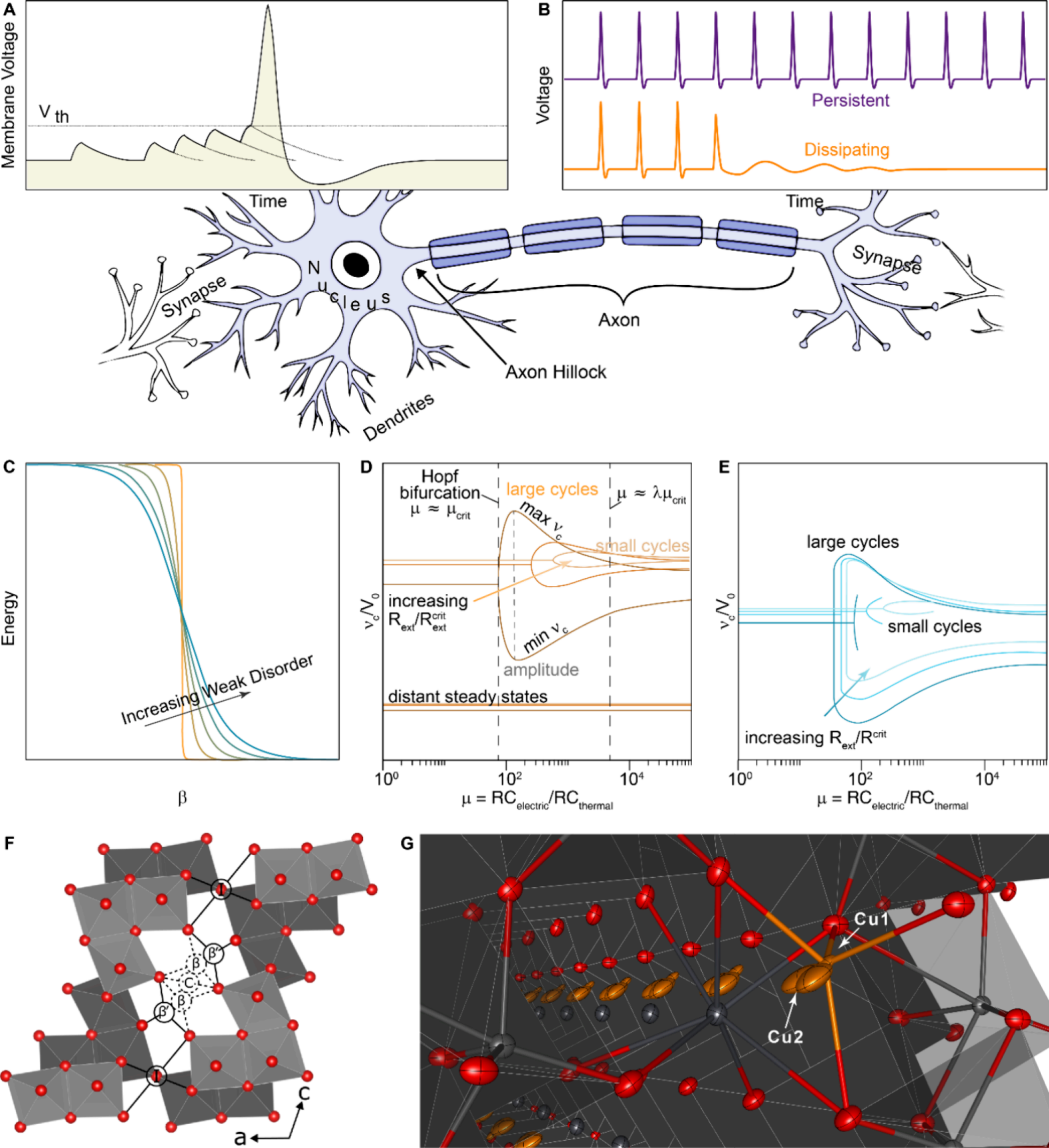
Mapping electronic phase transitions to neuronal and synaptic functionality*.* (A) Illustration of membrane voltage versus time for a single action potential triggered by the accumulation of membrane potential from repeated input spikes. (B) Persistent oscillations and dissipating behavior as two classes of excitability. (C) Possible scenarios of metal–insulator transitions. Abrupt first-order phase transitions (orange color) involve a discontinuous change in a state variable and can be rounded by weak disorder (sky blue color). Electrical-to-thermal time constant ratio (μ)-induced bifurcation plots showing small and large limit cycles, as a function of the external conductance for (D) an abrupt first-order first transition and (E) a highly broadened phase transition. (F) Perspective view of the ζ-V_2_O_5_ tunnel framework showing the diversity of insertion sites available to guest cations, with distinct interstitial sites labeled. The dashed and solid circles indicate sites preferred by lead and copper ions, respectively. (G) Perspective view of the single-crystal structure of the quaternary bronze β-Pb_*x*_/β′-Cu_*y*_V_2_O_5_. The site-filling preferences of Pb and Cu are preserved. The local environments of 7-coordinated Pb in the β-site and 5-coordinated Cu in the β′-site (with pronounced static split-site disorder) are highlighted. Key: light gray = V; red = O; dark gray = Pb; orange = Cu; gray polyhedra = VO_5_/VO_6_.

A key realization that has emerged from analytical frameworks and compact models simulating electrothermal neurons is that abruptly discontinuous first-order phase transitions (Figure 1C) are suboptimal for fabrication of stable oscillators since they can be attracted to distant steady states and/or can yield large unstable oscillations that can fatigue and destroy devices as shown in Figure 1D.^13^ The unstable large-magnitude oscillations derive from substantial temperature variations resulting from abrupt modulations of thermal and electrical conductance across a first-order phase transition under quasi-DC bias.^13,18^ As such, a critical question is how can neuron-like signal processing be modulated through specific atomistic modifications. In other words, *how to tunably broaden first-order phase transitions through site-selective modification while still retaining a sufficient extent of nonlinearity to achieve stable periodic and persistent neuro-emulative oscillations* as sketched in Figure 1E. In the canonical Ehrenfest statistical mechanics view of first-order phase transitions, a thermodynamic order parameter changes discontinuously at the transition point and underpins abrupt modulations of the functional properties of matter.^19,20^ Imry and Wortis suggested that the introduction of weak disorder broadens first-order phase transitions—corresponding to distinctive domains transforming from one phase to another not at a singular critical transition point, but across a smeared band of state variables (e.g., temperature and pressure),^20–22^ each reflective of a modified critical transition.^23,24^ The coinsertion approach demonstrated here represents an atom-precise method for introducing weak disorder and broadening the first-order electronic transition in ternary vanadium oxides, which maps directly to an expanded range of stable and persistent oscillations realized in single crystals.

The tunnel-structed vanadium oxide bronzes β/β′-M_*x*_V_2_O_5_, have received significant attention for their ability to host a variety of guest ions M with wide ranges of stoichiometry *x*, and for their unique combination of physical properties, including highly anisotropic electrical conductivity and propensity to undergo temperature-, voltage- and pressure-driven ion-ordering and electronic structure transitions. In this Article, we describe how a Mott material β′-Cu_*x*_V_2_O_5_ displaying a first-order insulator–metal transformation^25,26^ is modified through precise positioning of Pb-ions in seven-coordinated β-sites adjacent to five-coordinated β′ interstitial sites. Previous work has established that Cu-ion motion mediates charge delocalization along the tunnel-structured ζ-V_2_O_5_ framework by disrupting polaron formation.^25^ The coinsertion of Pb-cations modifies charge-ordering along the ζ-V_2_O_5_ framework even at low insertion stoichiometries and provides a tunable atom-precise design parameter for introducing weak disorder and broadening insulator–metal phase transitions. Such broadening provides a notably broader tolerance window wherein self-sustaining periodic oscillations emulative of biological neurons can be sustained, thereby yielding a much-expanded regime for neuromorphic oscillator-based computing.

Just as single crystals were crucial for the conclusive determination of the structure and function of transmembrane ion channels in biological neurons,^27,28^ single crystals serve here as a distinctive platform to interrogate the atomistic details of weak disorder and to decipher their implications for broadening of first-order phase transitions.^29,25^ Single-crystal-to-single crystal transformations provide insight into changes in lattice periodicity across first-order phase transformations as perturbed by site-selective modification—the placement of atoms in specific positions of the crystal lattice. In this work, we selectively position Pb and Cu ions in specific interstitial sites within the tunnels of the ζ polymorph of V_2_O_5_ polymorph (Figure 1F) and map the site-selective ion positioning determined from high-resolution single-crystal structure solutions to nonlinear dynamical conductance switching (Figure 1G). The results provide critical design rules for atom-precise modification of Mott materials to imbue intrinsic weak disorder providing a critical link between statistical mechanics and quantum chemical perspectives of phase transitions. We demonstrate the utilization of atom-precise damping of first-order transitions in the design of robust neuromorphic devices showing stable and persistent oscillations.

## Results and Discussion

### Site-Selective Positioning of p- and d-Block Cations along Tunnels of ζ-V_2_O_5_

Among known β/β′-M_x_V_2_O_5_ structures only Cu and Fe exhibit the characteristic split-site behavior necessary for the observed ion shuttling and associated polaron oscillation required to underpin nonlinear dynamical transitions.^3,25,30–32^ However, until now, there has been scarce little success in preparing large single crystals of β′-Fe_*x*_V_2_O_5_. Pb was chosen as a large 2+ ion with a tendency to express stereochemical activity and imbue lattice anharmonicity because these factors are likely to interact strongly with Cu-ion motion. Single-crystals of end-member ternary vanadium oxide bronzes β′-Cu_*x*_V_2_O_5_ (*x* = 0.45, 0.53, 0.65)^25,32^ and β-Pb_0.11_V_2_O_5_,^33,34^ as well as new quaternary M_*x*_M′_*y*_V_2_O_5_ bronzes with formulas β-Pb_*x*_/β′-Cu_*y*_V_2_O_5_: (**a**) *x* = 0.028; *y* = 0.28; (**b**) *x* = 0.037; *y* = 0.33; (**c**) *x* = 0.01; *y* = 0.47; (**d**) *x* = 0.08; *y* = 0.33; (**e**) *x* = 0.11; *y* = 0.41) have been obtained from melt growth of powders prepared by solid-state reaction as described in the Experimental Methods section. These compositions were selected on the basis of several factors: (i) prior investigations show that single crystals with Cu stoichiometries above *x* = 0.50 have a high proclivity for metallic behavior and exhibit weaker conductivity transitions, accompanied by little or no structural transformations.^32^ (ii) The relatively large size of the Pb^2+^ ion and proximity of β-sites (hosting Pb) to β′-sites (hosting Cu) within the structure result in an antagonistic relationship between Cu and Pb concentrations (Figure S1). In order to systematically vary Pb concentrations over a wide range of values, Cu concentrations had to be kept low. It should also be noted that some discrepancy exists between the composition of a powder prepared by a solid-state reaction and crystals grown from a melt of this powder, typically resulting in a lower Pb concentration in the melt-grown crystals. These deviations from the intended stoichiometries (e.g., β-Pb_0.028_/β′-Cu_0.28_V_2_O_5_ versus β-Pb_0.025_/β′-Cu_0.33_V_2_O_5_) are clearly delineated and do not modify the overall interpretation. SEM images, energy dispersive X-ray spectra (EDS), and EDS maps of are shown in Figures S2–S5. Figure 2A shows digital photographs of single crystals of ζ-V_2_O_5_, the end-members, and coinserted phases.

**Figure 2:**
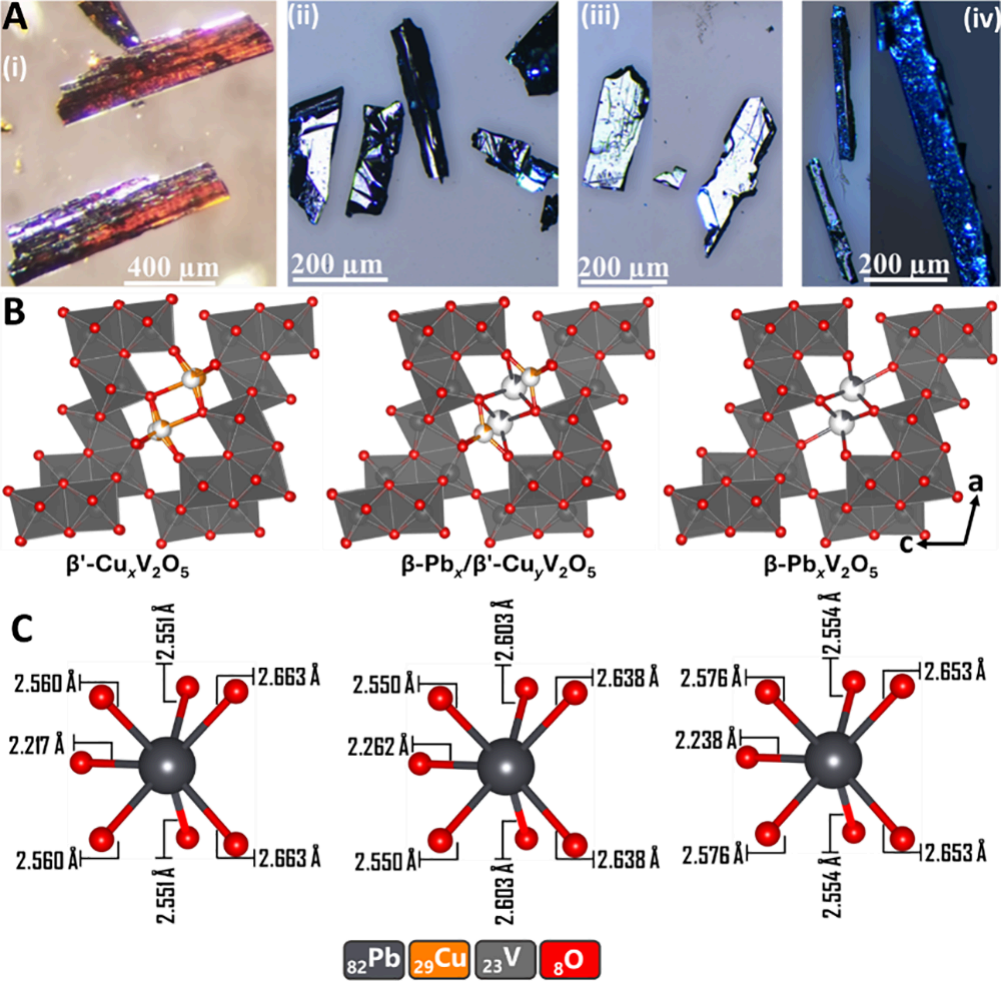
End-member M_*x*_V_2_O_5_ (M = Pb, Cu) and coinserted M_*x*_M′_*y*_V_2_O_5_. (A) Optical images of (i) ζ-V_2_O_5_;^37,71^ (ii) β′-Cu_*x*_V_2_O_5_; (iii) β-Pb_*x*_/β′-Cu_*y*_V_2_O_5_; and (iv) β-Pb_*x*_V_2_O_5_ faceted and colored single crystals. The ζ-V_2_O_5_ crystals are deep orange with an elongated prismatic shape, whereas the β-Cu_*x*_V_2_O_5_ crystals are lustrous black with block-like shapes. In contrast, the Pb/Cu coinserted V_2_O_5_ exhibits a flat shiny plate-like shape, whereas the β-Pb_*x*_V_2_O_5_ crystallizes in long lustrous rod-like shapes. (B) View along tunnels of structures refined from single-crystal X-ray diffraction. The panels show refined crystal structures of β′-Cu_*x*_V_2_O_5_ (left), β-Pb_*x*_/β′-Cu_*y*_V_2_O_5_ (middle) and β-Pb_*x*_V_2_O_5_ (right), highlighting the different site-filling preferences of Cu and Pb along the 1D tunnels of ζ-V_2_O_5_. (C) Comparison of Pb–O coordination for (left) β-Pb_0.01_/β′-Cu_0.47_V_2_O_5_; (middle) β-Pb_0.08_/β′-Cu_0.33_V_2_O_5_; and (right) β-Pb_0.11_/β′-Cu_0.41_V_2_O_5_ illustrating the effects of stereochemical activity of Pb 6s^2^ lone pairs. All refined single-crystal structures obtained for this study have been deposited in the Cambridge Structural Database as noted in the Experimental Methods section with deposition numbers 2373863 (β-Pb_0.11_/β′-Cu_0.41_V_2_O_5_), 2321463 (β-Pb_0.08_/β′-Cu_0.33_V_2_O_5_), and 2321464 (β-Pb_0.01_/β′-Cu_0.47_V_2_O_5_), and 2373861 (β′-Cu_0.53_V_2_O_5_), 2373862 (β′-Cu_0.65_V_2_O_5_).

The growth of high-quality single crystals enables high-resolution atomic structure determination using single-crystal X-ray diffraction (Tables S1–S26). As illustrated in Figure 2B, the extended monoclinic V_2_O_5_ framework for all structures is retained for different stoichiometries and identities of inserted ions. The framework comprises infinite double-layers of edge-sharing VO_6_ octahedra bridged along the *c*-direction by edge-sharing [VO_5_] square pyramids to create infinite tunnels extending down the *b*-direction. The Pb- and Cu-ions fractionate into discrete sites. Pb^2+^-ions (ionic radii of 1.23 Å)^35^ are confined to seven-coordinated β-sites, which are mirrored across the center of the tunnel with incomplete and random occupancy (Figure S1). In contrast, Cu^+^-ions (ionic radii of 0.67 Å)^35^ occupy the five-coordinated β′-site, which is offset from the β-site by 1/2 of a unit cell in the *b*-direction (Figure S1). Cu-ions further exhibit pronounced split-site disorder, where the principal Cu position (labeled Cu1 in Figure 1G) is flanked by a secondary Cu site (labeled Cu2) that is mirrored across the special position.^25^ The strong site preference of Pb- and Cu-ions for the β- and β′-sites, respectively, governed primarily by ionic radii and preferences for local coordination environments (Figure S1),^36^ is maintained in the quaternary bronzes β-Pb_*x*_/β′-Cu_*y*_V_2_O_5_ (Figure 2B, middle). Tables 1 and S1–S26 list the refined lattice parameters of the quaternary β-Pb_*x*_/β′-Cu_*y*_V_2_O_5_ structures and two ternary bronzes of comparable stoichiometries (β-Pb_0.11_V_2_O_5_ and β′-Cu_0.45_V_2_O_5_), contrasted to the “empty” tunnel-structured polymorph, ζ-V_2_O_5_.^37^

**Table 1:** Comparison of β Angles, Activation Energies, *E*_a_, Threshold Voltages, *V*_TH_, Weiss Constant, Θ and Effective Vanadium Reduction, *V*_ER_ Based on Refined Single-Crystal Structures^a^

		*E*_a_, **meV**				
**structure (space group)**	**β (deg)**	**high temp.**	**low temp.**	***V*_TH_, *V***	**(Θ), K**	***V*_ER_ of V^4+^**
ζ-V_2_O_5_ (*C*2/*m*)^b^	110.0					
β-Pb_0.11_V_2_O_5_ (*C*2/*m*)	109.7					0.22
β′-Cu_0.45_V_2_O_5_ (*C*2/*m*)	106.5					0.45
(**a**) β-Pb_0.028_/β′-Cu_0.28_V_2_O_5_ (*C*2/*m*)	107.2	7.80	23.7	30.0		0.34
(**b**) β-Pb_0.037_/β′-Cu_0.33_V_2_O_5_ (*C2/m*)	107.4	6.30	25.6	25.0		0.40
(**c**) β-Pb_0.01_/β′-Cu_0.47_V_2_O_5_ (*C*2/*m*)	106.3	10.7	26.1	36.6	35	0.49
(**d**) β-Pb_0.08_/β′-Cu_0.33_V_2_O_5_ (*C*2/*m*)	107.5	8.8	28.8	118.8	35	0.49
(**e**) β-Pb_0.11_/β′-Cu_0.411_V_2_O_5_ (*C*2/*m*)	107.4	17.7	43.7	13.2	36	0.63

a Listed are the formula of each refined structure and its space group as obtained at 110 K. The quaternary structure has a larger unit cell volume than either ternary structure, owing principally to a slight expansion of *b* and *c* compared to either structure, and an expansion along *a* compared to β′-Cu_0.45_V_2_O_5_ to accommodate Pb-ions. While β-Pb_*x*_V_2_O_5_ bronzes tend to have slightly wider monoclinic β-angles (>107°; here 109.7°) and β′-Cu_*x*_V_2_O_5_ bronzes tend to have more acute monoclinic β-angles that shrinks with increasing Cu stoichiometry (ranging from ca. 107° for Cu_0.30_ to 105° for Cu_0.65_; and here 106.5° for Cu_0.45_).^25^

b Structural and refinement data for ζ-V_2_O_5_ at 110 K obtained from ref 37.

Figure 2C depicts the capped trigonal planar coordination environment around the Pb^2+^ cations in β-Pb_*x*_/β′-Cu_*y*_V_2_O_5_. The Pb^2+^ ions exhibit pronounced stereochemical activity derived from filled 6s^2^ electrons, manifested as a significant shift of the Pb position from the center of the seven-coordinated coordination environment at the β-site. Even for the lowest amount of inserted Pb (0.01 per V_2_O_5_ unit), the large stereochemically active p-block cation props open the tunnel to an extent sufficient to modify the tight Cu-ion interactions with the V_2_O_5_ framework, and its position in the β-site between Cu-ions likely screens Cu–Cu interactions which contribute to shuttling and extended percolation.^38^ Cu still exhibits some split-site disorder characteristic of cation shuttling in end-member β′-Cu_*x*_V_2_O_5_, but the local structure of Cu shows more symmetric polyhedra with increasing Pb concentration (vide infra). Table S27 lists bond distances and bond valence sum (BVS) calculations illustrating comparable reduction of vanadium for the three different cointercalated compounds and the two end members β′-Cu_0.65_V_2_O_5_ and β-Pb_0.33_V_2_O_5_. However, the degree of localization is site-dependent and varies with composition. In the Pb-rich end member, reduction is more pronounced at the V1 and V2 sites, whereas in the Cu-rich end member and the coinserted β-Pb_0.11_/β′-Cu_0.41_V_2_O_5_, reduction is more pronounced at the V1 and V3 sites. As such, coinsertion of Pb-ions provides a means of modifying Cu–V_2_O_5_ ion–lattice interactions, which in turn imbues weak disorder by modulating charge ordering and polaron oscillation mechanisms underpinning conductance switching.

To better understand the local coordination environment around the Pb^2+^ cations in β-Pb_*x*_/β′-Cu_*y*_V_2_O_5_, we examined the total crystal structure of β-Pb_0.05_/β′-Cu_0.33_V_2_O_5_ using synchrotron X-ray pair distribution function (X-PDF) in the temperature range 90–400 K (Figure S6). Figure S6A highlights the evolution of the experimental XPDF data for low *r* peaks (*r* = 1.5–6.0 Å). The features centered at ∼2.5–2.8 Å and 5.0 Å, corresponding to Pb–O and Pb–Pb correlations, respectively, develop noticeable asymmetry upon warming.^39^ This asymmetry suggests the presence of multiple unresolved long and short bond lengths consistent with local atomic displacements that deviate from the average monoclinic *C*2/*m* symmetry.^40^ Furthermore, these peaks exhibit significant thermal broadening and adopt highly non-Gaussian profiles at elevated temperatures, a signature of substantial lattice anharmonicity derived from stereochemical expression of Pb 6s^2^ lone pairs.^41–43^ The electronic structure underpinnings and implications of such stereochemical expression are discussed further in subsequent sections.

Figure S6B–E represents the XPDF plot of β-Pb_0.05_/β′-Cu_0.33_V_2_O_5_ up to 20 Å at selected temperatures (100–400 K), fitted with the monoclinic *C*2/*m* structural model. X-PDF, being a total scattering technique, provides information on both local and average structure.^42^ To better resolve these contributions, we have separated the XPDF data into low *r* peaks (*r* = 1.5–3.2 Å), which reflect the local structure, and high *r* peaks (*r* = 3.2–20 Å), which reflect the average structure. As shown in Figure S6B–E, the high *r* region is well described by the monoclinic *C*2/*m* model, whereas the experimental low *r* peaks arising from Pb–O correlations deviate from the calculated profile for the monoclinic *C*2/*m* structural model, indicating greater emerging asymmetry in the local Pb coordination than is captured by the average structure, which is commonly ascribed to anharmonicity induced as a result of stereochemical expression of Pb 6s^2^ electron lone pairs.^43,44^

The divergence and emerging asymmetry can be quantified by the evolution of the goodness of fit (*R*_w_) value across the temperature series (Figure S6F) adapting a workflow applied to study off-centering resulting from lone-pair expression by Laurita and Seshadri.^43^ The *R*_w_ value for high *r* peaks (*r* = 3.2–20 Å) is in the range 0.084–0.112 as compared to 0.112–0.124 for *r* = (1.5–3.2 Å) at all temperatures. *R*_w_ values for the low *r* peaks (*r* = 1.5–3.2 Å) and high *r* peaks (*r* = 3.2–20 Å) indeed show divergent trends, which reflect the emergent asymmetry in the local structure (Figure S6F). The *R*_w_ values for the average structure, *r* = 3.2–20 Å, vary from 0.112 at 100 K to 0.084 at 400 K, suggesting the average structure retains its overall symmetry with increasing temperature. In contrast, for the local structure (*r* = 1.5–3.2 Å), the *R*_w_ values increase from 0.112 to 0.124 over the same temperature range, indicating increasing deviation of the local structure from the average monoclinic *C*2/*m* structure upon warming (Figure S6F).^43^ This progression toward a more distorted local structure upon warming is characteristic of “emphanisis”, a phenomenon previously observed in Pb chalcogenides, where Pb^2+^ ions become increasingly off-centered with increasing temperature reflecting a signature structural consequence of the stereochemical expression of their 6s^2^ lone-pairs.^40,41^

### Damping First-Order Transitions and Implications for Neuromorphic Function

The end-member ternary compound β′-Cu_*x*_V_2_O_5_ exhibits a sharply discontinuous thermally activated (as well as voltage- and current-induced) first-order metal–insulator transition (Figure 3A).^25^ The conductance switching derives from a combination of polaron oscillation coupled with real-space shuttling of Cu-ions across the two adjacent sites, as sketched in Figure 3B.^25^ In contrast, the p-block end-member compound, β-Pb_*x*_V_2_O_5_, exhibits voltage-induced nonlinear dynamical conductance modulation ascribed to lone-pair repulsions and lattice anharmonicity.^45,46^ Figure 3A plots the temperature dependence of resistance measured for single-crystals of coinserted compounds **a**–**e**. Similar to the end-member β′-Cu_*x*_V_2_O_5_, the low Pb-co-inserted **a** and **b** also exhibit discontinuous—albeit heavily broadened—thermally driven metal–insulator transitions, occurring at slightly lower temperatures of 202 and 205 K, respectively, compared to the end-member β′-Cu_*x*_V_2_O_5_. In contrast, the high Pb coinserted **c–e** compounds exhibit a monotonic rise in resistance with decreasing temperature but with pronounced charge-localization-induced nonlinear modulation of the activation energy (Figures S7 and S8). Activation energies are extracted from the low-temperature regime, where intrinsic transport behavior dominates; (at higher temperatures, defect-dependent scattering mechanisms are predominant). Notably, the conduction activation energy in the low-temperature regime increases with further Pb coinsertion, which is contrary to a static band picture, whereby filling frontier bands would reduce the band gap and increase conductivity. By simple electron counting, β-Pb_0.33_V_2_O_5_ should show the same degree of band filling as β′-Cu_0.66_V_2_O_5_. However, β′-Cu_0.66_V_2_O_5_ shows metallic conductivity,^32^ whereas β-Pb_0.33_V_2_O_5_ is semiconducting, suggesting that Pb incorporation modifies band structure beyond trivial band filling. The observed increase in activation energy for the low-resistance state is attributed to the role of Pb^2+^-ions in enforcing stronger charge localization within the lattice, which stabilizes the insulating state. Such localization likely corresponds to polaronic transport associated with deeper traps, which reflects an increased energy barrier (*E*_a_) for carrier hopping between vanadium sites. As such, site-selective modification based on positioning of Pb-ions in β-sites induces weak disorder that strongly broadens the originally first-order charge-ordering phase transition.

**Figure 3:**
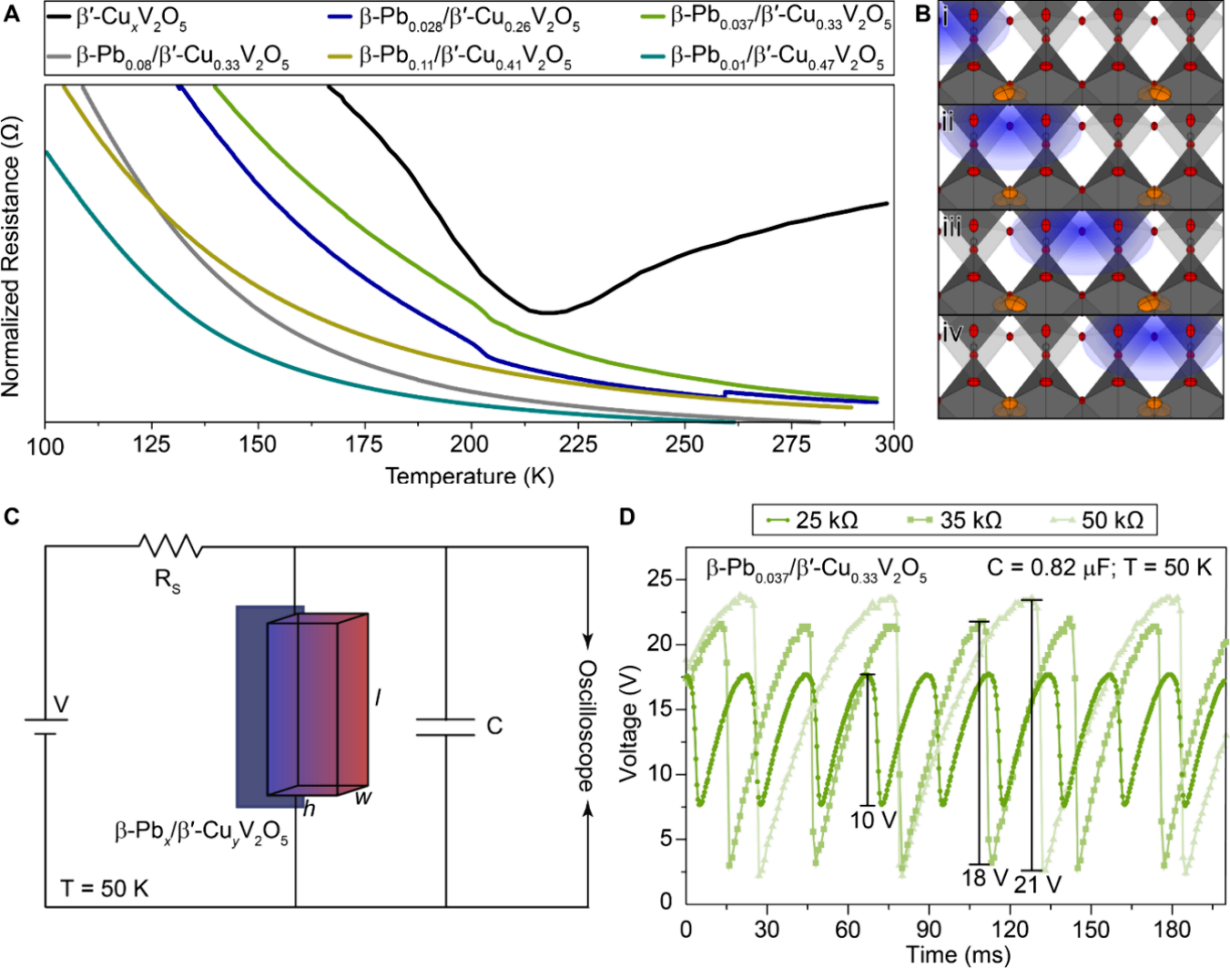
Conductance nonlinearities and characterization of single crystal oscillators. (A) Resistance versus temperature measured for single crystals of β′-Cu_*x*_V_2_O_5_, β-Pb_0.028_/β′-Cu_0.28_V_2_O_5_, β-Pb_0.037_/β′-Cu_0.33_V_2_O_5_, β-Pb_0.08_/β′-Cu_0.33_V_2_O_5_, β-Pb_0.11_/β′-Cu_0.41_V_2_O_5_ and β-Pb_0.01_/β′-Cu_0.47_V_2_O_5_.The discontinuity at 262K in β-Pb_0.028_/β′-Cu_0.28_V_2_O_5_ is a measurement-related effect from the source measurement unit (SMU). (B) Idealized depiction of polaron shuttling mechanism in β′-Cu_*x*_V_2_O_5_; polaron hopping along V–O–V chains is facilitated by the motion of copper ions from one split site (i) through the central beta-prime site (ii) to the other split site (iii). (C) Oscillator circuit diagram used to generate self-sustaining oscillations. (D) Stable voltage oscillations measured across a β-Pb_0.037_/β′-Cu_0.33_V_2_O_5_ single crystal oscillator with *V* = 45 V, *C* = 0.82 mF, and *R* = 25, 35, and 50 kΩ, configured as depicted in (C).

In addition to suppressing the temperature-induced conductance modulation, Pb coinsertion imbues a voltage-induced transition reminiscent of β-Pb_*x*_V_2_O_5_, as shown in Figure S9 and Table 1. Figure S9A–D shows voltage-triggered conductance switching observed for millimeter-sized single crystals. With increasing voltage an abrupt, reversible, and hysteretic increase in current is observed, which corresponds to the transition to a higher conducting state. Hysteretic loops as a function of temperature shown in Figure S9A–D confirm that Pb-co-insertion introduces weak disorder while still retaining the underlying first-order phase transition.^22,47^

Figure 3D illustrates the time-dependent output voltage of an oscillator circuit (Figure 3C, shows the circuit diagram) fashioned out of an entire single crystal of β-Pb_0.037_/β′-Cu_0.33_V_2_O_5_. Figure S10 shows time-dependent output voltages for single crystals **a** and **e**. When supplied with a constant DC bias and at series resistances tuned for each crystal (7, 8, and 9 kΩ for β-Pb_0.028_/β′-Cu_0.28_V_2_O_5_, Figure S10A), a parallel 1.0 μF capacitor charges until it reaches a threshold voltage at which the power dissipated through the crystal induces Joule heating that switches it into its conductive state; the capacitor discharges through the crystal; cooling based on Newtons’ laws reverts the active element to its high resistive state, initiating the cycle anew.^48^

Figures 3D and S10B show the implications of broadening the first-order phase transition for neuromorphic oscillators. The low-Pb materials exhibiting more pronounced first-order phase transitions show a broader range of oscillation frequencies and voltages but are constrained to narrower range of circuit capacitance where stable self-sustaining oscillations can be obtained. With increasing concentration of inserted Pb-ions more expansive regimes of self-sustaining dynamic oscillations can be accessed across broader windows of overall circuit capacitance. Large-cycle oscillations that can bring about catastrophic failure (Figure 1D)^13^ are furthermore substantially muted with increasing concentration of Pb-ions and broadening of the first-order phase transition (Figure 3D).^17,14,49^

In contrast to β′-Cu_*x*_V_2_O_5_ and ε-Cu_*x*_V_2_O_5_ with abruptly discontinuous first-order phase transitions that yield both narrowly scoped and large-magnitude oscillations that are prone to catastrophic thermal damage and fatigue,^29,25^ the ability to introduce weak disorder by Pb coinsertion opens up a substantially greater design space with “edge-of-chaos” characteristics for sustaining persistent periodic small-cycle oscillations as needed for robust neuro-emulative information processing.

In order to extrapolate the effects of weak disorder on neuromorphic circuit performance, electro-thermal oscillator simulations were conducted based on a compact circuit model.^50^

Figures 4A and S11 plot the oscillation frequency versus circuit capacitance relationship modeled for β-Pb_*x*_/β′-Cu_*y*_V_2_O_5_ oscillators. Increasing Pb content increases the minimum capacitance required to sustain oscillations and yields a broader range of capacitive coupling for device implementation (note that broadening the conductance transition by Pb coinsertion has little intrinsic effect on the oscillation frequency). The corresponding voltage amplitude versus capacitance relationships in Figure 4B show that Pb coinsertion further drastically reduces the oscillator’s voltage amplitude. In practice, this reduces both the required supply voltage and the likelihood of catastrophic electrical or thermal excursions (compare with Figure 1D).^13^ Artificial neurons constructed from only two coupled oscillator elements display a wide array of complex spiking behaviors depending on the difference in frequency and amplitude of the individual oscillators.^6^ Pb coinsertion allows these characteristics to be tuned by intrinsic changes to the active material, yielding a rich design space, far beyond the narrow singularities where edge-of-chaos is typically manifested.

**Figure 4:**
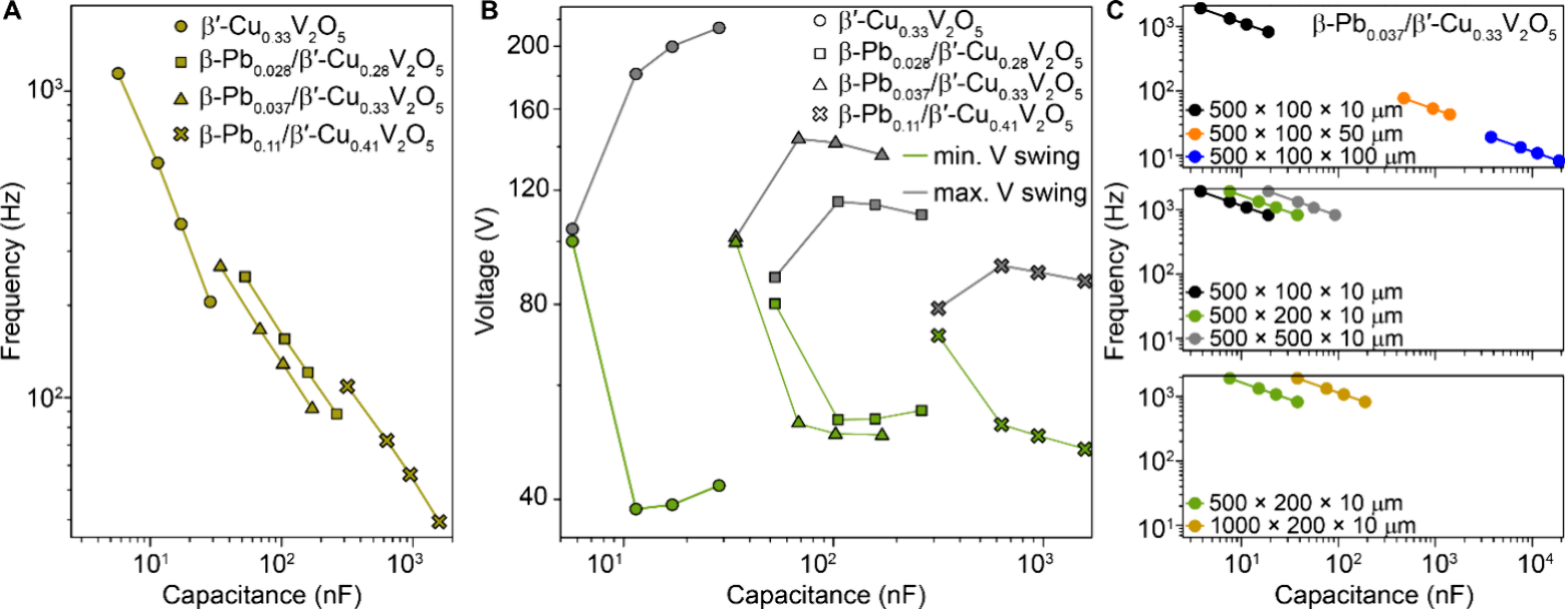
Compact models of single crystal electrothermal oscillators*.* (A) Frequency versus total circuit capacitance predicted by an electrothermal oscillator simulation for β′-Cu_*x*_V_2_O_5_, β-Pb_0.028_/β′-Cu_0.28_V_2_O_5_, β-Pb_0.037_/β′-Cu_0.33_V_2_O_5_, and β-Pb_0.11_/β′-Cu_0.41_V_2_O_5_ crystals with dimensions of 500 × 100 × 50 μm^3^ at 120 K and for the experimental results in (Figures 2A**,** [Fig fig3]D, and S10). (B) Minimum and maximum voltage swings as a function of capacitance for the crystals in (A) predicted by the electrothermal oscillator simulation. (C) Frequency vs total circuit capacitance predicted by an electrothermal oscillator simulation at 120 K for β-Pb_0.037_/β′-Cu_0.33_V_2_O_5_ crystals with indicated length × width × thickness dimensions. The top, middle and bottom panels show the effect of varying thickness, width, and length, respectively.

The resistance vs temperature measurements (Figure 3A) used to inform the compact model were performed on millimeter-scale single crystals. Changes in simulated oscillator behavior with dimension reduction, corresponding to scaling behavior, are represented in Figure 4C, which shows the frequency vs capacitance relationship predicted for crystals with varying sizes (length along electrical conduction path × width × height along thermal conduction path, see Figure 3C).^29^ The oscillator frequency and minimum capacitance are evidently most sensitive to crystal height (which determines heat capacity but not thermal contact area), indicating that thermal conduction plays a strong role in dictating the oscillator behavior of such electrothermal neurons.^13^ Overall, these simulations predict that device scaling in addition to compositional modification provides a means of tuning oscillation frequency and decreasing circuit capacitance requirements to the benefit of computing speed and efficiency.

### Mechanistic Origins of Weak Disorder Imbued by Site-Selective Positioning of Pb Ions in Interstitial Sites

Temperature-variant synchrotron powder X-ray diffraction (XRD) measurements have been performed to examine how the lattice periodicity evolves as a function of temperature upon coinsertion of Pb-ions. The stacked temperature-dependent powder X-ray diffractograms are plotted in Figure 5A–F and S12. Sequential Le Bail refinements were conducted for each sample in a temperature range from 90 to 395 K (Tables S28–S39).

**Figure 5:**
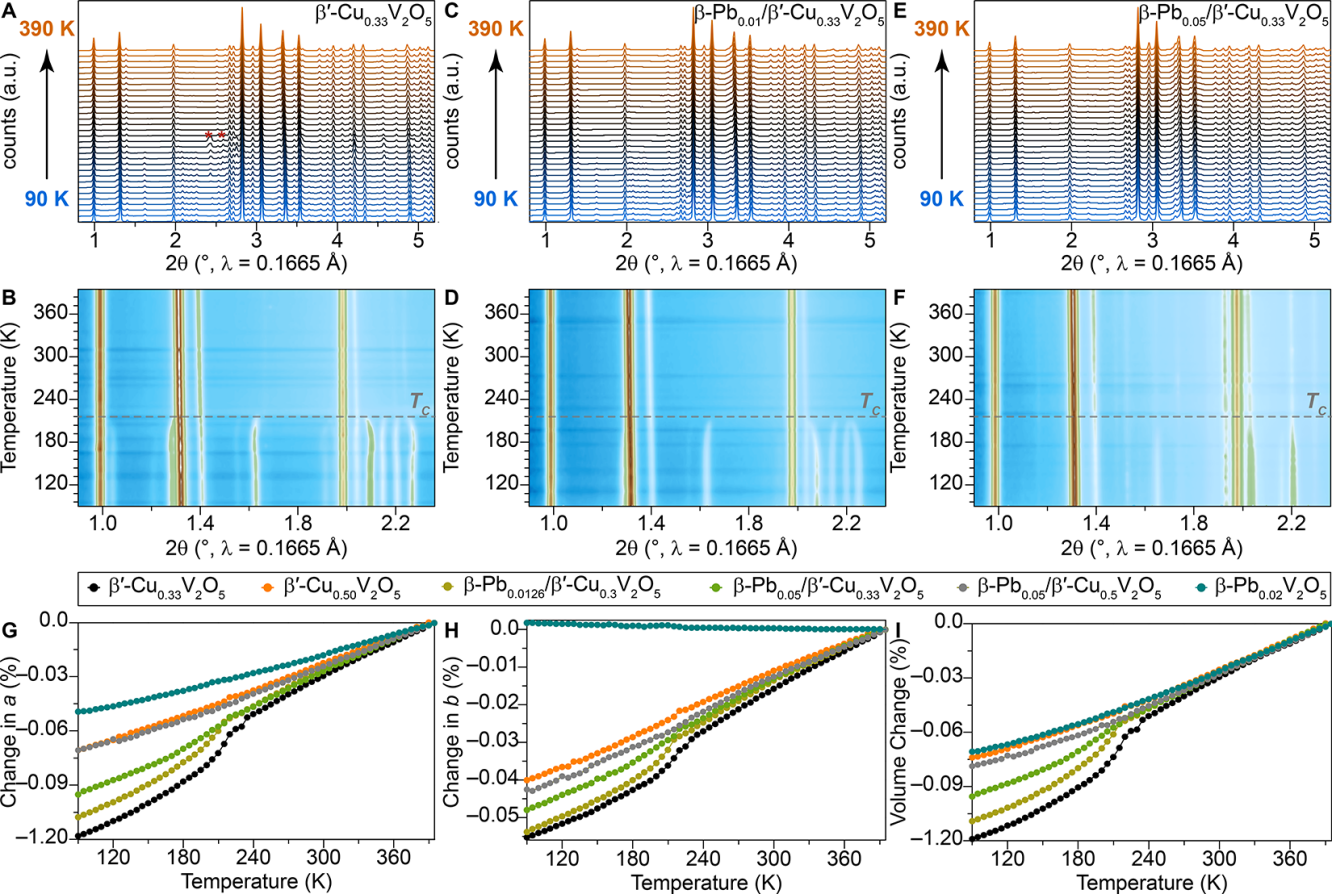
Structural Origins of Conductance Nonlinearities in Single Crystals of β-Pbx/β'-CuyV2O5. Synchrotron X-ray diffraction contour color plot of a selected 2θ region, as a function of temperature (90−395K) collected using a ramp rate of 1 K/min for (A, B) β'-Cu_0.33_V_2_O_5_, (C, D) β-Pb_0.05_/β'-Cu_0.33_V_2_O_5_, and (E, F) β-Pb_0.2_V_2_O_5_. A weak reflection marked with an asterisk (*) originates from ice. Evolution of (G) volume, (H) β angle, (I) a lattice parameter, as a function of temperature for β'-Cu_0.33_V_2_O_5_, β'-Cu_0.50_V_2_O_5_, β-Pb_0.0126_/β'-Cu_0.3_V_2_O_5_, β-Pb_0.05_/β'-Cu_0.33_V_2_O_5_, β-Pb_0.05_/β'-Cu_0.5_V_2_O_5_ and β-Pb_0.2_V_2_O_5_. Notably, the temperature-dependent powder X-ray diffractograms of β'-Cu_0.33_V_2_O_5_ reveal the presence of a low-temperature superstructure at 95K that gradually disappears by ≈210K. The reflections are tentatively assigned to an incommensurate superstructure with a modulation vector q_m_ = (0, 0.3085, 0) (Figure S15). Increasing the Pb stoichiometry to 0.01 and 0.05 following β-Pb_0.01_/β'-Cu_0.33_V_2_O_5_ and β-Pb_0.05_/β'-Cu_0.33_V_2_O_5_ gradually diminishes the intensity of the modulation satellites, following a similar trend as shown by the temperature-induced conductivity transition (Figure S15).

The normalized refined lattice parameters, β angle, and unit cell volume as a function of the temperature of each system are plotted in Figures 5G–I and S13, and S14. The inclusion of both Cu- and Pb-ions in interstitial sites expands the tunnels of the ζ-V_2_O_5_ framework. Each material shows an overall volumetric thermal expansion; the magnitude of the thermal expansion decreases with increasing Pb concentration. This overall volumetric thermal expansion arises from thermal expansion along *a* (Figure 5I) and *c* (Figure S13A) but competes with a subtle negative thermal expansion along *b* (Figure S13B). Discontinuous first-order phase transitions are observed for end-member β′-Cu_0.33_V_2_O_5_ and low-Pb coinserted, **a** and **b**, between 210 and 230K (Figures 5G–I and S13A,B) coincident with the electronic phase transition observed in conductivity measurements in Figure 3A.

Compositions that display discontinuous changes in lattice parameters characteristic of first-order phase transitions also present new reflections appearing below the transition temperature, which we interpret as structural modulation satellites (Figure S15). The modulated structures are expected to arise from Cu-ion ordering along *b* because of their relatively large intensity (thus excluding small atomic displacements from electron localization), in agreement with prior interpretations.^32^ Previous investigations have concluded that the low-temperature insulating state in β′-Cu_*x*_V_2_O_5_ arises from a loss of Cu-ion shuttling, which we associate with ordering of Cu-ions. With increasing Pb^2+^ stoichiometry, the tunnels of the ζ-V_2_O_5_ framework are expanded, and the lattice parameters and cell volume/angle are observed to change smoothly with temperature at higher Pb concentrations. In Figure S16, β′-Cu_*x*_V_2_O_5_ shows a greatly increased split-site occupancy at low temperature, suggesting that Cu-ion displacements driven by Cu–Cu interactions within the tunnels become “frozen” in the insulating state. The presence of Pb in the seven-coordinated β-sites suppresses Cu split-site occupancy at low temperature, indicating that Pb broadens the structural and electronic transitions by interrupting Cu–Cu interactions by screening electrostatic repulsion and/or by altering the tunnel geometry to destabilize the split sites (thus modifying Cu-ion/lattice interactions).

The temperature dependences of the magnetic susceptibility of β′-Cu_*x*_V_2_O_5_ and β-Pb_*x*_/β′-Cu_*y*_V_2_O_5_ are shown in Figures 6A–C and S17. Paramagnetic behavior is observed across the entire temperature range for β′-Cu_0.53_V_2_O_5_ (Figure 6A) with the measured susceptibility increasing with decreasing temperature because of reduced thermal agitation. Magnetization measurements were also performed for various β′-Cu_*x*_V_2_O_5_ (0.55 ≤ *x* ≤ 0.66) compositions. A distinctive modulation from antiferromagnetism dominant to paramagnetism dominant behavior is observed only for a “fully stuffed” β′-Cu_0.66_V_2_O_5_ (Figures 6B and S17A) where Cu-ion oscillation is likely strongly suppressed by filling of available interstitial sites, thereby enforcing strong charge localization. In contrast, the χ­(*T*) plots of β-Pb_0.11_/β′-Cu_0.41_V_2_O_5_, β-Pb_0.08_/β′-Cu_0.33_V_2_O_5_, and β-Pb_0.01_/β′-Cu_0.47_V_2_O_5_ (Figures 6C and S17B,C) show a ferromagnetic (FM) transition at a temperature of approximately 35 K. Below *T*_C_, the χ (T) displays a weak bifurcation between the zero field-cooled (ZFC) and field-cooled (FC) curve suggesting potential coexistence of FM and AFM interactions (see also Figure S18).^51–55^

**Figure 6:**
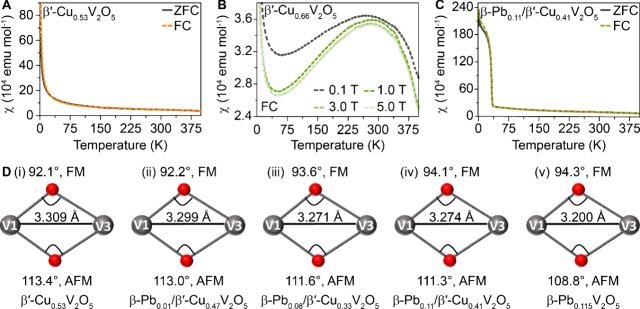
Magnetic transitions in single crystals of β-Pb_*x*_/β′-Cu_*y*_V_2_O_5_. Temperature dependence of magnetic susceptibility measured using a superconducting quantum interference device at an external magnetic field of 0.1 T between ZFC and FC processes in the range of 2–400 K for (A) β′-Cu_0.53_V_2_O_5_, (B) β′-Cu_0.66_V_2_O_5_, and (C) β-Pb_0.11_/β′-Cu_0.41_V_2_O_5_ between 2 and 400 K at an applied field of 0.1 T. (D) Principal superexchange interaction as a function of the V–O–V bond angles (deg) in (i) β**′**-Cu_0.53_V_2_O_5_, (ii) β-Pb_0.01_/β′-Cu_0.47_V_2_O_5_, (iii) β-Pb_0.08_/β′-Cu_0.33_V_2_O_5_, (iv) β-Pb_0.11_/β′-Cu_0.41_V_2_O_5_, and (v) β**′**-Pb_0.115_V_2_O_5_.

Figure 6D displays the most relevant V(1)–O(8)–V(3) superexchange interactions. A lone-pair distortion engendered by coinsertion of Pb-ions bearing stereochemically active electron lone pairs modulates oxide-mediated superexchange between the V(1)­O_6_ octahedral and adjacent V(3)­O_5_ square pyramid chains and underpins the observed ferromagnetism (Figure S18). As such, the magnetic measurements illustrate that (i) the specific charge localization patterns underpinning nonlinear dynamical conductance switching involve reduction of V 3d states of the V_2_O_5_ framework and (ii) by dint of their structural distortion of the host framework, Pb-ions modify the ability of Cu-ions to enforce charge localization, enforcing a ferromagnetic insulating state at low temperatures (vide infra).

We have further examined charge localization on the V_2_O_5_ framework using X-ray absorption and emission spectroscopies, which are correlated to local structure using extended X-ray absorption fine structure spectroscopy. Figure 7A plots V 2p and O 1s core-level hard X-ray photoemission (HAXPES) spectra measured at an excitation energy of 2 keV. In the V 2p_3/2_ region, ζ-V_2_O_5_ shows a singular feature corresponding to nominally pentavalent vanadium. A distinct shoulder, indicative of nominally tetravalent vanadium centers, is observed for the coinserted samples. V L_II/III_- (2p → 3d) and O K-edge (1s → 2p) X-ray absorption near edge structure (XANES) spectra are shown in Figure 7B and probe the orbital character of the conduction band in these compounds. At the V L_III_-edge,^56–58^ the diminution in intensity of the V 3d_*xy*_ feature at the V L_III_ edge for the β-Pb_*x*_/β′-Cu_*y*_V_2_O_5_ compounds is a result of Pauli blocking and corroborates that vanadium reduction as a result of Cu- and Pb-insertion involves the occupation of low-lying V 3d_*xy*_ states at the conduction band edge.^59^

**Figure 7:**
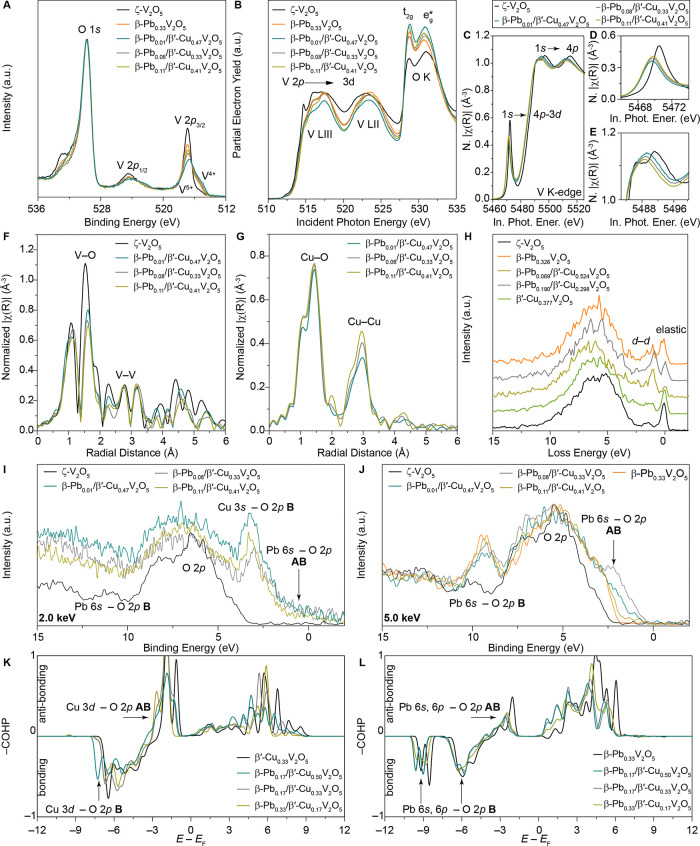
Stereochemically active lone pairs and their effects on local electronic structure. (A) Core-level O 1s and V 2p HAXPES spectra collected at an incident photon energy of 2.0 keV for β-Pb_*x*_/β′-Cu_*y*_V_2_O_5_, ζ-V_2_O_5_, and β-Pb_0.33_V_2_O_5._ (B) V L- and O K-edge XANES spectra acquired for β-Pb_*x*_/β′-Cu_*y*_V_2_O_5_, ζ-V_2_O_5_, and β-Pb_0.33_V_2_O_5_. Distinct features corresponding to transitions from V 2p core states to V 3d (3d_*xy*_, 3d_*xz*,*yz*_, and 3d_**x* ^2^*__–**y* ^2^*_) states split by crystal field splitting in the distorted octahedral (VO_6_) and adjacent (VO_5_) square pyramid coordination geometries. (C) V K-edge XANES spectra of β-Pb_*x*_/β′-Cu_*y*_V_2_O_5_ and ζ-V_2_O_5_ as reference. The expanded region of the (D) pre-edge and (E) white-line features in C, respectively. The energy shift to lower binding energies becomes more pronounced with increasing Pb stoichiometry, as shown in C­(ii). Additionally, the pre-edge absorption arising from transitions from V 1s core levels to hybrid V 4p–3d states that become accessible as a result of covalency and distortions from octahedral symmetry is modified in peak position and relative intensity upon coinsertion of Pb- and Cu-ions.^42,43^ The pre-edge absorption feature of β-Pb_*x*_/β′-Cu_*y*_V_2_O_5_ is less pronounced than in ζ-V_2_O_5_, suggesting more symmetric VO_*x*_ coordination upon Pb and Cu coinsertion. This increased symmetry becomes more evident as Pb stoichiometry decreases (C­(i)). *k* ^3^-Weighted Fourier transforms of (F) V K-edge EXAFS spectra. (G) Cu K-edge EXAFS spectra of β-Pb_*x*_/β′-Cu_*y*_V_2_O_5_ and ζ-V_2_O_5_ as reference. Focusing on the first coordination shell of F, a model considering one short V–O bond, and one long V–O_trans_ bond of VO_6_ is used to fit EXAFS spectra and compare the local V environments. The fitted *R*-space EXAFS spectra are shown in Figure S20A–C; fitting parameters related to the major scattering paths are provided in Tables S40 and S41. (H) RIXS spectra collected at the V L-edge for β-Pb_*x*_/β′-Cu_*y*_V_2_O_5_, ζ-V_2_O_5_, and β-Pb_*x*_V_2_O_5_ and β′-Cu_*x*_V_2_O_5_ at 514.6 excitation energy on an energy-loss scale. Overlay of valence band HAXPES spectra collected for β-Pb_*x*_/β′-Cu_*y*_V_2_O_5_, ζ-V_2_O_5_, and β-Pb_0.33_V_2_O_5_ at incident photon energies of (I) 2.0 and (J) 5.0 keV. COHP analyses of (K) Cu–O interactions in β-Pb_*x*_/β′-Cu_*y*_V_2_O_5_, and β′-Cu_0.33_V_2_O_5_; (L) Pb–O interactions in β-Pb_*x*_/β′-Cu_*y*_V_2_O_5_ and β-Pb_0.33_V_2_O_5_. COHP Bonding interactions between two species are negative on the vertical axis, whereas antibonding interactions are positive on the vertical axis.

XANES and extended X-ray absorption fine structure (EXAFS) spectroscopy data have also been collected at the V K-edge (Figure 7C–E). Figure 7C contrasts V K-edge XANES spectra.^60,61^ The dipole-allowed white-line 1s → 4p absorption, which is sensitive to oxidation state, is shifted to lower binding energies upon Pb and Cu coinsertion, suggesting a reduction in the formal vanadium oxidation state. To investigate the local vanadium environment, the *k* ^3^-weighted Fourier-transformed V K-edge EXAFS data are contrasted in Figure 7F. In the case of ζ-V_2_O_5_, the fitting result (Figure S19) shows that VO_6_ octahedra have one short V=O (vanadyl) bond at ca. 1.56 Å transposed against a long bond at ca. 2.51 Å (Table S40). However, upon Pb and Cu coinsertion, the fitting results in Figure S20A–C show an expansion of the vanadyl bond (1.60–1.63 Å) and concurrent contraction of the opposite V–O bond (2.29–2.30 Å) in β-Pb_*x*_/β′-Cu_*y*_V_2_O_5_, which corroborates the relationship between vanadium oxidation state and V–O bond length and thereby the increased VO_6_ octahedral symmetry upon Pb and Cu coinsertion.^25^ This relationship likely underlies the sensitivity of charge carrier mobility within the V_2_O_5_ framework to the behavior of guest ions in interstitial sites within the tunnels, as guest ion motion distorts intervening vanadyl bonds.

Cu K-edge XANES spectra are shown in Figure S21A and^25,62^ *k* ^3^-weighted Fourier-transformed Cu K-edge EXAFS spectra are shown in Figure 7G. The Cu–O fitting results (Figure S21B–D and Table S41) show Cu(1)–O bond lengths (1.91–1.95 Å) characteristic of five-coordinated monovalent Cu centers.^63^ It is noteworthy that the distribution of both Cu(1)–O and Cu(2)–O bond lengths become symmetrical with increasing Pb stoichiometry.^64^ An analogous fitting procedure was performed for the Pb L_3_-edge; the resulting Pb–O coordination shell fits, and corresponding structural parameters are presented in Figures S22 and S23 and Table S42, respectively. The inferred Pb–O distances for β-Pb_*x*_/β′-Cu_*y*_V_2_O_5_ are consistent with values reported for other divalent lead oxides with stereochemically active lone pairs such as PbO,^65,64^ and PbGa_2_O_4_.^64^

Taken together, the high-resolution single-crystal structure solutions in Figure 2 and spectroscopic characterization in Figure 7 indicate (i) preservation of the ζ-V_2_O_5_ tunnel framework upon coinsertion; (ii) site-selective positioning of divalent Pb and monovalent Cu in seven- and five-coordinated β and β′ sites, respectively; (iii) off-centering of Pb cations as a result of the stereochemical expression of its 6s^2^ lone pairs; (iv) dependence of vanadium reduction on the motion of interstitial cations, mediated by intervening vanadyl bonds; and (v) symmetrization of Cu local coordination with propping open of the tunnel and increasing incorporation of Pb-cations. As such, the weak disorder of structural and electronic first-order phase transitions of β′-Cu_*x*_V_2_O_5_ induced by Pb-ion coinsertion derive from the structural distortions to the host framework induced by the stereochemical expression of Pb 6s^2^ lone-pairs and the modulation of Cu split-site interactions, thereby constraining the cation shuttling and charge ordering mechanism.^25^

Resonant inelastic X-ray scattering (RIXS) spectra were acquired for ζ-V_2_O_5_, β-Pb_*x*_V_2_O_5_, β′-Cu_*x*_V_2_O_5_, and β-Pb_*x*_/β′-Cu_*y*_V_2_O_5_ single crystals. Figures 7H and S24 display the RIXS spectra acquired at the V L_3_-edge, plotted on an energy-loss scale alongside the corresponding XANES spectrum. When excited at 514.6 eV, a broad feature appears approximately at 6 eV ascribed to charge-transfer excitations between V 3d and O 2p hybrid states and shifts from 6 to 10 eV loss energy with increasing excitation energy (Figure S24).^66^ Additionally, an inelastic feature at 1.2 eV energy loss emerges in the spectra excited near the V 3d_*xy*_ resonance, which is attributed to on-site d–d electron transitions from occupied V d_xy_ states to unoccupied V d_xz_ and V d_yz_ states.^25^ This d–d electron transition is weaker in β′-Cu_*x*_V_2_O_5_, because of delocalization of V 3d_*xy*_ electrons resulting from the shuttling of the Cu-ions across the two adjacent V sites. However, upon Pb coinsertion (β-Pb_*x*_/β′-Cu_*y*_V_2_O_5_ and β-Pb_*x*_V_2_O_5_), the transition becomes stronger, which reflects enhanced electron localization on the V 3d_*xy*_ orbitals, which enforces a more insulating ground state.

### Stereochemical Expression of Pb Ions and Resulting Modulation of Ion–Lattice Interactions

Energy-variant valence-band HAXPES measurements have been performed to examine the stereochemical activity of the divalent lead ions. Figures 7I,G contrast valence-band HAXPES spectra measured at incident photon energies of 2.0 and 5.0 keV, respectively. The photoionization cross-sections for subshells with higher orbital angular momentum are diminished more sharply as a function of incident photon energy, enabling variable energy HAXPES to be used as a probe of orbital character. Accordingly, HAXPES spectra obtained at higher incident photon energies more prominently feature orbital contributions from subshells with lower angular momentum values.^67–71^ In our current context, upon varying the incident photon energy in valence-band photoemission experiments, states with considerable Cu 3d character are most pronounced in intensity at low incident energies (2.0 keV), whereas Pb 6s^2^-derived states are more prominent at higher incident energies (5.0 keV).^6,15^

A direct comparison of valence-band HAXPES spectra between ζ-V_2_O_5_, and β-Pb_*x*_/β′-Cu_*y*_V_2_O_5_ reveals three distinct spectroscopic features: a feature centered at ca. 10 eV binding energy is evident for β-Pb_*x*_/β′-Cu_*y*_V_2_O_5_ but absent in ζ-V_2_O_5_; this feature is most pronounced at 5 keV and is ascribed to bonding (B) Pb 6s^2^ states hybridized with O 2p states. Two additional features are observed at the valence band maximum of β-Pb_*x*_/β′-Cu_*y*_V_2_O_5_ in comparison to ζ-V_2_O_5_. The 2 keV spectra show pronounced Cu 3d states located right below the Fermi level at 3.2 eV, whereas filled antibonding (AB) Pb 6s^2^–O 2p states corresponding to stereochemically active lone pair are observed at 1.9 eV right below the Fermi level.^25,45^ The presence of this anion-hybridized bonding state and the corresponding antibonding state at the valence band edge provides direct evidence that the Pb 6*s* electrons are not inert, but instead are (strongly) hybridized with O 2p states, consistent with stereochemical expression. The energy separation between the bonding (B) and antibonding (AB) states is commonly used in the HAXPES literature as a useful metric for comparing lone-pair expression across related systems based on the strength of anion hybridization.^72,68,73,74^ Table S43 shows that the measured value of 8.1 eV for β-Pb_0.11_/β′-Cu_0.41_V_2_O_5_ is comparable to that observed in other Pb-, Tl-, and Sn-compounds such as PbVO_3_Cl and δ-Pb_0.5_V_2_O_5_ known to manifest stereochemically active lone pairs.^39,45,51,72^

To aid spectral interpretation, first-principles DFT calculations have been performed starting with structures obtained from high-resolution single-crystal X-ray diffraction. Figure S25 illustrates the total and atom-projected density of states for β-Pb_0.33_V_2_O_5_ and β-Pb_*x*_/β′-Cu_*y*_V_2_O_5_. In both compounds, the valence band maximum (VBM) exhibits electronic states arising from Pb orbitals. The presence of Pb states at the VBM underscores the role of stereochemically active lone pairs in defining the Fermi surface in coinserted β-Pb_*x*_/β′-Cu_*y*_V_2_O_5_ compounds. Crystal Orbital Hamilton Population (COHP)^52,75^ analyses further aid spectral interpretation and delineate the extent and modes of hybridization (Figure 7K,L).^68,76,77^

A molecular orbital diagram derived from the valence band measurements and COHP calculations is plotted in Figure S26. The hybridization of Pb 6s stereoactive lone pair states with O 2p states results in the formation of Pb 6s–O 2p bonding (B) at approximately 10 eV and Pb 6s–O 2p antibonding (AB) states at the VBM (Table S43 indicates Δ*E* of 8.1 eV). A second-order Jahn–Teller distortion stabilizes the hybridization of the lone pair *n*s^2^-states with ligand p states.^68^ The break in symmetry allows further hybridization of Pb 6s–O 2p AB states with unoccupied Pb 6p states in the conduction band, resulting in the formation of occupied antibonding states labeled as Pb 6s, 6p–O 2p lone pair (LP) states. COHP analysis of β-Pb_*x*_/β′-Cu_*y*_V_2_O_5_ also suggests some hybridization of Cu 3d with O 2p, resulting in the formation of Cu 3d–O 2p B and Cu 3d–O 2p AB states as shown in Figure 7K.^38^ As such, HAXPES and electronic structure calculations corroborate the strong stereochemical activity of Pb 6s^2^ lone pairs, which engenders a midgap state at the top of the valence band and enforces charge localization along the V_2_O_5_ framework. Charge carrier injection from this midgap state has been proposed to contribute to the voltage-driven nonlinear dynamical conductance transition in end-member β-Pb_0.33_V_2_O_5_.^45,46^

## Discussion

Using distinctive single crystals as platforms for high-resolution structure determination, electronic structure spectroscopy, and measurements of single-crystal neuronal oscillators, we seek to determine the means by which Pb coinsertion broadens the temperature-driven metal–insulator transition in β-Pb_*x*_/β′-Cu_*y*_V_2_O_5_. We will first consider the transition mechanisms of the end-members β′-Cu_*x*_V_2_O_5_ and β-Pb_*x*_V_2_O_5_.

Goodenough proposed an early conduction model for β-M_*x*_V_2_O_5_ based on structural features and trends in conduction activation energy and Seebeck coefficient. In brief, small polarons hop from one V2 site to another along the crystallographic *b* direction via collective V 3d bands which increase in domain size with cation insertion.^78^ More recent investigations have established that the insulating state in β′-Cu_*x*_V_2_O_5_ arises from electron localization stabilized by the formation of small polarons, with favorable electrostatic contributions from stationary Cu-ions in β′ sites (green arrows in Figure 8, bottom-left panel).^25^ Above a critical temperature, the motion of Cu-ions destabilizes the small polaron, freeing V 3d electrons for transport (Figure 8, top-left panel). The abrupt change in lattice parameters and formation of a superstructure shown in Figure 5 are consistent with the “freezing in” of Cu-ion displacements during polaron formation. The relationship between vanadium redox and vanadyl bond length, derived from EXAFS results (Figure 7F and related discussion), corroborates this interaction between Cu-ion motion and electron localization on vanadium sites.

**Figure 8:**
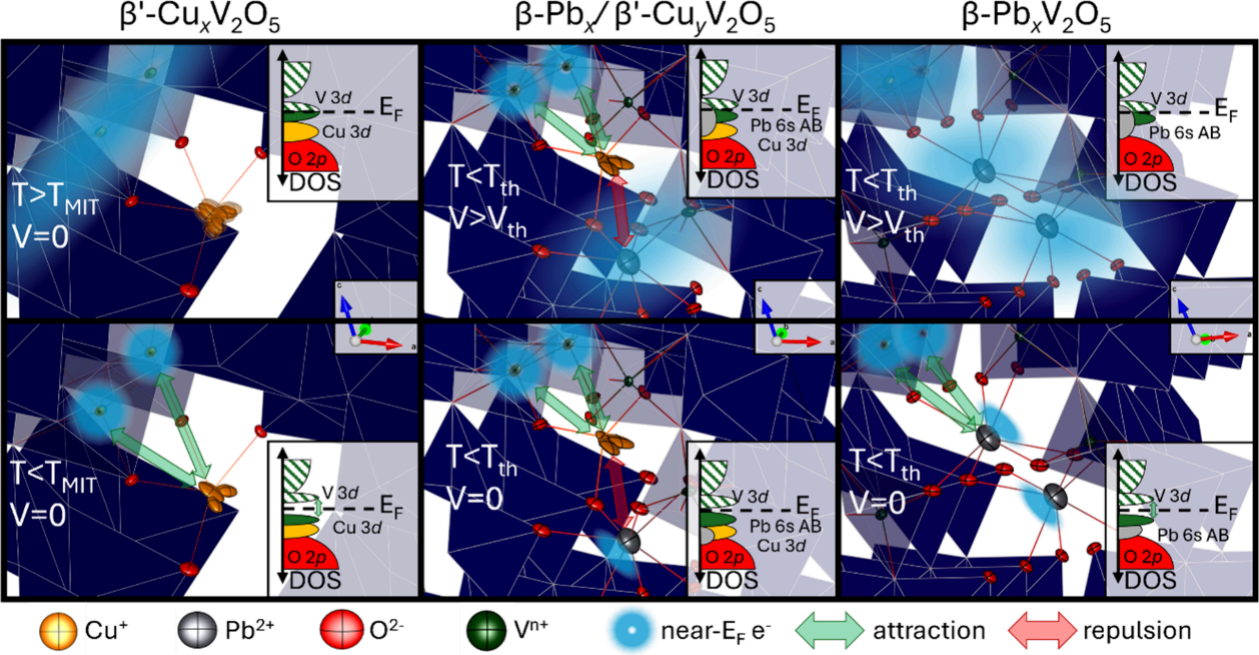
Mechanism underpinning conductance instabilities in end-member M_*x*_V_2_O_5_ (M = Pb, Cu) and coinserted M_*x*_M′_*y*_V_2_O_5_. Evolution of conductance switching as a function of temperature and voltage in (left) β′-Cu_0.33_V_2_O_5_, (center) β-Pb_*x*_/β′-Cu_*y*_V_2_O_5_ and (right) β-Pb_0.2_V_2_O_5_. Concentrated blue clouds indicate localized electrons, whereas diffuse clouds indicate delocalized electron density.

Although the voltage-driven nonlinear dynamical conductivity transition in Pb_*x*_V_2_O_5_ is less well-studied, the stereochemical activity of its Pb 6s^2^ electron lone pairs is a consistent observation.^45,46^ The divalent charge of the Pb^2+^-ion has a stronger stabilizing effect on adjacent small polarons than the single charge on the Cu ion, as indicated by Figure S8 and by the larger loss energy and increased intensity of the d–d transition feature in Figure 7F. The localization of both V 3d electrons on the V_2_O_5_ framework and Pb 6s electrons in lone-pair states is represented by concentrated electron clouds in the bottom-right panel of Figure 8. The valence band HAXPES spectra and COHP calculations presented in Figure 7G,H,J indicate that Pb 6s–O 2p hybridized lone pair states lie immediately below the Fermi level, so that their spatial distribution is sensitive to an applied electrical field. Application of sufficient voltage may trigger the insulator-to-metal transition by destabilizing small polarons or charge ordering, or by delocalizing Pb 6s electrons to create new charge carriers (Figure 8, top-right panel).

Site-selective positioning of Pb-ions in seven-coordinated β sites broadens and eventually suppresses the temperature-induced conductivity transition in β′-Cu_*x*_V_2_O_5_ (contrast Figure 3A to 3B). The corresponding decrease in the magnitude of the structural transition at the critical temperature (Figures 5G–I and S15) indicates that Pb reduces the effect of Cu-ion motion on electron transport by (i) interrupting correlated Cu-ion motion by direct electrostatic interactions (Figure 8, bottom middle panel) with adjacent stereochemically expressed Pb^2+^ ion or via structural distortion of the V_2_O_5_ tunnel that reduces Cu-ion shuttling (Figure S16) and (ii) direct Coulombic stabilization of a localized electron on adjacent V sites. As shown in Figure S9, Pb incorporation gradually imparts a voltage-driven transition akin to that of the β-Pb_*x*_V_2_O_5_ end-member. Cu- and Pb-ions thus retain their distinct charge localization/delocalization mechanisms, albeit with some antagonistic interactions. Pb coinsertion constitutes an atom-precise means of introducing weak disorder in β′-Cu_*x*_V_2_O_5_, enabling systematic broadening of structural and electronic first-order phase transitions.

## Conclusions

Single crystals of coinserted quaternary β-Pb_*x*_/β′-Cu_*y*_V_2_O_5_ have been grown by melt growth. Pb- and Cu-ions fractionate across distinctive seven-coordinated β- and five-coordinated β′-sites, respectively, within a tunnel-structured framework. The coinserted compounds show highly nonlinear modulation of conductance as a function of temperature and applied voltage, from a ferromagnetic low conductance state to a paramagnetic higher conductance state, which enables the fabrication of single crystal neuromorphic oscillators. High-resolution structure solutions from single-crystal X-ray diffraction and measurements of local structure using XANES and EXAFS probes provide insights into the mechanisms underpinning conductance nonlinearities. The insertion of Pb-ions props open the 1D tunnels and modifies Cu-ion interactions with the V_2_O_5_ framework, thereby suppressing charge-ordering-mediated first-order structural and electronic phase transitions. Specifically, the 6s^2^ lone pairs on the Pb^2+^ ion are stereochemically active and their expression induces a significant distortion of the local coordination environment, which leads to distinctive off-centering and reduction in the local symmetry of the Pb-ions. The lone pair distortion further enforces charge localization along the 1D V_2_O_5_ tunnels and modulates oxide-mediated superexchange. As such, the stereochemical expression of Pb 6s^2^ lone pairs provides an atom-precise means of introducing weak disorder in a Mott material displaying a first-order charge order/disorder phase transition. The resulting broadened structural and electronic phase transitions enable the construction of versatile and durable neuromorphic oscillators with small-cycle oscillations across a broad range of circuit capacitance values (mitigating the fatigue and catastrophic failure resultant from large cycle oscillations for active elements with sharp first-order transitions). The results pave the way to generalizable site-selective modification strategies to precisely modulate conductance nonlinearities and enable the design of highly energy efficient and compact neuromorphic computing primitives that can be leveraged across a substantial design space of circuit elements. Future work will explore the effect of coinsertion of other cations from across the periodic table, using site-selective modification strategies developed for this polymorph^36^ to systematically correlate and modulate neuronal information processing through atomistic modifications. Coupling to external fields other than heat (such as strain, photoexcitation, or ion injection) further holds promise for faster or more efficient conductivity switching via novel mechanisms, with the ultimate aim of ultralow-energy brain-inspired computing. Accessing limits of entropy minimization and energy dissipation will require both site selective modification and selective field coupling such as THz excitation of specific phonon modes to achieve optical spiking or electrochemical ion insertion from solid or liquid electrolytes to achieve synaptic emulation.

## Experimental Section

### Synthesis of β-Pb_*x*_/β′-Cu_*y*_V_2_O_5_ Powders and Single Crystals

The coinserted M_*x*_M′_*y*_V_2_O_5_ compounds were synthesized by the solid-state reaction between Cu metal, lead­(II) oxalate, and α-V_2_O_5_ along a range of Pb and Cu stoichiometries *x* and *y* as per:

$$\alpha -V_{2}O_{5}(s)+x{\mathrm{PbC}}_{2}O_{4}(s)+y\mathrm{Cu}(s)\to \beta -{\mathrm{Pb}}_{x}/\beta^{\prime }-{\mathrm{Cu}}_{y}V_{2}O_{5}(s)+2x{\mathrm{CO}}_{2}(g)$$ 1

Briefly, powder samples of β-Pb_*x*_/β′-Cu_*y*_V_2_O_5_ were prepared by solid-state synthesis by mixing stoichiometric amounts of elemental Cu powder (Sigma-Aldrich, 99% purity), α-V_2_O_5_ (Sigma-Aldrich, 99% purity), and lead­(II) oxalate powder (Strem, 99.9% purity). The mixture was ball-milled in a Spex Certiprep ball-mill for 30 min using yttrium-stabilized zirconia (YSZ) balls as the milling media. The resulting mixture was loaded into an Al_2_O_3_ combustion boat, and placed in a tube furnace (Thermo Scientific, Lindberg/Blue M; model number TF55030A-1 with UT150 controller) at ambient temperature under flowing argon. The furnace was heated to 120 °C for 3 h to remove water, then heated to 530 °C for 18 h, and finally cooled to room temperature. The resulting dark blue-gray powder was ground in a mortar and pestle, loaded into a combustion boat and heated under argon multiple times with the same thermal processing sequence to ensure compositional homogeneity. These stoichiometries minimized the presence of unreacted precursors.

Single-crystals of β-Pb_*x*_/β′-Cu_*y*_V_2_O_5_ were obtained by sealing the prepared powder samples under vacuum in a fused amorphous silica ampule, melting at 750 °C in a programmable furnace (Thermo Scientific Lindberg/Blue M; model number TF55030A-1 with UT150 controller), and cooling through the melting point at a rate of 1 °C/h to 550 °C, followed by cooling to ambient temperature under furnace momentum. The resulting black rod and needle-shaped crystals were stored under air.

### Structure Elucidation

Single-crystal diffraction data were collected on a Bruker Quest X-ray diffractometer utilizing the APEX3 software suite; X-ray radiation was generated from a Mo-Iμs X-ray tube (*K*_α_ = 0.71073 Å). All crystals were placed in a cold N_2_ stream maintained at 110 K. Following unit cell determination, extended data collection was performed using omega and phi scans. Data reduction, integration of frames, merging, and scaling were performed with the program APEX3, and absorption correction was performed utilizing the program SADABS.^79,80^ Structures were solved using intrinsic phasing. Least-squares refinement for all structures was carried out on *F* ^2^. Structural refinement, the calculation of derived results, and generation of electron-density maps were performed using the SHELXTL package of computer programs, ShelXle, and Olex2.^81–83^ A Crystallographic Information File for β-Pb_*x*_/β′-Cu_*y*_V_2_O_5_ has been deposited in the Cambridge Structural Database and is available for access with deposition numbers 2373863 (β-Pb_0.11_/β′-Cu_0.41_V_2_O_5_), 2321463 (β-Pb_0.08_/β′-Cu_0.33_V_2_O_5_), and 2321464 (β-Pb_0.01_/β′-Cu_0.47_V_2_O_5_), and 2373861 (β′-Cu_0.53_V_2_O_5_), 2373862 (β′-Cu_0.65_V_2_O_5_).

### Synchrotron XRD and X-ray Pair Distribution Function (X-PDF)

Temperature-dependent synchrotron X-ray pair distribution function and X-ray diffraction measurements were performed at beamline 28-ID-1 at the National Synchrotron Light Source II, with X-rays generated by a damping wiggler source and wavelength selected by a bent Laue monochromator. An X-ray wavelength of 0.166553 Å was determined by refinement against a LaB_6_ standard. β-Pb_*x*_/β′-Cu_*y*_V_2_O_5_ powders were loaded into 1 mm inner diameter polyimide tubes. XRD and PDF measurements were collected back-to-back at each temperature by translating the detector along the beam path to capture a *Q* range of (0.5191–8.7275 Å^–1^) for XRD and (0.0–24.0 Å^–1^) for PDF data. Each specimen was cooled to 90 K and measured at 5 K increments from 90 to 400 K after 5 min of thermal equilibration, with temperature control achieved using a liquid nitrogen cryostream. Sequential refinements of X-ray diffraction patterns were performed using GSAS-II. The atomic positions, profile terms, and lattice parameters are tabulated in the Supporting Information. All crystal structure renditions depicted in this article were prepared using the Vesta III software suite (JP-Minerals).^84^ The pair distribution functions (PDF), *G*(*r*), were obtained by the transformation of the normalized total scattering function, *S*(*Q*), with a *Q*_max_ = 24.0 Å^–1^. Corrections to obtain the *S*(*Q*) and subsequent Fourier transform with a *Q*_max_ of 24.0 Å^–1^ to obtain the *G*(*r*) was performed using the program PDFgetX3.^85^ The processing parameters used for the data conversion are: *Q*_min_ = 0.0 Å^–1^, *Q*_max_ = 24.0 Å ^–1^, *r*_min_ = 1.0 Å and *r*_max_ = 20 Å. Simulation of the experimental PDF data was done using PDFgui software.^86^ All the data sets from 100 to 400 K were initially modeled using a monoclinic *C*2/*m* structure. The refinement parameters were the scale, linear atomic correlation factor, lattice parameter, and the thermal displacement values.

### Electron Microscopy

Scanning electron microscopy (SEM) images were obtained using a JEOL JSM-7500F FE-SEM equipped with an Oxford energy-dispersive X-ray spectrometer (EDS) for elemental characterization. SEM images were collected at an accelerating voltage of 5 kV; EDS spectra were collected at an accelerating voltage of 20 kV. Low-magnification transmission electron microscopy (TEM) images were collected using a JEOL JEM-2010 electron microscope at an operating voltage of 200 kV. Prior to imaging, powder materials were affixed to conductive carbon tape (SEM) or dispersed in 2-propanol and drop-cast onto a copper grid (TEM).

### HAXPES Measurements

Hard X-ray photoemission spectroscopy (HAXPES) measurements were performed at the National Institute of Standards and Technology (NIST) beamline SST-2 of the National Synchrotron Light Source II of Brookhaven National Laboratory. Measurements were performed at approximately 2 keV photon energy with a pass energy of 200 eV and a step size of 0.85 eV with the analyzer axis oriented parallel with the photoelectron polarization vector. HAXPES data at 5 keV was collected with a 500 eV energy filter. The higher excitation energy of HAXPES circumvents deleterious charging issues that are ubiquitous in ultraviolet and soft X-ray photoelectron spectroscopy.^87^ Photon energy selection was accomplished using a double Si(111) crystal monochromator. No evidence of charging was observed during our measurements. The beam energy was aligned to the Fermi level of a metallic silver foil before measurements.

### Magnetic Measurements

Magnetic measurements of β'-Cu_x_V_2_O_5_ and β-Pb_x_/β'-Cu_y_V_2_O_5_ powders were carried out on a Quantum Design Magnetic Property Measurement System using the Quantum Design superconducting quantum interference device (SQUID) magnetometer option. Both zero-field cooled (ZFC) and field-cooled (FC) measurements have been performed in the temperature range of 2–400 K with an applied field up to 0.1 T. Field-dependent magnetization measurements were performed at 2 K and above room temperature under an applied magnetic field ranging from −7 to +7 T.

### Electrical Transport and Oscillator Measurements

Thin, single-crystals of β-Pb_x_/β'-Cu_y_V_2_O_5_ were mounted flat on a glass substrate using GE varnish with two ends of the crystals covered with conducting Ag paste to act as electrodes. DC and/or AC electrical resistance measurements were carried out using a variable temperature inset in a cryogen-free system (Janis/Lakeshore cryogenics) between 50 and 300 K with temperature ramp rates between 1.0 and 2 K/min. Nonlinear current–voltage measurements were carried out at fixed temperatures with a ramp rate of 0.1 V/s and by capping the current to protect the sample from overheating. A Keithley 2450 source-measure unit was used to measure current–voltage traces while resistance measurements were done using either the Keithley 2450 SMU or with a Signal Recovery 7265 DSP lock-in amplifier. Oscillations were recorded using a GW Instek GDS-2062 oscilloscope.

### Electrothermal Oscillator Simulations

Capacitance-dependent electro-thermal oscillations were simulated using a physics-based compact model that combines the material's electrical and thermal properties with the nonlinear dynamics described by local activity theory. The model quantitatively connects intrinsic material properties to key performance parameters, including frequency, amplitude of electrical and thermal oscillations, and power. The compact model, previously described in ref 88, uses a coupled system of two first-order differential equations; (1) Kirchhoff voltage law, (2) Joule heating and Newton's law of cooling. Simulations were performed with Runge–Kutta numerical integrator. The model is based on the following assumptions; (i) that quantities are uniform throughout the device; (ii) negligible series resistor; and (iii) simplified thermal transport. The active material's temperature-dependent electrical conductivity was based on the values shown in Figure 3A. Temperature-dependent thermal conductivity and specific heat capacity were obtained from Wang et al.^89^ and Drake et al.,^90^ respectively, with each data set fitted to physics-based models, providing analytical representations of their behavior.

### Computational Methods

Electronic structure calculations were performed using density functional theory as implemented in the Vienna ab initio simulation package (VASP).^91–93^ Initial atomic positions for β-Pb_*x*_/β′-Cu_*y*_V_2_O_5_ were obtained from structure solutions derived from high-resolution single-crystal X-ray diffraction data. The projected augmented wave (PAW) formalism was used to model electron–ion interactions.^94^ A kinetic energy cutoff of 520 eV was used for plane-wave basis restriction. Electronic exchange and correlation effects were included using the generalized gradient approximation based on the Perdew–Burke–Ernzerhof functional (GGA-PBE).^95^ A Hubbard correction of *U* = 3.25 eV was used to account for strong electron correlation in the V 3d electrons as benchmarked in a previous study.^96^ A Monkhorst–Pack reciprocal grid of 2 × 4 × 4 points was used for the relaxation of 1 × 2 × 1 supercell structures. The structures were relaxed when each Cartesian force component was less the 0.01 eV/Å unless otherwise noted. Electron localization function plots were produced by the VASP output in Vesta.^97^ COHP analyses were performed using the software package Local Orbital Suite Toward Electronic-Structure Reconstruction (LOBSTER).^97,98^ LOBSTER-recommended basis functions were used for the projection calculations accounting for 2s and 2p orbitals of oxygen; 3d and 4s orbitals of vanadium and copper; and 5d, 6s, and 6p orbitals of Pb. The absolute charge spilling is <3.56% in all cases.

### Extended X-ray Absorption Fine Structure (EXAFS) Spectroscopy

V K-edge, Cu K-edge, and Pb L_III_-edge X-ray absorption spectroscopy (XAS) scans were acquired at beamline 7-BM at National Synchrotron Light Source II of Brookhaven National Laboratory. Samples were prepared by uniformly spreading powder onto a piece of polyimide (Kapton) tape. The polyimide tape was then loaded onto a sample holder, and 20 scans were performed at 30 s per scan and subsequently averaged to improve the signal-to-noise ratio. Before sample acquisition, the beamline was calibrated by placing metallic vanadium, copper, and lead foils, and measuring the edge position. Spectra were collected in both fluorescent and transmittance modes. The Athena program from the IFEFFIT package was used for data sanitization. Data in the *k* range of 2.5–11.0 Å^–1^ was Fourier transformed to obtain R-space data. The *R*-space data was used to perform shell fitting. Fitting was performed for the major shells between *R* space = 1.1–4.0 Å. Multishell least-squares parameter fitting of V K-edge, Cu K-edge and Pb L_III_-edge EXAFS data was performed using the ARTEMIS module of the IFEFFIT software package.^99^ The photoelectron mean free path, scattering amplitude, and phase functions were calculated using the FEFF6 program. Atomic coordinates and lattice parameters obtained from crystallography data were used to build initial models for EXAFS fitting.

### X-ray Absorption Near-Edge Structure (XANES) Spectroscopy

V L-, and O K-XANES measurements were carried out at the National Synchrotron Light Source II of Brookhaven National Laboratory beamline SST-1 operated by the National Institute of Standards and Technology. Measurements were performed in partial electron yield (PEY) mode with a nominal resolution of 0.1 eV. The PEY signal was normalized to the incident beam intensity of a clean gold grid to eliminate the effects of any incident beam fluctuations and optics absorption features.

### Resonant Inelastic X-ray Scattering

RIXS spectra were collected at the Advanced Light Source beamline 8.0.1.1's high-efficiency iRIXS endstation^100^ using linearly polarized (perpendicular to the scattering plane) radiation supplied by an undulator and spherical grating monochromator. Excitation energy was calibrated to the V L_3_ highest-intensity V L_3_ feature of an α-V_2_O_5_ standard, and emission energies were calibrated by applying a linear fit to the elastic (zero energy-loss) feature.

## Supplementary Material



## Data Availability

The data that support the findings of this study are available from the corresponding author upon reasonable request.

## Notes

Additional experimental results, including SEM images and EDS spatial elemental maps; crystal data and structure refinement tables; transport measurements; oscillator characterization; diffraction contour color maps; additional magnetometry measurements; EXAFS fitting results; density of states calculation results; and additional resonant inelastic X-ray scattering results (PDF)
