# Supplementary material for: An Atom-Precise Approach to Damp First-Order Phase Transitions and Its Implications for Neuromorphic Signal Processing
Source: J Am Chem Soc. 2026 May 13;148(20):20677–93. doi: 10.1021/jacs.6c02370 (PMC13220263; doi:10.1021/jacs.6c02370)
Supplement: Supplementary file 1 [file ja6c02370_si_001.pdf]

**Supporting Information**

# An Atom-Precise Approach to Damp First-Order Phase Transitions and its Implications for Neuromorphic Signal Processing

George Agbeworvi,[1§] Nitin Kumar,[2§] John D. Ponis,[1§] Shruti Hariyani,[1,3,4§] Nicholas Jerla,[2] Fatme Jardali,[5] Jialu Li,[6] Wasif Zaheer,[1] Joseph V. Handy,[1] Jaime R. Ayala,[1] Cherno Jaye,[7] Conan Weiland,[7] Daniel A. Fischer,[7] Patrick J. Shamberger,[5] Jinghua Guo,[6] R. Stanley Williams,[8] G. Sambandamurthy,[2,]* and Sarbajit Banerjee[3,4,5]*

1. Department of Chemistry, Texas A&M University, College Station, TX, 77843, USA;

2. Department of Physics, University at Buffalo, The State University of New York, Buffalo, New York 14260-1500, USA;

3. Laboratory for Inorganic Chemistry, Department of Chemistry and Applied Biosciences, ETH Zurich, Vladimir-Prelog-Weg 2, CH-8093 Zürich, Switzerland

4. Laboratory for Battery Science, PSI Center for Energy and Environmental Sciences, Paul Scherrer Institute, Forschungsstrasse 111, CH-5232 Villigen PSI, Switzerland

5. Department of Material Science and Engineering, Texas A&M University, College Station, TX, 77843, USA;

6. Advanced Light Source, Lawrence Berkeley National Laboratory, Berkeley, CA 94720, USA;

7. Material Measurement Laboratory, National Institute of Standards and Technology, Gaithersburg, MD, 20899, USA;

8. Department of Electrical Engineering, Texas A&M University, College Station, TX 77843, USA

[§]these authors contributed equally; * Corresponding author

Corresponding Author's Email Address: sbanerje@ethz.ch, sg82@buffalo.edu

Lead Contact's email address: sbanerje@ethz.ch

*KEYWORDS. Stereochemically active electron lone pairs, Revised lone pair model, Magnetism, Metal insulator transition, Single crystals, Intercalation; Hard X-ray photoemission, Bonding.*

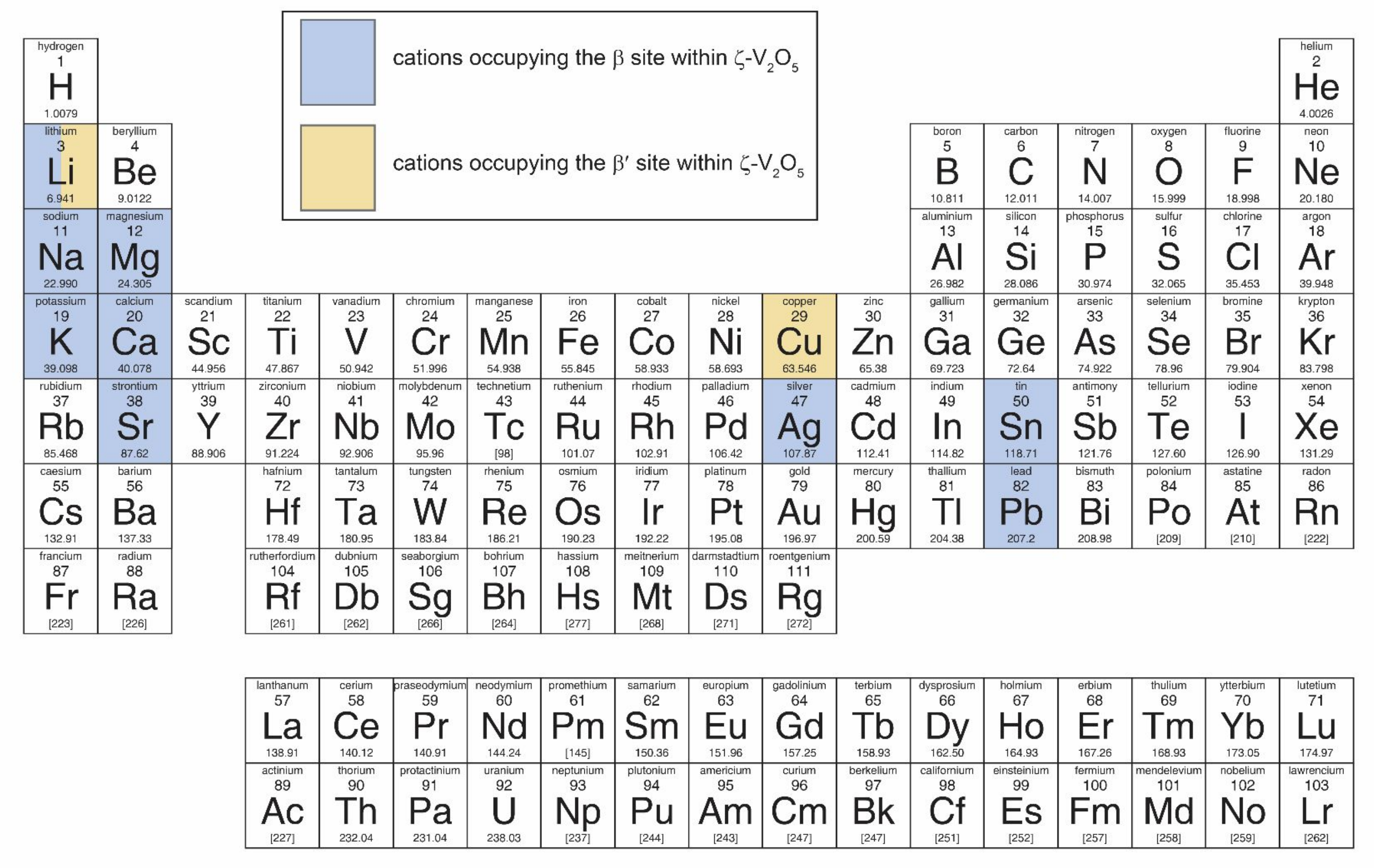


***Figure S1. Inserted Cations within ζ-$V_2O_5$ Framework***. *A* periodic table highlighting the identity of ions that are known to be inserted into the ζ-$V_2O_5$ polymorph. Blue and yellow shading denote cations that occupy seven-coordinated β and five—coordinated β' sites, respectively. This site preference is largely governed by ionic size, charge density, and coordination environment; larger cations favor the higher-coordinate β site and smaller cations preferentially occupy the lower-coordinate β' site. Consistent with these trends, cations such as $Pb^{2+}$ (1.23 Å) and $Ag^{+}$(1.15 Å) preferentially occupy the seven-coordinated β site, whereas smaller ions such as $Cu^{+}$ (0.67 Å) favor the five-coordinated β' site.[1] The distribution shown here summarizes known insertion behavior and provides a framework for understanding site-selective incorporation in the present system.

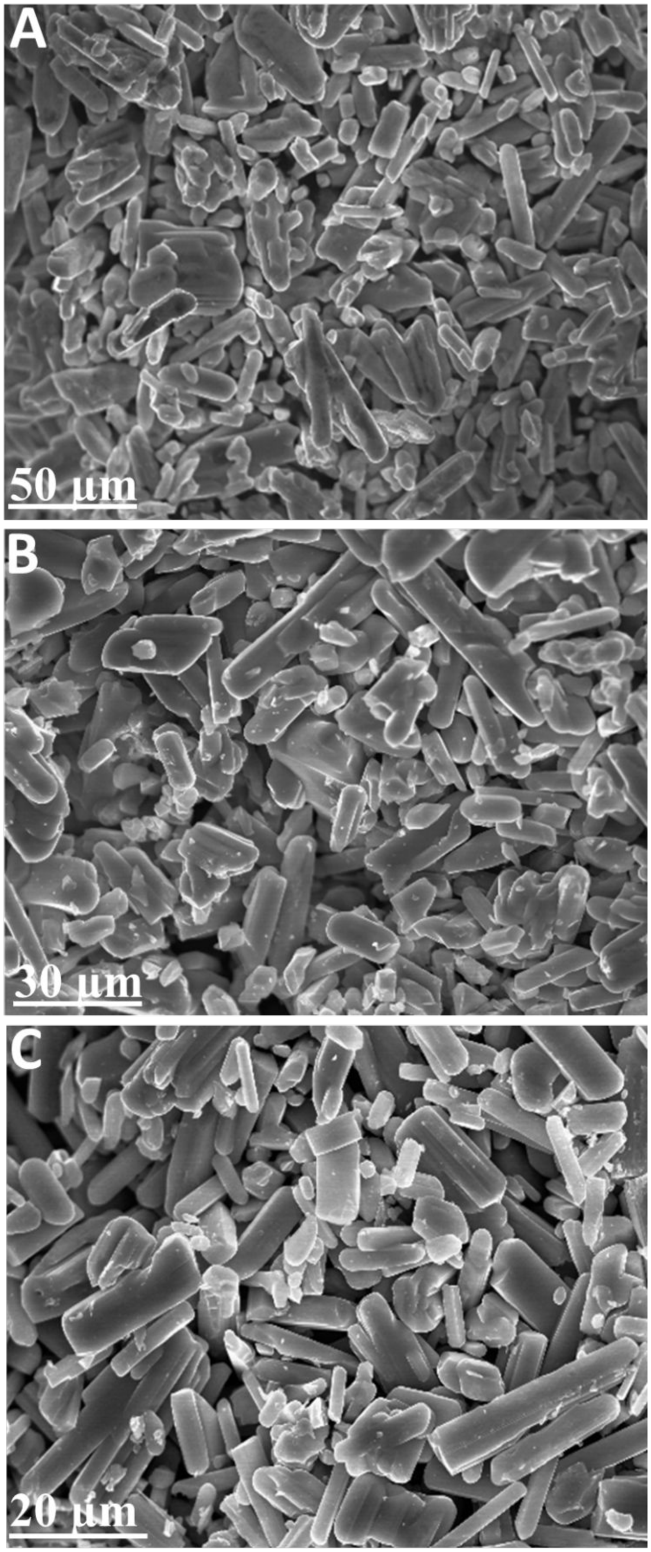


***Figure S2. Crystal morphology of β-Pb$_x$/β'-Cu$_y$V$_2$O$_5$ Powders**.* SEM images acquired for **(A)** β-Pb$_{0.01}$/β'-Cu$_{0.47}$V$_2$O$_5$; **( B)** β-Pb$_{0.08}$/β'-Cu$_{0.33}$V$_2$O$_5$; and **(C)** β-Pb$_{0.11}$/β'-Cu$_{0.41}$V$_2$O$_5$.

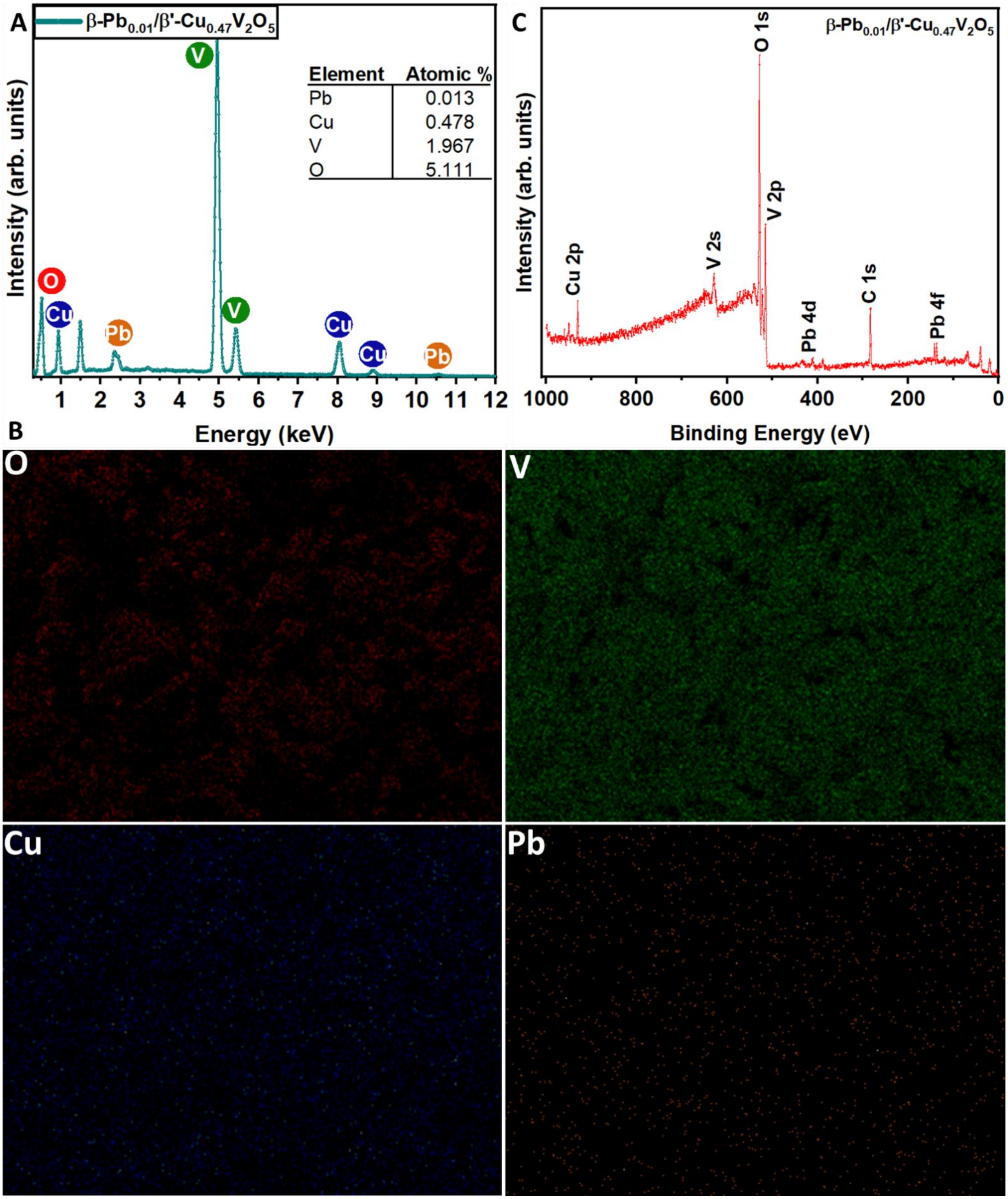


***Figure S3. EDS analysis of β-$Pb_{0.01}$/β'-$Cu_{0.47}V_2O_5$.*** Energy dispersive X-ray spectroscopy (EDS) (**A, B**) spatial elemental maps of β-$Pb_{0.01}$/β'-$Cu_{0.47}V_2O_5$. The corresponding image of β-$Pb_{0.01}$/β'-$Cu_{0.47}V_2O_5$ from which the maps are derived is presented in **Figure S2A**. The inset table (top right) shows the elemental composition of β-$Pb_{0.01}$/β'-$Cu_{0.47}V_2O_5$ as measured by EDS, which is consistent with the nominal Pb stoichiometry. (C) 2 keV HAXPES survey spectra of β-$Pb_{0.01}$/β'-$Cu_{0.47}V_2O_5$.

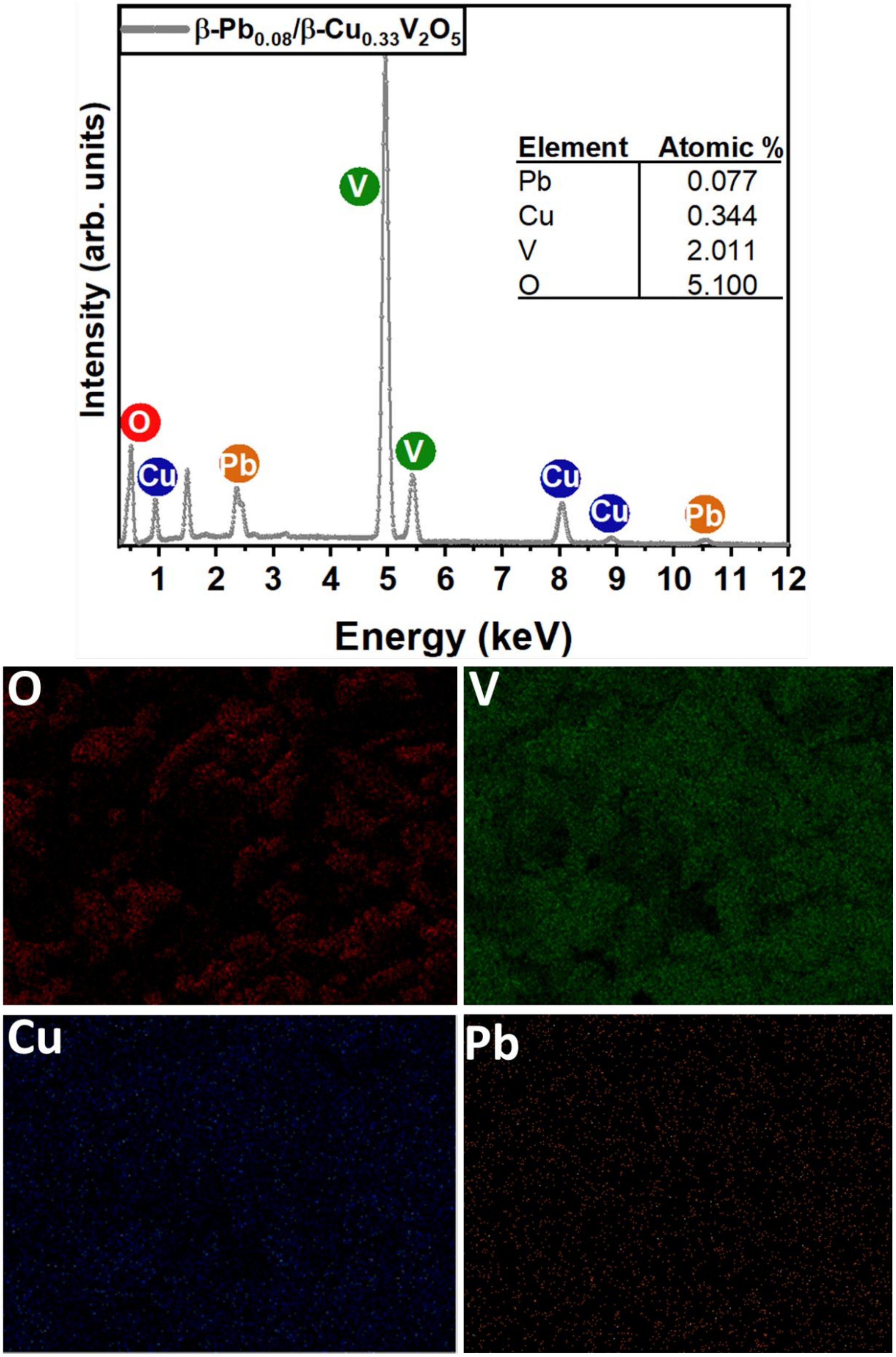


***Figure S4. EDS analysis of β-$Pb_{0.08}$/β'-$Cu_{0.33}V_2O_5$.*** Energy dispersive X-ray spectroscopy (EDS) spatial elemental maps of β-$Pb_{0.08}$/β'-$Cu_{0.33}V_2O_5$. The corresponding SEM image of β-$Pb_{0.08}$/β'-$Cu_{0.33}V_2O_5$ from which the maps are derived is presented in **Figure S2B**. The inset table (top right) shows the elemental composition of β-$Pb_{0.08}$/β'-$Cu_{0.33}V_2O_5$ as measured by EDS, which is consistent with the nominal Pb stoichiometry.

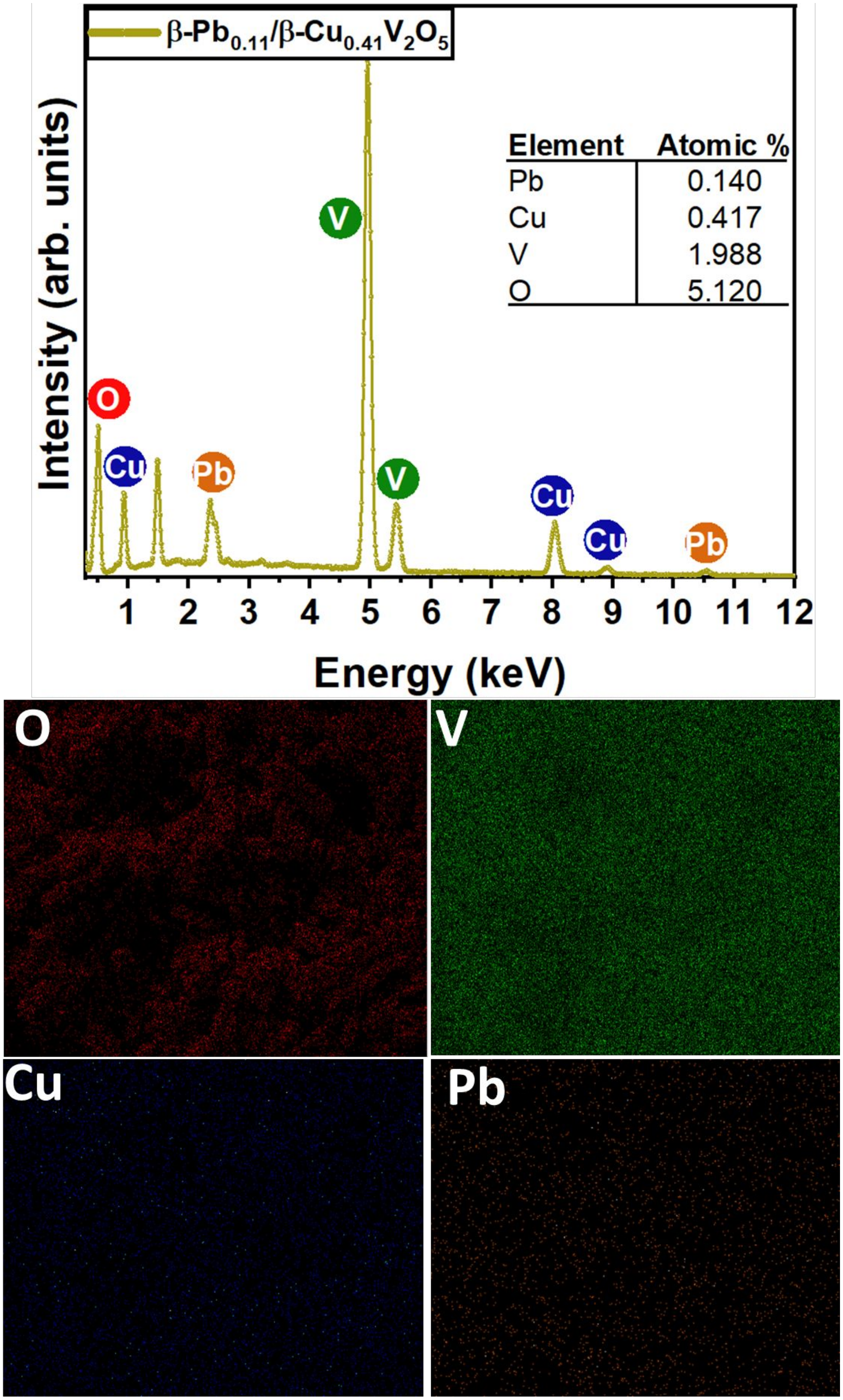


***Figure S5. EDS analysis of $\beta$-Pb$_{0.11}$/$\beta$'-Cu$_{41}$V$_2$O$_5$.*** Energy dispersive X-ray spectroscopy (EDS) spatial elemental maps of the elemental $\beta$-Pb$_{0.11}$/$\beta$'-Cu$_{0.41}$V$_2$O$_5$. The corresponding SEM image of $\beta$-Pb$_{0.11}$/$\beta$'-Cu$_{0.41}$V$_2$O$_5$ from which the maps are derived is presented in **Figure S2C**. The inset table (top right) shows the elemental composition of $\beta$-Pb$_{0.11}$/$\beta$'-Cu$_{0.41}$V$_2$O$_5$ as measured by EDS, which is consistent with the nominal Pb stoichiometry.

***Table S1. Crystal data and structure refinement for β'-$Cu_{0.45}V_2O_5$:bCuV2O5_0354_110K_0m_a.***

| | | |
|---|---|---|
| **Identification code** | 0354bCuV2O5 | |
| **Empirical formula** | Cu0.45O5V2 | |
| **Formula weight** | 210.41 | |
| **Temperature** | 110.00 K | |
| **Wavelength** | 0.71073 Å | |
| **Crystal system** | Monoclinic | |
| **Space group** | *C 2/m* | |
| **Unit cell dimensions** | *a* = 15.1981(7) Å | α = 90°. |
| | *b* = 3.6257(2) Å | β = 106.567(2)° |
| | *c* = 10.0701(5) Å | γ = 90°. |
| **Volume** | 531.86(5) $Å^3$ | |
| ***Z*** | 6 | |
| **Density (calculated)** | 3.941 $mg/m^3$ | |
| **Absorption coefficient** | 7.771 $mm^{-1}$ | |
| **F(000)** | 594 | |
| **Crystal size** | 0.050 × 0.050 × 0.010 $mm^3$ | |
| **Theta range for data collection** | 2.797 to 32.590° | |
| **Index ranges** | -22<=h<=22, -5<=k<=5, -13<=l<=15 | |
| **Reflections collected** | 1108 | |
| **Independent reflections** | 1108 [R(int) = 0.0505] | |
| **Completeness to theta = 25.242°** | 99.80% | |
| **Absorption correction** | multi-scan | |
| **Max. and min. transmission** | 0.3555 and 0.1742 | |
| **Refinement method** | Full-matrix least-squares on $F^2$ | |
| **Data / restraints / parameters** | 1108 / 0 / 90 | |
| **Goodness-of-fit on $F^2$** | 1.146 | |
| **Final R indices [I>2sigma(I)]** | R1 = 0.0261, wR2 = 0.0406 | |
| **R indices (all data)** | R1 = 0.0195, wR2 = 0.0393 | |
| **Largest diff. peak and hole** | 0.557 and -0.400 | |

***Table S2. Atomic coordinates, occupancies, and equivalent isotropic displacement parameters ($Å^2$) for β'-$Cu_{0.45}V_2O_5$: bCuV2O5_0354_110K_0m_a. U(eq) is defined as one third of the trace of the orthogonalized $U^{ij}$ tensor.***

| Atom | *x* | *y* | *z* | Occupancy | Uiso |
|---|---|---|---|---|---|
| Cu(1) | 0.4570(7) | 1.0000 | 0.6567(12) | 0.170 | 0.009(13) |
| Cu(2) | 0.4701(5) | 0.936(3) | 0.6412(6) | 0.238 | 0.024(12) |
| Cu(3) | 0.5026(11) | 0.500000 | 0.573(2) | 0.025 | 0.039(6) |
| V(1) | 0.71382(3) | 0.0000 | 0.59437(4) | 1 | 0.005(9) |
| V(2) | 0.33349(2) | 0.0000 | 0.09210(3) | 1 | 0.003(9) |
| V(3) | 0.38624(3) | 0.5000 | -0.12045(4) | 1 | 0.005(9) |

| | | | | | |
|---|---|---|---|---|---|
| O(1) | 0.74472(11) | 0.5000 | 0.57576(16) | 1 | 0.008(3) |
| O(2) | 0.60316(12) | 0.0000 | 0.54067(17) | 1 | 0.013(3) |
| O(3) | 0.73989(10) | 0.0000 | 0.77797(15) | 1 | 0.004(3) |
| O(4) | 0.43258(10) | 0.0000 | 0.20449(16) | 1 | 0.007(3) |
| O(5) | 0.31205(10) | 0.5000 | 0.04752(15) | 1 | 0.005(3) |
| O(6) | 0.5000 | 0.5000 | 0.0000 | 1 | 0.006(4) |
| O(7) | 0.36751(10) | 0.0000 | -0.08390(15) | 1 | 0.005(3) |
| O(8) | 0.40189(11) | 0.5000 | -0.27426(16) | 1 | 0.009(3) |

***Table S3. Anisotropic displacement parameters ($Å^2 x 10^3$) for β'-$Cu_{0.45}V_2O_5$: bCuV2O5_0354_110K_0m_a. The anisotropic displacement factor exponent takes the form: $-2\pi^2[ h^2 a^{*2}U^{11} + ... + 2 h k a^* b^* U^{12}]$***

| Atom | U11 | U22 | U33 | U23 | U13 | U12 |
|---|---|---|---|---|---|---|
| Cu(1) | 6(18) | 13(3) | 5(17) | 0 | -1(11) | 0 |
| Cu(2) | 18(16) | 37(2) | 9(10) | 7(12) | -9(9) | -13(14) |
| Cu(3) | 20(8) | 27(10) | 64(12) | 0 | 2(8) | 0 |
| V(1) | 10(18) | 3(16) | 3(15) | 0 | 2(8) | 0 |
| V(2) | 4(16) | 2(16) | 4(15) | 0 | 1(12) | 0 |
| V(3) | 9(17) | 2(16) | 6(15) | 0 | 5(13) | 0 |
| O(1) | 18(8) | 3(7) | 5(6) | 0 | 6(6) | 0 |
| O(2) | 11(7) | 18(9) | 7(7) | 0 | -1(5) | 0 |
| O(3) | 6(7) | 3(7) | 4(6) | 0 | 2(5) | 0 |
| O(4) | 5(7) | 9(7) | 7(6) | 0 | 0(5) | 0 |
| O(5) | 6(6) | 4(7) | 6(6) | 0 | 2(5) | 0 |
| O(6) | 5(9) | 4(10) | 9(9) | 0 | 1(8) | 0 |
| O(7) | 6(7) | 3(7) | 5(6) | 0 | 2(5) | 0 |
| O(8) | 9(7) | 14(8) | 5(6) | 0 | 3(6) | 0 |

***Table S4. Bond lengths [Å] and angles [°] for β'-$Cu_{0.45}V_2O_5$: bCuV2O5_0354_110K_0m_a.***

| Atom pairs | Bond Length [Å] | Atom pairs | Bond Length [Å] |
|---|---|---|---|
| Cu(3)—Cu(3)#1 | 1.46(4) | V(2)—O(7) | 1.9827(15) |
| Cu(3)—Cu(2) | 1.844(17) | V(2)—O(5)#9 | 2.2574(15) |
| Cu(3)—Cu(2)#2 | 1.844(17) | V(2)—V(3) | 3.0804(4) |
| Cu(3)—Cu(1)#3 | 2.192(13) | V(2)—V(3)#3 | 3.0804(4) |
| Cu(3)—Cu(1) | 2.192(13) | V(3)—O(8) | 1.6328(16) |
| Cu(3)—Cu(2)#4 | 2.255(11) | V(3)—O(6) | 1.8069(4) |
| Cu(3)—Cu(2)#3 | 2.255(11) | V(3)—O(7)#10 | 1.8875(4) |
| Cu(3)—Cu(2)#1 | 2.80(2) | V(3)—O(7) | 1.8875(4) |
| Cu(3)—Cu(2)#5 | 2.80(2) | V(3)—O(3)#11 | 2.1670(15) |
| V(1)—O(2) | 1.6131(17) | V(3)—O(5) | 2.2847(15) |
| V(1)—O(3) | 1.7770(15) | O(2)—Cu(2)#12 | 1.866(4) |

| | | | |
|---|---|---|---|
| V(1)—O(1) | 1.8953(5) | O(2)—Cu(2)#1 | 1.866(4) |
| V(1)—O(1)#3 | 1.8953(5) | O(2)—Cu(1)#1 | 1.937(11) |
| V(1)—O(1)#4 | 1.9868(16) | O(4)—Cu(2)#1 | 1.830(4) |
| V(1)—V(1)#7 | 3.0496(6) | O(4)—Cu(2)#12 | 1.830(4) |
| V(1)—V(1)#6 | 3.0496(6) | O(4)—Cu(1)#1 | 1.853(10) |
| V(1)—Cu(1)#1 | 3.064(10) | O(8)—Cu(2)#13 | 2.191(5) |
| V(2)—O(4) | 1.6053(15) | O(8)—Cu(2)#14 | 2.191(5) |
| V(2)—O(5)#3 | 1.8737(4) | O(8)—Cu(1)#13 | 2.191(7) |
| V(2)—O(5) | 1.8737(4) | O(8)—Cu(1)#15 | 2.191(7) |
| V(2)—O(3)#8 | 1.9462(15) | V(1)—V(3) | 3.2897(6) |

| Atoms | Bond Angle [°] | Atoms | Bond Angle [°] |
|---|---|---|---|
| Cu(3)#1—Cu(3)—Cu(2) | 115.7(8) | O(2)—V(1)—O(1)#3 | 103.35(5) |
| Cu(3)#1—Cu(3)—Cu(2)#2 | 115.7(7) | O(3)—V(1)—O(1)#3 | 96.60(5) |
| Cu(3)#1—Cu(3)—Cu(1)#3 | 117.2(8) | O(1)—V(1)—O(1)#3 | 146.07(10) |
| Cu(3)#1—Cu(3)—Cu(1) | 117.2(8) | O(2)—V(1)—O(1)#6 | 105.52(8) |
| Cu(1)#3—Cu(3)—Cu(1) | 111.6(10) | O(3)—V(1)—O(1)#6 | 149.97(7) |
| Cu(3)#1—Cu(3)—Cu(2)#4 | 110.7(6) | O(1)—V(1)—O(1)#6 | 76.48(5) |
| Cu(2)—Cu(3)—Cu(2)#4 | 6.1(3) | O(1)#3—V(1)—O(1)#6 | 76.48(5) |
| Cu(2)#2—Cu(3)—Cu(2)#4 | 124.1(12) | O(2)—V(1)—V(1)#7 | 108.53(5) |
| Cu(1)#3—Cu(3)—Cu(2)#4 | 120.9(11) | O(3)—V(1)—V(1)#7 | 129.45(3) |
| Cu(1)—Cu(3)—Cu(2)#4 | 9.4(3) | O(1)—V(1)—V(1)#7 | 111.31(5) |
| Cu(3)#1—Cu(3)—Cu(2)#3 | 110.7(6) | O(1)#3—V(1)—V(1)#7 | 39.31(4) |
| Cu(1)#3—Cu(3)—Cu(2)#3 | 9.4(3) | O(1)#6—V(1)—V(1)#7 | 37.177(12) |
| Cu(1)—Cu(3)—Cu(2)#3 | 120.9(11) | O(2)—V(1)—V(1)#6 | 108.53(5) |
| Cu(3)#1—Cu(3)—Cu(2)#1 | 36.4(4) | O(3)—V(1)—V(1)#6 | 129.45(4) |
| Cu(1)#3—Cu(3)—Cu(2)#1 | 87.8(3) | O(1)—V(1)—V(1)#6 | 39.31(4) |
| Cu(1)—Cu(3)—Cu(2)#1 | 153.5(9) | O(1)#3—V(1)—V(1)#6 | 111.31(5) |
| Cu(3)#1—Cu(3)—Cu(2)#5 | 36.4(4) | O(1)#6—V(1)—V(1)#6 | 37.177(12) |
| Cu(1)#3—Cu(3)—Cu(2)#5 | 153.5(9) | V(1)#7—V(1)—V(1)#6 | 72.947(17) |
| Cu(1)—Cu(3)—Cu(2)#5 | 87.8(3) | O(2)—V(1)—Cu(1)#1 | 33.5(2) |
| O(2)—V(1)—O(3) | 104.51(8) | O(3)—V(1)—Cu(1)#1 | 138.0(2) |
| O(2)—V(1)—O(1) | 103.35(5) | O(1)—V(1)—Cu(1)#1 | 95.39(8) |
| O(3)—V(1)—O(1) | 96.60(5) | O(1)#3—V(1)—Cu(1)#1 | 95.39(8) |
| O(2)—V(1)—O(1)#3 | 103.35(5) | O(1)#6—V(1)—Cu(1)#1 | 72.0(2) |
| O(3)—V(1)—O(1)#3 | 96.60(5) | V(1)#7—V(1)—Cu(1)#1 | 81.77(19) |
| O(1)—V(1)—O(1)#3 | 146.07(10) | V(1)#6—V(1)—Cu(1)#1 | 81.77(19) |
| O(2)—V(1)—O(1)#6 | 105.52(8) | O(4)—V(2)—O(5)#3 | 103.47(5) |
| O(3)—V(1)—O(1)#6 | 149.97(7) | O(4)—V(2)—O(5) | 103.47(5) |
| O(1)—V(1)—O(1)#6 | 76.48(5) | O(5)#3—V(2)—O(5) | 150.72(9) |
| O(7)—V(3)—O(3)#11 | 84.29(5) | O(4)—V(2)—O(3)#8 | 97.37(7) |
| O(8)—V(3)—O(5) | 159.80(7) | O(5)#3—V(2)—O(3)#8 | 93.86(5) |
| O(6)—V(3)—O(5) | 94.75(4) | O(5)—V(2)—O(3)#8 | 93.86(5) |
| O(7)#10—V(3)—O(5) | 73.86(4) | O(4)—V(2)—O(7) | 101.47(7) |
| O(7)—V(3)—O(5) | 73.86(4) | O(5)#3—V(2)—O(7) | 81.82(5) |
| O(3)#11—V(3)—O(5) | 72.10(5) | O(5)—V(2)—O(7) | 81.82(5) |
| O(8)—V(3)—V(2)#10 | 142.783(15) | O(3)#8—V(2)—O(7) | 161.16(6) |
| O(6)—V(3)—V(2)#10 | 85.546(13) | O(4)—V(2)—O(5)#9 | 174.14(7) |
| O(7)#10—V(3)—V(2)#10 | 38.34(4) | O(5)#3—V(2)—O(5)#9 | 77.19(5) |
| O(7)—V(3)—V(2)#10 | 110.25(4) | O(5)—V(2)—O(5)#9 | 77.19(5) |

**Symmetry transformations used to generate equivalent atoms:**

(i) 1-x, 1-y, 1-z; (ii) x, 1-y, z; (iii) x, -1+y, z; (iv) x, 2-y, z;

(v) 1-x, y, 1-z; (vi) 1.5-x, 0.5-y, 1-z; (vii) 1.5-x, -0.5-y, 1-z; (viii) 1-x, -y, 1-z; (ix) 0.5-x, 0.5-y, -z; (x) x, 1+y, z; (xi) -0.5+x, 0.5+y, -1+z; (xii) 1-x, -1+y, 1-z; (xiii) x, y, -1+z; (xiv) x, 1-y, -1+z; (xv) x, -1+y, -1+z; (xvi) 0.5+x, -0.5+y, 1+z; (xvii) 1-x, 1-y, -z; (xviii) x, y, 1+z; (xix) x, 1+y, 1+z.

### *Table S5. Crystal data and structure refinement for β′-$Cu_{0.53}V_2O_5$: bcuv2o5.*

| | | |
|---|---|---|
| **Identification code** | *bcuv2o5* | |
| **Empirical formula** | Cu0.53 O5 V2 | |
| **Formula weight** | 215.34 | |
| **Temperature** | 110.00 K | |
| **Wavelength** | 0.71073 Å | |
| **Crystal system** | Monoclinic | |
| **Space group** | *C 2/m* | |
| **Unit cell dimensions** | *a* = 15.1590(10) Å | α = 90°. |
| | *b* = 3.6294(2) Å | β = 106.066(2)° |
| | *c* = 10.0739(7) Å | γ = 90°. |
| **Volume** | 532.60(6) $Å^3$ | |
| ***Z*** | 6 | |
| **Density (calculated)** | 4.03 mg/$m^3$ | |
| **Absorption coefficient** | 8.223 $mm^{-1}$ | |
| **F(000)** | 608 | |
| **Crystal size** | 0.312 × 0.082 × 0.036 $mm^3$ | |
| **Theta range for data collection** | 2.104 to 40.231° | |
| **Index ranges** | -27<=h<=27, -6<=k<=6, -18<=l<=18 | |
| **Reflections collected** | 1869 | |
| **Independent reflections** | 1869 [R(int) = 0.0609] | |
| **Completeness to theta = 25.242°** | 99.80% | |
| **Absorption correction** | multi-scan | |
| **Max. and min. transmission** | 0.5008 and 0.2435 | |
| **Refinement method** | Full-matrix least-squares on $F^2$ | |
| **Data / restraints / parameters** | 1869 / 0 / 83 | |
| **Goodness-of-fit on $F^2$** | 1.393 | |
| **Final R indices [I>2sigma(I)]** | R1 = 0.0324, wR2 = 0.0515 | |
| **R indices (all data)** | R1 = 0.0275, wR2 = 0.0490 | |
| **Largest diff. peak and hole** | 1.000 and -0.883 e.$Å^{-3}$ | |

***Table S6. Atomic coordinates, occupancies, and equivalent isotropic displacement parameters ($Å^2$) for β′-$Cu_{0.53}V_2O_5$: bcuv2o5. U(eq) is defined as one third of the trace of the orthogonalized $U^{ij}$ tensor.***

| Atom | *x* | *y* | *z* | Occupancy | Uiso |
|---|---|---|---|---|---|
| Cu(1) | 0.4576(5) | 1.0000 | 0.1561(7) | 0.230 | 0.010(8) |
| Cu(2) | 0.4699(4) | 1.056(2) | 0.1428(5) | 0.279 | 0.022(8) |
| V(1) | 0.28664(2) | 1.0000 | -0.09416(3) | 1 | 0.004(6) |
| V(2) | 0.66712(2) | 1.0000 | 0.40938(3) | 1 | 0.003(6) |
| V(3) | 0.61366(2) | 0.5000 | 0.62091(3) | 1 | 0.006(6) |

| | | | | | |
|---|---|---|---|---|---|
| O(1) | 0.25540(10) | 0.5000 | -0.07592(14) | 1 | 0.007(2) |
| O(2) | 0.39818(10) | 1.0000 | -0.04044(15) | 1 | 0.010(2) |
| O(3) | 0.26021(9) | 1.0000 | -0.27742(13) | 1 | 0.005(2) |
| O(4) | 0.56735(9) | 1.0000 | 0.29753(14) | 1 | 0.007(2) |
| O(5) | 0.68825(9) | 0.5000 | 0.45442(14) | 1 | 0.005(2) |
| O(6) | 0.5000 | 0.5000 | 0.5000 | 1 | 0.007(3) |
| O(7) | 0.63237(9) | 1.0000 | 0.58557(13) | 1 | 0.005(2) |
| O(8) | 0.59686(10) | 0.5000 | 0.77533(14) | 1 | 0.008(2) |

***Table S7. Anisotropic displacement parameters ($Å^2$x $10^3$) for β'-$Cu_{0.53}V_2O_5$: bcuv2o5. The anisotropic displacement factor exponent takes the form: $-2\pi^2[ h^2 a^{*2}U^{11} + ... + 2 h k a^* b^* U^{12}]$***

| Atom | U11 | U22 | U33 | U23 | U13 | U12 |
|---|---|---|---|---|---|---|
| Cu(1) | 7(10) | 14(15) | 7(9) | 0 | -3(7) | 0(1) |
| Cu(2) | 16(10) | 3(16) | 9(7) | -6(8) | -9(6) | 10(10) |
| V(1) | 7(12) | 3(11) | 3(11) | 0 | 1(8) | 0 |
| V(2) | 4(11) | 3(11) | 4(10) | 0 | 1(8) | 0 |
| V(3) | 10(13) | 3(11) | 7(11) | 0 | 5(9) | 0 |
| O(1) | 13(6) | 3(5) | 6(5) | 0 | 4(4) | 0 |
| O(2) | 8(5) | 14(6) | 7(5) | 0 | -1(4) | 0 |
| O(3) | 6(5) | 5(5) | 4(4) | 0 | 1(4) | 0 |
| O(4) | 5(5) | 9(5) | 7(5) | 0 | -0(4) | 0 |
| O(5) | 6(5) | 4(5) | 6(5) | 0 | 1(4) | 0 |
| O(6) | 4(7) | 6(7) | 11(8) | 0 | -0(6) | 0 |
| O(7) | 7(5) | 4(5) | 4(5) | 0 | 2(4) | 0 |
| O(8) | 7(5) | 12(6) | 5(5) | 0 | 2(4) | 0 |

***Table S8. Bond lengths [Å] and angles [°] for β'-$Cu_{0.53}V_2O_5$: bcuv2o5.***

| Atom pairs | Bond Length [Å] | Atom pairs | Bond Length [Å] |
|---|---|---|---|
| V(1)—O(2) | 1.6265(15) | V(3)—O(6) | 1.8140(3) |
| V(1)—O(3) | 1.7774(13) | V(3)—O(7)#6 | 1.8858(4) |
| V(1)—O(1) | 1.8972(4) | V(3)—O(7) | 1.8858(4) |
| V(1)—O(1)#1 | 1.8972(4) | V(3)—O(3)#7 | 2.1737(14) |
| V(1)—O(1)#2 | 1.9864(14) | V(3)—O(5) | 2.2675(14) |
| V(1)—V(1)#3 | 3.0483(5) | O(2)—Cu(2)#8 | 1.876(3) |
| V(1)—V(1)#2 | 3.0483(5) | O(2)—Cu(2) | 1.876(3) |
| V(1)—Cu(1) | 3.075(6) | O(2)—Cu(1) | 1.935(7) |
| V(2)—O(4) | 1.6167(14) | O(4)—Cu(2) | 1.838(3) |
| V(2)—O(5)#1 | 1.8765(4) | O(4)—Cu(2)#8 | 1.838(3) |
| V(2)—O(5) | 1.8765(4) | O(4)—Cu(1) | 1.868(6) |
| V(2)—O(3)#4 | 1.9471(13) | O(8)—Cu(1)#9 | 2.183(5) |

| | | | |
|---|---|---|---|
| V(2)—O(7) | 1.9863(13) | O(8)—Cu(1)#10 | 2.183(5) |
| V(2)—O(5)#5 | 2.2416(14) | O(8)—Cu(2)#11 | 2.184(4) |
| V(2)—V(3)#1 | 3.0736(4) | O(8)—Cu(2)#10 | 2.184(4) |
| V(2)—V(3) | 3.0736(4) | Cu(2)—Cu(2)#8 | 0.403(16) |
| V(3)—O(8) | 1.6443(14) | V(1)—V(3) | 3.3089(5) |

| Atoms | Bond Angle [°] | Atoms | Bond Angle [°] |
|---|---|---|---|
| O(2)—V(1)—O(3) | 105.09(7) | O(8)—V(3)—O(5) | 159.92(6) |
| O(2)—V(1)—O(1) | 103.42(5) | O(6)—V(3)—O(5) | 94.51(4) |
| O(3)—V(1)—O(1) | 96.32(4) | O(7)#6—V(3)—O(5) | 74.25(4) |
| O(2)—V(1)—O(1)#1 | 103.42(5) | O(7)—V(3)—O(5) | 74.24(4) |
| O(3)—V(1)—O(1)#1 | 96.32(4) | O(3)#7—V(3)—O(5) | 72.22(5) |
| O(1)—V(1)—O(1)#1 | 146.08(8) | O(8)—V(3)—V(2)#6 | 142.699(13) |
| O(2)—V(1)—O(1)#2 | 105.37(7) | O(6)—V(3)—V(2)#6 | 85.302(12) |
| O(3)—V(1)—O(1)#2 | 149.54(6) | O(7)#6—V(3)—V(2)#6 | 38.61(4) |
| O(1)—V(1)—O(1)#2 | 76.60(4) | O(7)—V(3)—V(2)#6 | 110.79(4) |
| O(1)#1—V(1)—O(1)#2 | 76.60(4) | O(3)#7—V(3)—V(2)#6 | 84.00(3) |
| O(2)—V(1)—V(1)#3 | 108.49(4) | O(5)—V(3)—V(2)#6 | 37.44(1) |
| O(3)—V(1)—V(1)#3 | 129.06(3) | O(8)—V(3)—V(2) | 142.699(13) |
| O(1)—V(1)—V(1)#3 | 111.43(4) | O(6)—V(3)—V(2) | 85.303(12) |
| O(1)#3—V(1)—V(1)#3 | 39.34(4) | O(7)#6—V(3)—V(2) | 110.79(4) |
| O(1)#2—V(1)—V(1)#3 | 37.259(11) | O(7)—V(3)—V(2) | 38.61(4) |
| O(2)—V(1)—V(1)#2 | 108.49(4) | O(3)#7—V(3)—V(2) | 84.00(3) |
| O(3)—V(1)—V(1)#2 | 129.06(3) | O(5)—V(3)—V(2) | 37.439(10) |
| O(1)—V(1)—V(1)#2 | 39.34(4) | V(2)#6—V(3)—V(2) | 72.374(10) |
| O(1)#1—V(1)—V(1)#2 | 111.43(4) | V(1)#6—O(1)—V(1) | 146.08(8) |
| O(1)#2—V(1)—V(1)#2 | 37.259(11) | V(1)#6—O(1)—V(1)#2 | 103.40(4) |
| V(1)#3—V(1)—V(1)#2 | 73.069(14) | V(1)—O(1)—V(1)#2 | 103.40(4) |
| O(2)—V(1)—Cu(1) | 33.35(17) | V(1)—O(2)—Cu(2)#8 | 126.4(3) |
| O(3)—V(1)—Cu(1) | 138.44(17) | V(1)—O(2)—Cu(2) | 126.4(3) |
| O(1)—V(1)—Cu(1) | 95.56(6) | Cu(2)#8—O(2)—Cu(2) | 12.3(5) |
| O(1)#1—V(1)—Cu(1) | 95.56(6) | V(1)—O(2)—Cu(1) | 119.1(3) |
| O(1)#2—V(1)—Cu(1) | 72.02(17) | V(1)—O(3)—V(2)#4 | 134.56(8) |
| V(1)#3—V(1)—Cu(1) | 81.91(13) | V(1)—O(3)—V(3)#12 | 113.36(7) |
| V(1)#2—V(1)—Cu(1) | 81.91(13) | V(2)#4—O(3)—V(3)#12 | 112.08(6) |
| O(4)—V(2)—O(5)#1 | 103.46(4) | V(2)—O(4)—Cu(2) | 165.6(4) |
| O(4)—V(2)—O(5) | 103.46(4) | V(2)—O(4)—Cu(2)#8 | 165.6(3) |
| O(5)#1—V(2)—O(5) | 150.51(8) | Cu(2)—O(4)—Cu(2)#8 | 12.6(5) |
| O(4)—V(2)—O(3)#4 | 96.96(7) | V(2)—O(4)—Cu(1) | 174.9(3) |
| O(5)#1—V(2)—O(3)#4 | 94.25(4) | V(2)—O(5)—V(2)#6 | 150.51(8) |
| O(5)—V(2)—O(3)#4 | 94.25(4) | V(2)—O(5)—V(2)#5 | 102.75(4) |
| O(4)—V(2)—O(7) | 101.22(7) | V(2)#6—O(5)—V(2)#5 | 102.75(4) |
| O(5)#1—V(2)—O(7) | 81.58(4) | V(2)—O(5)—V(3) | 95.29(4) |
| O(5)—V(2)—O(7) | 81.58(4) | V(2)#6—O(5)—V(3) | 95.29(4) |
| O(3)#4—V(2)—O(7) | 161.83(6) | V(2)#5—O(5)—V(3) | 98.67(5) |

**Symmetry transformations used to generate equivalent atoms:**

(i) x, 1+y, z; (ii) 0.5-x, 1.5-y, -z; (iii) 0.5-x, 2.5-y, -z; (iv) 1-x, 2-y, -z; (v) 1.5-x, 1.5-y, 1-z; (vi) x, -1+y, z; (vii) 0.5+x, -0.5+y, 1+z; (viii) x, 2-y, z; (ix) 1-x, 1-y, 1-z; (x) 1-x, 2-y, 1-z; (xi) 1-x, -1+y, 1-z; (xii) -0.5+x, 0.5+y, -1+z.

***Table S9. Crystal data and structure refinement for β'-$Cu_{0.65}V_2O_5$: bcuv2o5_062_0m.***

| | | |
|---|---|---|
| **Identification code** | *062bcuv2o5* | |
| **Empirical formula** | Cu0.65 O5 V2 | |
| **Formula weight** | 222.97 | |
| **Temperature** | 110.00 K | |
| **Wavelength** | 0.71073 Å | |
| **Crystal system** | Monoclinic | |
| **Space group** | *C 2/m* | |
| **Unit cell dimensions** | *a* = 15.1079(7) Å | α = 90°. |
| | *b* = 3.6372(2) Å | β = 105.671(2)° |
| | *c* = 10.0582(5) Å | γ = 90°. |
| **Volume** | 532.16(5) $Å^3$ | |
| ***Z*** | 6 | |
| **Density (calculated)** | 4.173 $mg/m^3$ | |
| **Absorption coefficient** | 8.913 $mm^{-1}$ | |
| **F(000)** | 628 | |
| **Crystal size** | 0.312 × 0.082 × 0.036 $mm^3$ | |
| **Theta range for data collection** | 2.103 to 38.777° | |
| **Index ranges** | -26<=h<=26, -6<=k<=6, -17<=l<=17 | |
| **Reflections collected** | 1699 | |
| **Independent reflections** | 1699 [R(int) = 0.0609] | |
| **Completeness to theta = 25.242°** | 99.20% | |
| **Absorption correction** | multi-scan | |
| **Max. and min. transmission** | 0.7476 and 0.5787 | |
| **Refinement method** | Full-matrix least-squares on $F^2$ | |
| **Data / restraints / parameters** | 1699 / 0 / 83 | |
| **Goodness-of-fit on $F^2$** | 1.393 | |
| **Final R indices [I>2sigma(I)]** | R1 = 0.0381, wR2 = 0.0476 | |
| **R indices (all data)** | R1 = 0.0255, wR2 = 0.0462 | |
| **Largest diff. peak and hole** | 0.866 and -1.170 e.$Å^{-3}$ | |

***Table S10. Atomic coordinates, occupancies, and equivalent isotropic displacement parameters ($Å^2$) for β'-$Cu_{0.65}V_2O_5$: bcuv2o5. U(eq) is defined as one third of the trace of the orthogonalized $U^{ij}$ tensor.***

| Atom | *x* | *y* | *z* | Occupancy | Uiso |
|---|---|---|---|---|---|
| Cu(1) | 0.4572(5) | 1.0000 | 0.1566(8) | 0.280 | 0.008(6) |
| Cu(2) | 0.4695(3) | 0.9552(16) | 0.1451(4) | 0.345 | 0.016(6) |
| V(1) | 0.28695(2) | 1.0000 | -0.09347(3) | 1 | 0.003(6) |
| V(2) | 0.66799(2) | 1.0000 | 0.41050(3) | 1 | 0.003(6) |
| V(3) | 0.61376(2) | 0.5000 | 0.62066(3) | 1 | 0.005(7) |
| O(1) | 0.25541(10) | 0.5000 | -0.07635(13) | 1 | 0.005(2) |

| | | | | | |
|---|---|---|---|---|---|
| O(2) | 0.39945(10) | 1.0000 | -0.03976(15) | 1 | 0.07(2) |
| O(3) | 0.26038(9) | 1.0000 | -0.27752(13) | 1 | 0.004(2) |
| O(4) | 0.56790(9) | 1.0000 | 0.29910(14) | 1 | 0.006(2) |
| O(5) | 0.68875(9) | 0.5000 | 0.45636(13) | 1 | 0.004(2) |
| O(6) | 0.5000 | 0.5000 | 0.5000 | 1 | 0.006(3) |
| O(7) | 0.63229(9) | 1.0000 | 0.58682(13) | 1 | 0.004(2) |
| O(8) | 0.59606(9) | 0.5000 | 0.77609(14) | 1 | 0.006(2) |

***Table S11. Crystal data and structure refinement for*** **β-*Pb*$_{0.01}$/β'-Cu0*$_{0.47}$V$_2$O$_5$*: *2674_1esc_1_0m,***

| | | |
|---|---|---|
| **Identification code** | 26741E1 | |
| **Empirical formula** | Cu0.47O5Pb0.01V2 | |
| **Formula weight** | 214.61 | |
| **Temperature** | 110.00 K | |
| **Wavelength** | 0.71073 Å | |
| **Crystal system** | Monoclinic | |
| **Space group** | *C 2/m* | |
| **Unit cell dimensions** | *a* = 15.1856(4) Å | α = 90° |
| | *b* = 3.62990(10) Å | β = 106.3300(10)° |
| | *c* = 10.0799(3) Å | γ = 90° |
| **Volume** | 533.21(3) Å$^3$ | |
| ***Z*** | 6 | |
| **Density (calculated)** | 4.010 mg/m$^3$ | |
| **Absorption coefficient** | 8.512 mm$^{-1}$ | |
| **F(000)** | 605 | |
| **Crystal size** | 0.177 × 0.091 × 0.079 mm$^3$ | |
| **Theta range for data collection** | 2.105 to 49.287° | |
| **Index ranges** | -32<=h<=32, -7<=k<=7, -21<=l<=21 | |
| **Reflections collected** | 29399 | |
| **Independent reflections** | 2974 [R(int) = 0.0430] | |
| **Completeness to theta = 49.287°** | 99.60% | |
| **Absorption correction** | multi-scan | |
| **Max. and min. transmission** | 0.7497 and 0.5150 | |
| **Refinement method** | Full-matrix least-squares on F$^2$ | |
| **Data / restraints / parameters** | 2974 / 0 / 89 | |
| **Goodness-of-fit on F$^2$** | 1.125 | |
| **Final R indices [I>2sigma(I)]** | R1 = 0.0232, wR2 = 0.0534 | |
| **R indices (all data)** | R1 = 0.0275, wR2 = 0.0552 | |
| **Largest diff. peak and hole** | 1.112 and 2.091 | |

***Table S12. Atomic coordinates, occupancies, and equivalent isotropic displacement parameters ($Å^2$) for β-$Pb_{0.01}$/β'-Cu0$_{.47}V_2O_5$: 2674_1esc_1_0m. U(eq) is defined as one third of the trace of the orthogonalized $U^{ij}$ tensor.***

| Atom | x | y | z | Occupancy | Uiso |
|---|---|---|---|---|---|
| Pb(1) | 0.49824(15) | 0.0000 | -0.1004(2) | 0.019 | 0.008(5) |
| Cu(1) | 0.5423(2) | 0.5000 | -0.1558(4) | 0.221 | 0.11(4) |
| Cu(2) | 0.5290(3) | 0.5648(15) | -0.1407(3) | 0.243 | 0.023(6) |
| V(1) | 0.66673(2) | 0.5000 | 0.40873(2) | 1 | 0.004(3) |
| V(2) | 0.61367(2) | 0.0000 | 0.62082(2) | 1 | 0.006(3) |
| V(3) | 0.71360(2) | 0.5000 | 0.09434(2) | 1 | 0.005(3) |
| O(1) | 0.74458(6) | 0.0000 | 0.07553(8) | 1 | 0.008(11) |
| O(2) | 0.60237(6) | 0.5000 | 0.04067(9) | 1 | 0.011(13) |
| O(3) | 0.73986(5) | 0.5000 | 0.27767(8) | 1 | 0.005(9) |
| O(4) | 0.56732(5) | 0.5000 | 0.29659(9) | 1 | 0.008(11) |
| O(5) | 0.68813(5) | 0.0000 | 0.45348(8) | 1 | 0.006(10) |
| O(6) | 0.5000 | 0.0000 | 0.5000 | 1 | 0.007(15) |
| O(7) | 0.63237(5) | 0.5000 | 0.58470(8) | 1 | 0.005(9) |
| O(8) | 0.59746(6) | 0.0000 | 0.77491(9) | 1 | 0.009(12) |

***Table S13. Anisotropic displacement parameters ($Å^2$x $10^3$) for β-$Pb_{0.01}$/β'-$Cu_{0.47}V_2O_5$: 2674_1esc_1_0m. The anisotropic displacement factor exponent takes the form: -2[illegible]$^2$[ $h^2$ $a^{*2}U^{11}$ + ... + 2 h k a* b* $U^{12}$]***

| Atom | U11 | U22 | U33 | U23 | U13 | U12 |
|---|---|---|---|---|---|---|
| Pb(1) | 6(7) | 9(8) | 7(7) | 0 | 1(5) | 0 |
| Cu(1) | 8(5) | 16(7) | 6(4) | 0 | -3(4) | 0 |
| Cu(2) | 17(6) | 36(10) | 9(4) | 6(5) | -8(4) | -12(7) |
| V(1) | 9(6) | 4(5) | 3(5) | 0 | 2(4) | 0 |
| V(2) | 4(5) | 3(5) | 4(5) | 0 | 1(4) | 0 |
| V(3) | 10(6) | 3(5) | 6(5) | 0 | 5(4) | 0 |
| O(1) | 15(3) | 4(2) | 5(2) | 0 | 5(2) | 0 |
| O(2) | 10(3) | 15(4) | 7(3) | 0 | -1(2) | 0 |
| O(3) | 7(2) | 5(2) | 4(2) | 0 | 1(18) | 0 |
| O(4) | 6(2) | 10(3) | 7(2) | 0 | -1(19) | 0 |
| O(5) | 7(2) | 4(2) | 6(2) | 0 | 2(19) | 0 |
| O(6) | 4(3) | 5(3) | 11(4) | 0 | 1(3) | 0 |

| | | | | | | |
|---|---|---|---|---|---|---|
| O(7) | 7(2) | 3(2) | 6(2) | 0 | 2(19) | 0 |
| O(8) | 9(3) | 15(3) | 5(2) | 0 | 3(2) | 0 |

***Table S14. Bond lengths [Å] and angles [°] for β-$Pb_{0.01}$/β'-$Cu_{0.47}V_2O_5$: 2674_1esc_1_0m.***

| Atom pairs | Bond Length [Å] | Atom pairs | Bond Length [Å] |
|---|---|---|---|
| V(1)—V(3) | 3.2998(4) | V(3)—O(7) | 1.8882(2) |
| V(2)—V(3)#1 | 3.0793(2) | V(3)—O(5) | 2.2795(8) |
| V(2)—V(3) | 3.0793(2) | V(3)—O(8) | 1.6396(8) |
| V(2)—O(4) | 1.6116(8) | V(1)—V(1)#4 | 3.0521(3) |
| V(2)—O(3) | 1.9503(8) | V(1)—V(1)#5 | 3.0521(3) |
| V(2)—O(7) | 1.9847(8) | V(1)—O(3) | 1.7775(7) |
| V(2)—O(5)#1 | 1.8763(2) | V(1)—O(2) | 1.6223(10) |
| V(2)—O(5) | 1.8763(2) | V(1)—O(1)#1 | 1.8977(3) |
| V(2)—O(5)#2 | 2.2506(8) | V(1)—O(1)#5 | 1.9866(8) |
| V(3)—O(3)#2 | 2.1712(8) | V(1)—O(1) | 1.8977(3) |
| V(3)—O(6) | 1.8112(2) | V(3)—O(7)#3 | 1.8882(2) |

| Atoms | Bond Angle [°] | Atoms | Bond Angle [°] |
|---|---|---|---|
| O(5)—V(2)—O(7) | 81.75(2) | O(5)—V(3)—V(2) | 37.364(6) |
| V(3)#1—V(2)—V(3) | 72.228(5) | O(5)—V(3)—V(2)#3 | 37.364(6) |
| O(4)—V(2)—V(3)#1 | 96.34(3) | O(8)—V(3)—V(2) | 142.748(8) |
| O(4)—V(2)—V(3) | 96.34(3) | O(8)—V(3)—V(2)#3 | 142.748(8) |
| O(4)—V(2)—O(3) | 97.15(4) | O(8)—V(3)—O(3)#2 | 87.72(4) |
| O(4)—V(2)—O(7) | 101.36(4) | O(8)—V(3)—O(6) | 105.58(3) |
| O(4)—V(2)—O(5) | 103.49(2) | O(8)—V(3)—O(7)#3 | 104.68(2) |
| O(4)—V(2)—O(5)#1 | 103.49(2) | O(8)—V(3)—O(7) | 104.68(2) |
| O(4)—V(2)—O(5)#2 | 174.01(4) | O(8)—V(3)—O(5) | 159.85(4) |
| O(3)—V(2)—V(3) | 141.286(8) | V(1)#5—V(1)—V(1)#4 | 72.978(8) |
| O(3)—V(2)—V(3)#1 | 141.286(8) | O(3)—V(1)—V(1)#4 | 129.280(18) |
| O(3)—V(2)—O(7) | 161.49(3) | O(3)—V(1)—V(1)#5 | 129.280(18) |
| O(3)—V(2)—O(5)#2 | 76.86(3) | O(3)—V(1)—O(1)#1 | 96.57(3) |
| O(7)—V(2)—V(3)#1 | 36.260(3) | O(3)—V(1)—O(1) | 96.57(3) |
| O(7)—V(2)—V(3) | 36.260(3) | O(3)—V(1)—O(1)#5 | 149.70(4) |
| O(7)—V(2)—O(5)#2 | 84.63(3) | O(2)—V(1)—V(1)#5 | 108.53(3) |
| O(5)—V(2)—V(3)#1 | 117.69(2) | O(2)—V(1)—V(1)#4 | 108.53(3) |
| O(5)#2—V(2)—V(3) | 88.486(17) | O(2)—V(1)—O(3) | 104.77(4) |
| O(5)#1—V(2)—V(3) | 117.69(2) | O(2)—V(1)—O(1)#1 | 103.34(3) |
| O(5)—V(2)—V(3) | 47.50(2) | O(2)—V(1)—O(1) | 103.34(3) |
| O(5)#1—V(2)—V(3)#1 | 47.50(2) | O(2)—V(1)—O(1)#5 | 105.53(4) |
| O(5)#2—V(2)—V(3)#1 | 88.486(17) | O(1)#1—V(1)—V(1)#4 | 39.25(2) |
| O(5)—V(2)—O(3) | 94.00(2) | O(1)#5—V(1)—V(1)#5 | 37.191(6) |
| O(5)#1—V(2)—O(3) | 94.00(2) | O(1)—V(1)—V(1)#4 | 111.29(2) |
| O(5)#1—V(2)—O(7) | 81.75(2) | O(1)#1—V(1)—V(1)#5 | 111.29(2) |
| O(5)#1—V(2)—O(5)#2 | 77.19(2) | O(1)—V(1)—V(1)#5 | 39.25(2) |
| O(5)#1—V(2)—O(5) | 150.61(5) | V(2)#3—O(5)—V(2) | 150.61(5) |
| O(5)—V(2)—O(5)#2 | 77.19(2) | V(2)#3—O(5)—V(2)#2 | 102.81(2) |
| V(2)—V(3)—V(2)#3 | 72.228(5) | V(2)—O(5)—V(3) | 95.13(2) |
| O(3)#2—V(3)—V(2) | 83.903(17) | V(2)#2—O(5)—V(3) | 98.44(3) |
| O(3)#2—V(3)—V(2)#3 | 83.903(17) | V(2)#3—O(5)—V(3) | 95.14(2) |

| | | | |
|---|---|---|---|
| O(3)#2—V(3)—O(5) | 72.13(3) | V(1)#3—O(1)—V(1) | 146.03(5) |
| O(6)—V(3)—V(2)#3 | 85.364(6) | V(1)#3—O(1)—V(1)#5 | 103.55(2) |
| O(6)—V(3)—V(2) | 85.364(6) | V(1)—O(1)—V(1)#5 | 103.55(2) |
| O(6)—V(3)—O(3)#2 | 166.70(2) | O(6)—V(3)—O(7)#3 | 92.12(2) |
| O(6)—V(3)—O(7) | 92.12(2) | O(6)—V(3)—O(5) | 94.57(2) |

**Symmetry transformations used to generate equivalent atoms:**
(i) x, 1+y, z; (ii) 1.5-x, 0.5-y, 1-z; (iii) x, -1+y, z; (iv) 1.5-x, 1.5-y, -z;
(v) 1.5-x, 0.5-y, -z; (vi) 1-x, -y, 1-z.

***Table S15. Crystal data and structure refinement for β-$Pb_{0.08}$/β'-$Cu_{0.33}V_2O_5$: 2674_1csc_1_0m.***

| | | |
|---|---|---|
| **Identification code** | 26741C1 | |
| **Empirical formula** | Cu0.33O5Pb0.08V2 | |
| **Formula weight** | 219.42 | |
| **Temperature** | 110.00 K | |
| **Wavelength** | 0.71073 Å | |
| **Crystal system** | Monoclinic | |
| **Space group** | *C 2/m* | |
| **Unit cell dimensions** | *a* = 15.3408(6) Å | α = 90°. |
| | *b* = 3.6293(2) Å | β = 107.458(2)° |
| | *c* = 10.0934(4) Å | γ = 90°. |
| **Volume** | 536.08(4) $Å^3$ | |
| ***Z*** | 6 | |
| **Density (calculated)** | 4.078 mg/$m^3$ | |
| **Absorption coefficient** | 10.771 $mm^{-1}$ | |
| **F(000)** | 613 | |
| **Crystal size** | 0.143 × 0.062 × 0.048 $mm^3$ | |
| **Theta range for data collection** | 2.78 to 52.29° | |
| **Index ranges** | -34<=h<=34, -8<=k<=8, -20<=l<=22 | |
| **Reflections collected** | 32984 | |
| **Independent reflections** | 3367 [R(int) = 0.0355] | |
| **Completeness to theta = 52.322°** | 99.30% | |
| **Absorption correction** | multi-scan | |
| **Max. and min. transmission** | 0.7503 and 0.4667 | |
| **Refinement method** | Full-matrix least-squares on $F^2$ | |
| **Data / restraints / parameters** | 3367 / 0 / 89 | |
| **Goodness-of-fit on $F^2$** | 1.231 | |
| **Final R indices [I>2sigma(I)]** | R1 = 0.0222, wR2 = 0.0525 | |
| **R indices (all data)** | R1 = 0.0246, wR2 = 0.0535 | |
| **Largest diff. peak and hole** | 1.885 and -1.822 | |

***Table S16. Atomic coordinates, occupancies, and equivalent isotropic displacement parameters ($Å^2$) for β-$Pb_{0.08}$/β'-$Cu_{0.33}V_2O_5$. Crystal data and structure refinement for β-$Pb_{0.08}$/β'-$Cu_{0.33}V_2O_5$: 2674_1csc_1_0m. U(eq) is defined as one third of the trace of the orthogonalized $U^{ij}$ tensor.***

| Atom | *x* | *y* | *z* | Occupancy | Uiso |
|---|---|---|---|---|---|
| Pb(1) | 0.49984(2) | 0.5000 | 0.60375(4) | 0.119 | 0.008(7) |
| Cu(1) | 0.4550(2) | 1.0000 | 0.6594(4) | 0.147 | 0.010(8) |

| | | | | | |
|---|---|---|---|---|---|
| Cu(2) | 0.4664(4) | 0.930(2) | 0.6453(5) | 0.174 | 0.023(10) |
| V(1) | 0.28539(2) | 1.0000 | 0.40589(2) | 1 | 0.005(3) |
| V(2) | 0.66615(2) | 1.0000 | 0.90609(2) | 1 | 0.004(2) |
| V(3) | 0.61484(2) | 0.5000 | 1.11867(2) | 1 | 0.006(3) |
| O(1) | 0.25505(7) | 0.5000 | 0.42434(9) | 1 | 0.008(11) |
| O(2) | 0.39572(7) | 1.0000 | 0.46285(10) | 1 | 0.011(14) |
| O(3) | 0.26072(5) | 1.0000 | 0.22192(8) | 1 | 0.005(9) |
| O(4) | 0.56693(6) | 1.0000 | 0.79159(9) | 1 | 0.009(12) |
| O(5) | 0.68767(6) | 0.5000 | 0.95150(9) | 1 | 0.006(10) |
| O(6) | 0.500000 | 0.5000 | 1.0000 | 1 | 0.007(15) |
| O(7) | 0.63279(5) | 1.0000 | 1.08135(8) | 1 | 0.005(9) |
| O(8) | 0.60168(7) | 0.5000 | 1.27354(9) | 1 | 0.010(13) |

***Table S17. Anisotropic displacement parameters (Å$^2$x 10$^3$) for Table S1. Crystal data and structure refinement for β-$Pb_{0.08}$/β'-$Cu_{0.33}V_2O_5$: 2674_1csc_1_0m. The anisotropic displacement factor exponent takes the form: $-2\pi^2[ h^2 a^{*2}U^{11} + ... + 2 h k a^* b^* U^{12}]$***

| Atom | U11 | U22 | U33 | U23 | U13 | U12 |
|---|---|---|---|---|---|---|
| Pb(1) | 6(11) | 9(13) | 8(12) | 0 | 1(8) | 0 |
| Cu(1) | 6(7) | 15(14) | 6(6) | 0 | -3(4) | 0(1) |
| Cu(2) | 19(9) | 37(7) | 12(6) | 7(9) | -10(7) | 11(12) |
| V(1) | 8(6) | 3(5) | 4(5) | 0 | 2(4) | 0 |
| V(2) | 5(5) | 2(5) | 5(5) | 0 | 2(4) | 0 |
| V(3) | 10(6) | 2(5) | 7(5) | 0 | 5(4) | 0 |
| O(1) | 15(3) | 3(2) | 6(2) | 0 | 4(2) | 0 |
| O(2) | 10(3) | 15(4) | 7(3) | 0 | -1(2) | 0 |
| O(3) | 6(2) | 5(2) | 5(2) | 0 | 1(18) | 0 |
| O(4) | 6(2) | 12(3) | 9(2) | 0 | 3(2) | 0 |
| O(5) | 7(2) | 4(2) | 7(2) | 0 | 2(19) | 0 |
| O(6) | 4(3) | 5(3) | 11(4) | 0 | 1(3) | 0 |
| O(7) | 7(2) | 4(2) | 6(2) | 0 | 3(19) | 0 |
| O(8) | 11(3) | 15(4) | 6(3) | 0 | 3(2) | 0 |

***Table S18. Bond lengths [Å] and angles [°] for Table S1. Crystal data and structure refinement for β-$Pb_{0.08}$/β'-$Cu_{0.33}V_2O_5$: 2674_1csc_1_0m.***

| Atom pairs | Bond Length [Å] | Atom pairs | Bond Length [Å] |
|---|---|---|---|
| V(2)—V(3) | 3.0870(2) | V(1)—O(1) | 1.616(1) |
| V(2)—V(3)#1 | 3.0870(2) | V(1)—O(1)#5 | 1.9898(9) |
| V(2)—O(4) | 1.6115(9) | V(1)—O(1)#1 | 1.8964(3) |
| V(2)—O(3)#2 | 1.9488(8) | V(1)—O(1) | 1.8964(3) |
| V(2)—O(5)#1 | 1.8765(2) | V(3)—O(3)#6 | 2.1667(8) |
| V(2)—O(5) | 1.8765(2) | V(3)—O(6) | 1.8090(2) |
| V(2)—O(5)#3 | 2.2718(9) | V(3)—O(5) | 2.2861(9) |
| V(2)—O(7) | 1.9836(8) | V(3)—O(7) | 1.8902(2) |
| V(1)—V(1)#4 | 3.0527(3) | V(3)—O(7)#7 | 1.8902(2) |
| V(1)—V(3) | 3.2709(4) | V(3)—O(8) | 1.6352(9) |

| Atoms | Bond Angle [°] | Atoms | Bond Angle [°] |
|---|---|---|---|
| | | O(3)#6—V(3)—V(2) | 83.824(18) |
| V(3)#1—V(2)—V(3) | 72.007(6) | O(3)#6—V(3)—V(2)#7 | 83.824(18) |
| O(4)—V(2)—V(3) | 96.64(3) | O(3)#6—V(3)—O(5) | 72.07(3) |
| O(4)—V(2)—V(3)#1 | 96.64(3) | O(6)—V(3)—V(2)#7 | 86.592(7) |
| O(4)—V(2)—O(3)#2 | 97.60(4) | O(6)—V(3)—V(2) | 86.591(7) |
| O(4)—V(2)—O(5) | 103.55(3) | O(6)—V(3)—O(3)#6 | 168.14(2) |
| O(4)—V(2)—O(5)#3 | 173.96(4) | O(6)—V(3)—O(5) | 96.08(2) |
| O(4)—V(2)—O(5)#1 | 103.55(3) | O(6)—V(3)—O(7) | 92.25(3) |
| O(4)—V(2)—O(7) | 101.46(4) | O(6)—V(3)—O(7)#7 | 92.25(3) |
| O(3)#2—V(2)—V(3) | 141.110(9) | O(5)—V(3)—V(2)#7 | 37.253(6) |
| O(3)#2—V(2)—V(3)#1 | 141.111(9) | O(5)—V(3)—V(2) | 37.254(6) |
| O(3)#2—V(2)—O(5)#3 | 76.36(3) | O(7)#7—V(3)—V(2) | 110.07(2) |
| O(3)#2—V(2)—O(7) | 160.94(3) | O(7)—V(3)—V(2)#7 | 110.07(2) |
| O(5)—V(2)—V(3)#1 | 117.49(3) | O(7)#8—V(3)—V(2)#7 | 38.23(2) |
| O(5)#3—V(2)—V(3) | 88.241(18) | O(7)—V(3)—V(2) | 38.22(2) |
| O(5)#3—V(2)—V(3)#1 | 88.241(18) | O(7)—V(3)—O(3)#6 | 84.53(3) |
| O(5)—V(2)—V(3) | 47.52(3) | O(7)#7—V(3)—O(3)#6 | 84.53(3) |
| O(5)#1—V(2)—V(3)#1 | 47.52(3) | O(7)#7—V(3)—O(5) | 73.76(2) |
| O(5)#1—V(2)—V(3) | 117.49(3) | O(7)—V(3)—O(5) | 73.76(2) |
| O(5)—V(2)—O(3)#2 | 93.89(3) | O(7)#7—V(3)—O(7) | 147.50(5) |
| O(5)#1—V(2)—O(3)#2 | 93.89(3) | O(8)—V(3)—V(2) | 142.64(1) |
| O(5)#—V(2)—O(5) | 150.50(5) | O(8)—V(3)—V(2)#7 | 142.64(1) |
| O(5)#1—V(2)—O(5)#3 | 77.14(3) | O(8)—V(3)—O(3)#6 | 86.91(4) |
| O(5)—V(2)—O(5)#3 | 77.14(3) | O(8)—V(3)—O(6) | 104.94(4) |
| O(5)#1—V(2)—O(7) | 81.71(3) | O(8)—V(3)—O(5) | 158.98(4) |
| O(5)—V(2)—O(7) | 81.71(3) | O(8)—V(3)—O(7)#7 | 104.93(2) |
| O(7)—V(2)—V(3)#1 | 36.130(4) | O(8)—V(3)—O(7) | 104.93(2) |
| O(7)—V(2)—V(3) | 36.130(4) | V(2)#2—O(7)—V(3)#8 | 113.46(4) |
| O(7)—V(2)—O(5)#3 | 84.58(3) | V(1)—O(7)—V(2)#2 | 134.99(5) |
| O(3)—V(1)—V(1)#4 | 130.170(18) | V(1)—O(3)—V(3)#8 | 111.55(4) |
| O(3)—V(1)—O(1)#1 | 96.94(3) | V(3)#9—O(6)—V(3) | 180.000 |
| O(3)—V(1)—O(1) | 96.94(3) | V(2)—O(5)—V(2)#3 | 102.86(3) |
| O(3)—V(1)—O(1)#5 | 150.99(4) | V(2)#7—O(5)—V(2) | 150.50(5) |

| | | | |
|---|---|---|---|
| O(2)—V(1)—V(1)#4 | 107.99(3) | V(2)#7—O(5)—V(2)#3 | 102.86(3) |
| O(2)—V(1)—O(3) | 104.08(4) | V(2)#7—O(5)—V(3) | 95.23(3) |
| O(2)—V(1)—O(1)#5 | 104.92(5) | V(2)—O(5)—V(3) | 95.23(3) |
| O(2)—V(1)—O(1)#1 | 103.11(3) | V(2)#3—O(5)—V(3) | 98.12(3) |
| O(2)—V(1)—O(1) | 103.11(3) | V(3)—O(7)—V(2) | 105.64(2) |
| O(1)—V(1)—V(1)#4 | 111.35(3) | V(3)#1—O(7)—V(2) | 105.64(2) |
| O(1)#1—V(1)—V(1)#4 | 39.33(2) | V(3)—O(7)—V(3)#1 | 147.49(5) |
| O(1)#5—V(1)—V(1)#4 | 37.158(7) | V(1)—O(1)—V(1)#5 | 103.51(3) |
| O(1)—V(1)—O(1)#5 | 76.49(3) | V(1)#7—O(1)—V(1)#5 | 103.51(3) |
| O(1)#1—V(1)—O(1) | 146.24(6) | V(1)—O(1)—V(1)#7 | 146.24(5) |
| O(1)#1—V(1)—O(1)#5 | 76.49(3) | V(2)—V(3)—V(2)#7 | 72.007(6) |

**Symmetry transformations used to generate equivalent atoms:**

(i) x, 1+y, z; (ii) 1-x, 2-y, 1-z; (iii) 1.5-x, 1.5-y, 2-z; (iv) 0.5-x, 2.5-y, 1-z;
(v) 0.5-x, 1.5-y, 1-z; (vi) 0.5+x, -0.5+y, 1+z; (vii) x, -1+y, z; (viii) -0.5+x, 0.5+y, -1+z;
(ix) 1-x, 1-y, 2-z.

***Table S19. Crystal data and structure refinement for β-$Pb_{0.11}$/β'-$Cu_{0.41}V_2O_5$: pbcuv2o5_3481b_0m_a.***

| | | |
|---|---|---|
| **Identification code** | 3481bpbcuv2o5 | |
| **Empirical formula** | Cu0.41O5Pb0.11V2 | |
| **Formula weight** | 230.72 | |
| **Temperature** | 110.00 K | |
| **Wavelength** | 0.71073 Å | |
| **Crystal system** | Monoclinic | |
| **Space group** | *C 2/m* | |
| **Unit cell dimensions** | *a* = 15.3078(14) Å | α = 90°. |
| | *b* = 3.6340(3) Å | β = 107.451(3)° |
| | *c* = 10.0911(9) Å | γ = 90°. |
| **Volume** | 535.52(8) Å$^3$ | |
| ***Z*** | 6 | |
| **Density (calculated)** | 4.288 mg/m$^3$ | |
| **Absorption coefficient** | 12.606 mm$^{-1}$ | |
| **F(000)** | 641 | |
| **Crystal size** | 0.125 × 0.04 × 0.01 mm$^3$ | |
| **Theta range for data collection** | 2.116 to 40.337° | |
| **Index ranges** | -27<=h<=27, -6<=k<=6, -18<=l<=18 | |
| **Reflections collected** | 1880 | |
| **Independent reflections** | 1880 [R(int) = 0.0682] | |
| **Completeness to theta = 25.242°** | 99.30% | |
| **Absorption correction** | multi-scan | |
| **Max. and min. transmission** | 0.5045 and 0.7479 | |
| **Refinement method** | Full-matrix least-squares on F$^2$ | |
| **Data / restraints / parameters** | 1880 / 0 / 90 | |
| **Goodness-of-fit on F$^2$** | 1.09 | |
| **Final R indices [I>2sigma(I)]** | R1 = 0.063, wR2 = 0.0879 | |
| **R indices (all data)** | R1 = 0.0373, wR2 = 0.0801 | |
| **Largest diff. peak and hole** | 1.516 and 1.368 | |

***Table S20. Atomic coordinates, occupancies, and equivalent isotropic displacement parameters (Å$^2$) for β-$Pb_{0.11}$/β'-$Cu_{0.41}V_2O_5$: pbcuv2o5_3481b_0m_a. U(eq) is defined as one third of the trace of the orthogonalized $U^{ij}$ tensor.***

| Atom | x | y | z | Occupancy | Uiso |
|---|---|---|---|---|---|
| Pb(1) | 0.49986(5) | 0.5000 | -0.10874(8) | 0.164 | 0.011(19) |
| Cu(1) | 0.5498(9) | 1.0000 | -0.1679(15) | 0.130 | 0.008(3) |
| Cu(2) | 0.5369(7) | 0.949(4) | -0.1504(9) | 0.243 | 0.026(17) |
| V(1) | 0.71466(4) | 1.0000 | 0.09337(5) | 1 | 0.008(11) |
| V(2) | 0.66697(4) | 1.0000 | 0.40654(5) | 1 | 0.007(10) |
| V(3) | 0.61575(4) | 0.5000 | 0.61731(5) | 1 | 0.009(11) |
| O(1) | 0.75517(18) | 1.0000 | -0.0762(2) | 1 | 0.011(4) |
| O(2) | 0.60299(18) | 1.0000 | 0.0349(3) | 1 | 0.013(4) |
| O(3) | 0.73805(16) | 1.0000 | 0.2779(2) | 1 | 0.008(4) |
| O(4) | 0.56703(16) | 1.0000 | 0.2920(3) | 1 | 0.012(4) |
| O(5) | 0.68786(16) | 0.5000 | 0.4536(2) | 1 | 0.009(4) |
| O(6) | 0.5000 | 0.5000 | 0.5000 | 1 | 0.010(5) |
| O(7) | 0.63326(16) | 1.0000 | 0.5818(2) | 1 | 0.009(4) |
| O(8) | 0.60279(18) | 0.5000 | 0.7737(2) | 1 | 0.012(4) |

***Table S21. Anisotropic displacement parameters (Å$^2$x $10^3$) for β-$Pb_{0.11}$/β'-$Cu_{0.41}V_2O_5$: pbcuv2o5_3481b_0m_a. The anisotropic displacement factor exponent takes the form: -2[illegible]$^2$[ $h^2$ $a^{*2}U^{11}$ + ... + 2 h k a* b* $U^{12}$]***

| Atom | U11 | U22 | U33 | U23 | U13 | U12 |
|---|---|---|---|---|---|---|
| Pb(1) | 10(3) | 12(3) | 11(3) | 0 | 3(2) | 0 |
| Cu(1) | 6(3) | 9(4) | 5(2) | 0 | -3(13) | 0(1) |
| Cu(2) | 22(18) | 35(3) | 14(13) | -3(15) | -7(13) | 7(19) |
| V(1) | 11(5) | 5(2) | 9(2) | 0 | 3(2) | 0 |
| V(2) | 8(19) | 4(2) | 10(19) | 0 | 2(15) | 0 |
| V(3) | 14(2) | 4(2) | 11(2) | 0 | 6(17) | 0 |
| O(1) | 17(10) | 7(9) | 9(8) | 0 | 4(8) | 0 |
| O(2) | 12(10) | 15(12) | 11(9) | 0 | 0(8) | 0 |
| O(3) | 9(8) | 6(9) | 9(8) | 0 | 2(7) | 0 |
| O(4) | 9(9) | 11(11) | 14(9) | 0 | 2(8) | 0 |
| O(5) | 10(9) | 6(9) | 10(8) | 0 | 1(7) | 0 |
| O(6) | 9(12) | 5(13) | 17(13) | 0 | 5(10) | 0 |

| | | | | | | |
|---|---|---|---|---|---|---|
| O(7) | 11(9) | 5(9) | 11(9) | 0 | 3(7) | 0 |
| O(8) | 18(11) | 10(10) | 10(9) | 0 | 3(8) | 0 |

***Table S22. Bond lengths [Å] and angles [°] for β-$Pb_{0.11}$/β'-$Cu_{0.41}V_2O_5$: pbcuv2o5_3481b_0m_a.***

| Atom pairs | Bond Length [Å] | Atom pairs | Bond Length [Å] |
|---|---|---|---|
| Pb(1)—Cu(2) | 1.817(18) | V(2)—O(5)#10 | 2.247(2) |
| Pb(1)—Cu(2) | 1.817(18) | V(2)—V(3)#11 | 3.0724(6) |
| Pb(1)—Cu(1)#2 | 2.124(9) | V(2)—V(3) | 3.0724(6) |
| Pb(1)—Cu(2)#3 | 2.157(9) | V(3)—O(8) | 1.649(2) |
| Pb(1)—Cu(2)#2 | 2.157(9) | V(3)—O(6) | 1.8118(6) |
| Pb(1)—Pb(1)#4 | 2.1934(15) | V(3)—O(7) | 1.8866(7) |
| Pb(1)—O(8)#5 | 2.238(3) | V(3)—O(7)#2 | 1.8866(7) |
| Pb(1)—O(2) | 2.5541(18) | V(3)—O(3)#10 | 2.170(2) |
| Pb(1)—O(2)#2 | 2.5541(18) | V(3)—O(5) | 2.246(2) |
| Pb(1)—O(4)#4 | 2.5754(18) | O(2)—Cu(2) | 1.849(5) |
| Pb(1)—O(4)#6 | 2.5754(18) | O(2)—Cu(2)#3 | 1.849(5) |
| V(2)—O(2) | 1.632(3) | O(2)—Cu(1) | 1.961(14) |
| V(2)—O(3) | 1.788(2) | O(4)—Cu(2)#11 | 1.801(4) |
| V(1)—O(1)#7 | 1.8954(8) | O(4)—Cu(2)#6 | 1.801(4) |
| V(1)—O(1)#8 | 1.8954(8) | O(4)—Cu(1)#6 | 1.851(11) |
| V(1)—O(1) | 1.988(2) | O(4)—Cu(1)#8 | 1.851(11) |
| V(1)—V(1)#8 | 3.0408(9) | O(8)—Cu(1)#7 | 2.144(10) |
| V(1)—V(1)#7 | 3.0408(9) | O(8)—Cu(1)#8 | 2.144(10) |
| V(1)—Cu(1) | 3.053(11) | O(4)—Cu(2)#14 | 2.175(7) |
| V(1)—Cu(2)#3 | 3.078(5) | O(4)—Cu(2)#13 | 2.175(7) |
| V(1)—Cu(2) | 3.078(5) | V(1)—V(3) | 3.2738(7) |
| V(2)—O(4) | 1.618(2) | V(2)—O(5) | 1.8810(6) |

| Atoms | Bond Angle [°] | Atoms | Bond Angle [°] |
|---|---|---|---|
| Cu(2)—Pb(1)—Cu(2)#1 | 127.7(7) | O(7)#2—V(3)—O(3)#10 | 84.72(8) |
| Cu(2)—Pb(1)—Cu(1)#2 | 122.7(7) | O(8)—V(3)—O(5) | 158.63(11) |
| Cu(2)#1—Pb(1)—Cu(1)#2 | 5.1(3) | O(6)—V(3)—O(5) | 96.86(6) |
| Cu(2)—Pb(1)—Cu(2)#3 | 4.3(3) | O(7)—V(3)—O(5) | 74.40(7) |
| Cu(2)#1—Pb(1)—Cu(2)#3 | 132.1(8) | O(7)#2—V(3)—O(5) | 74.40(7) |
| Cu(2)—Pb(1)—Cu(2)#2 | 132.1(8) | O(3)#10—V(3)—O(5) | 72.28(9) |
| Cu(2)#1—Pb(1)—Cu(2)#2 | 4.3(3) | O(8)—V(3)—V(2)#2 | 142.34(3) |
| Cu(2)#3—Pb(1)—Cu(2)#2 | 136.4(10) | O(6)—V(3)—V(2)#2 | 87.15(2) |
| Cu(2)—Pb(1)—Pb(1)#4 | 108.9(2) | O(7)—V(3)—V(2)#2 | 110.97(7) |
| Cu(2)#1—Pb(1)—Pb(1)#4 | 108.9(2) | O(7)#2—V(3)—V(2)#2 | 38.62(7) |
| Cu(2)#3—Pb(1)—Pb(1)#4 | 105.8(4) | O(3)#10—V(3)—V(2)#2 | 84.10(5) |
| Cu(2)#2—Pb(1)—Pb(1)#4 | 105.8(4) | O(5)—V(3)—V(2)#2 | 37.521(17) |
| Cu(2)—Pb(1)—O(8)#5 | 63.9(3) | O(8)—V(3)—V(2) | 142.34(3) |
| Cu(2)#1—Pb(1)—O(8)#5 | 63.9(3) | O(6)—V(3)—V(2) | 87.15(2) |
| Cu(2)#3—Pb(1)—O(8)#5 | 68.2(5) | O(7)—V(3)—V(2) | 38.62(7) |
| Cu(2)#2—Pb(1)—O(8)#5 | 68.2(5) | O(7)#2—V(3)—V(2) | 110.96(7) |
| Pb(1)#4—Pb(1)—O(8)#5 | 137.71(8) | O(3)#10—V(3)—V(2) | 84.10(5) |
| Cu(2)—Pb(1)—O(2) | 46.35(15) | O(5)—V(3)—V(2) | 37.520(17) |
| Cu(2)#1—Pb(1)—O(2) | 126.0(2) | V(2)#2—V(3)—V(2) | 72.513(18) |
| Cu(1)—Pb(1)—O(2) | 48.5(4) | V(1)#7—O(1)—V(1)#8 | 146.93(15) |
| Cu(1)#2—Pb(1)—O(2) | 123.7(3) | V(1)#7—O(1)—V(1) | 103.06(7) |
| Cu(2)#3—Pb(1)—O(2) | 45.25(15) | V(1)#8—O(1)—V(1) | 103.06(7) |

| | | | |
|---|---|---|---|
| Cu(2)#2—Pb(1)—O(2) | 128.1(3) | V(2)#2—O(5)—V(2) | 150.03(14) |
| Pb(1)#4—Pb(1)—O(2) | 67.46(6) | V(2)#2—O(5)—V(3) | 95.84(8) |
| O(8)#5—Pb(1)—O(2) | 83.53(8) | V(2)—O(5)—V(3) | 95.84(8) |
| Cu(2)—Pb(1)—O(2)#2 | 126.0(2) | V(2)#2—O(5)—V(2)#10 | 102.70(7) |
| Cu(2)#1—Pb(1)—O(2)#2 | 46.35(15) | V(2)—O(5)—V(2)#10 | 102.70(7) |
| Cu(2)#3—Pb(1)—O(2)#2 | 128.1(3) | V(3)—O(5)—V(2)#10 | 98.60(9) |
| Cu(2)#2—Pb(1)—O(2)#2 | 45.25(15) | V(3)#15—O(6)—V(3) | 180.00(5) |
| Pb(1)#4—Pb(1)—O(2)#2 | 67.46(6) | V(3)—O(7)—V(3)#9 | 148.77(14) |
| O(8)#5—Pb(1)—O(2)#2 | 83.53(8) | V(3)—O(7)—V(2) | 105.00(7) |
| O(2)—Pb(1)—O(2)#2 | 90.70(8) | V(3)#9—O(7)—V(2) | 105.00(7) |
| Cu(2)—Pb(1)—O(4)#4 | 123.5(2) | V(3)—O(8)—Cu(1)#13 | 115.3(4) |
| Cu(2)#1—Pb(1)—O(4)#4 | 44.37(13) | V(3)—O(8)—Cu(2)#14 | 122.6(4) |
| V(1)—O(3)—V(2) | 136.42(13) | V(3)—O(8)—Cu(2)#13 | 122.6(4) |
| V(1)—O(3)—V(3)#10 | 111.26(11) | Cu(2)#14—O(8)—Cu(2) | 97.2(10) |
| V(2)—O(3)—V(3)#10 | 112.32(11) | V(3)—O(8)—Pb(1)#13 | 144.41(15) |
| V(3)—O(8)—Cu(1)#12 | 115.3(4) | Cu(2)—O(8)—Pb(1)#13 | 48.6(5) |

**Symmetry transformations used to generate equivalent atoms:**
(i) x, 1-y, z; (ii) x, -1+y, z; (iii) x, 2-y, z; (iv) 1-x, 1-y, -z;
(v) x, y, -1+z; (vi) 1-x, 2-y, -z; (vii) 1.5-x, 2.5-y, -z; (viii) 1.5-x, 1.5-y, -z;
(ix) x, 1+y, z; (x) 1.5-x, 1.5-y, 1-z; (xi) 1-x, y, -z; (xii) x, -1+y, 1+z;
(xiii) x, y, 1+z; (xiv) x, 1-y, 1+z; (xv) 1-x, 1-y, 1-z; (xvi) x, 1+y, -1+z.

***Table S23. Crystal data and structure refinement for β-$Pb_{0.115}V_2O_5$: pbv2o5_4511b_0m_a.***

| | | |
|---|---|---|
| **Identification code** | 4511bpbv2o5 | |
| **Empirical formula** | Pb0.115V2O5 | |
| **Formula weight** | 205.48 | |
| **Temperature** | 110.00 K | |
| **Wavelength** | 0.71073 Å | |
| **Crystal system** | Monoclinic | |
| **Space group** | C 2/m | |
| **Unit cell dimensions** | *a* = 15.3440(12) Å | α = 90°. |
| | *b* = 3.6112(3) Å | β = 109.755(2)° |
| | *c* = 10.0331(8) Å | γ = 90°. |
| **Volume** | 523.22(7) Å$^3$ | |
| ***Z*** | 6 | |
| **Density (calculated)** | 3.913 mg/m$^3$ | |
| **Absorption coefficient** | 10.706 mm$^{-1}$ | |
| **F(000)** | 587 | |
| **Crystal size** | 0.16 × 0.065 × 0.015 mm$^3$ | |
| **Theta range for data collection** | 2.915 to 49.189° | |
| **Index ranges** | -30<=h<=32, -7<=k<=7, -21<=l<=21 | |
| **Reflections collected** | 2889 | |
| **Independent reflections** | 2889 [R(int) = 0.0557] | |
| **Completeness to theta = 25.242°** | 99.20% | |
| **Absorption correction** | multi-scan | |
| **Max. and min. transmission** | 0.5504 and 0.7479 | |
| **Refinement method** | Full-matrix least-squares on F$^2$ | |
| **Data / restraints / parameters** | 2889 / 0 / 73 | |
| **Goodness-of-fit on F$^2$** | 1.14 | |
| **Final R indices [I>2sigma(I)]** | R1 = 0.0336, wR2 = 0.0656 | |

| | |
|---|---|
| **R indices (all data)** | R1 = 0.0257, wR2 = 0.0582 |
| **Largest diff. peak and hole** | 0.1592 and -0.1585 |

***Table S24. Atomic coordinates, occupancies, and equivalent isotropic displacement parameters (Å$^2$) for β-$Pb_{0.115}V_2O_5$: pbv2o5_4511b_0m_a. U(eq) is defined as one third of the trace of the orthogonalized $U^{ij}$ tensor.***

| Atom | x | y | z | Occupancy | Uiso |
|---|---|---|---|---|---|
| Pb(1) | 0.49623(2) | 0.5000 | 0.90370(4) | 0.172 | 0.00852(8) |
| V(1) | 0.71166(2) | 1.0000 | 0.08942(2) | 1 | 0.00366(4) |
| V(2) | 0.66209(2) | 1.0000 | 0.39973(2) | 1 | 0.003(4) |
| V(3) | 0.61706(2) | 0.5000 | 0.61967(2) | 1 | 0.005(4) |
| O(1) | 0.74252(8) | 0.5000 | 0.07407(11) | 1 | 0.006(14) |
| O(2) | 0.60033(8) | 1.0000 | 0.02631(13) | 1 | 0.009(16) |
| O(3) | 0.73586(7) | 1.0000 | 0.27626(11) | 1 | 0.005(13) |
| O(4) | 0.56180(8) | 1.0000 | 0.28023(13) | 1 | 0.010(18) |
| O(5) | 0.68548(8) | 0.5000 | 0.44526(12) | 1 | 0.006(14) |
| O(6) | 0.5000 | 0.5000 | 0.5000 | 1 | 0.007(2) |
| O(7) | 0.63434(7) | 1.0000 | 0.57860(11) | 1 | 0.005(13) |
| O(8) | 0.60735(8) | 0.5000 | 0.77424(12) | 1 | 0.007(14) |

***Table S25. Anisotropic displacement parameters (Å$^2$x 10$^3$) for β-$Pb_{0.115}V_2O_5$: pbv2o5_4511b_0m_a. The anisotropic displacement factor exponent takes the form: $-2\pi^2[ h^2 a^{*2}U^{11} + ... + 2 h k a^* b^* U^{12}]$***

| Atom | U11 | U22 | U33 | U23 | U13 | U12 |
|---|---|---|---|---|---|---|
| Pb(1) | 8(12) | 8(12) | 9(13) | 0 | 2(9) | 0 |
| V(1) | 5(7) | 3(7) | 4(7) | 0 | 2(5) | 0 |
| V(2) | 4(7) | 3(7) | 3(7) | 0 | 2(5) | 0 |
| V(3) | 7(8) | 3(7) | 5(7) | 0 | 3(6) | 0 |
| O(1) | 9(4) | 4(3) | 6(3) | 0 | 4(3) | 0 |
| O(2) | 5(3) | 13(4) | 7(4) | 0 | 2(3) | 0 |
| O(3) | 5(3) | 5(3) | 4(3) | 0 | 1(2) | 0 |
| O(4) | 6(4) | 18(5) | 6(3) | 0 | 1(3) | 0 |
| O(5) | 9(4) | 4(3) | 6(3) | 0 | 3(3) | 0 |
| O(6) | 4(4) | 6(5) | 8(5) | 0 | -0(4) | 0 |
| O(7) | 6(3) | 3(3) | 5(3) | 0 | 2(2) | 0 |
| O(8) | 9(4) | 8(3) | 5(3) | 0 | 3(3) | 0 |

***Table S26. Bond lengths [Å] and angles [°] for β-$Pb_{0.115}V_2O_5$: pbv2o5_4511b_0m_a.***

| Atom pairs | Bond Length [Å] | Atom pairs | Bond Length [Å] |
|---|---|---|---|
| V(1)—V(3) | 3.2001(4) | V(1)—V(1)#9 | 3.0481(4) |
| Pb(1)—O(2)[ii] | 2.4488(8) | V(2)—O(4) | 1.5996(12) |
| Pb(1)—O(2)#3 | 2.4488(8) | V(2)—O(5) | 1.8673(3) |
| Pb(1)—O(8) | 2.4681(12) | V(2)—O(5)#7 | 1.8673(3) |
| Pb(1)—O(4)#4 | 2.5195(9) | V(2)—O(3) | 1.9400(11) |
| Pb(1)—O(4)#5 | 2.5195(9) | V(2)—O(7) | 1.9795(11) |
| Pb(1)—O(2)#4 | 2.5791(9) | V(2)—O(5)#10 | 2.3302(12) |
| Pb(1)—O(2)#5 | 2.5791(9) | V(2)—V(3)#7 | 3.1045(3) |
| Pb(1)—Pb(1)#6 | 3.6112(3) | V(2)—V(3) | 3.1045(3) |
| Pb(1)—Pb(1)#7 | 3.6112(3) | V(3)—O(8) | 1.6073(11) |
| V(1)—O(2) | 1.6087(12) | V(3)—O(6) | 1.7916(3) |
| V(1)—O(3) | 1.7836(10) | V(3)—O(7)#6 | 1.8905(4) |
| V(1)—O(1) | 1.8861(4) | V(3)—O(7) | 1.8905(4) |
| V(1)—O(1)#7 | 1.8861(4) | V(3)—O(3)#10 | 2.1425(11) |
| V(1)—O(1)#8 | 1.9910(11) | V(3)—O(5) | 2.3252(12) |
| V(1)—V(1)#8 | 3.0481(4) | V(1)—V(1)#9 | 3.0481(4) |
| V(2)—O(5)#7 | 1.8673(3) | V(2)—O(4) | 1.5996(12) |
| V(2)—O(3) | 1.9400(11) | V(2)—O(5) | 1.8673(3) |

| Atoms | Bond Angle [°] | Atoms | Bond Angle [°] |
|---|---|---|---|
| Pb(1)#1—Pb(1)—O(2)#2 | 71.54(3) | O(1)#7—V(1)—O(1)#8 | 76.37(3) |
| Pb(1)#1—Pb(1)—O(2)#3 | 71.54(3) | O(2)—V(1)—V(1)#8 | 109.76(4) |
| O(2)#2—Pb(1)—O(2)#3 | 95.01(4) | O(3)—V(1)—V(1)#8 | 129.35(2) |
| Pb(1)#1—Pb(1)—O(8) | 136.15(4) | O(1)—V(1)—V(1)#8 | 39.41(3) |
| O(2)#2—Pb(1)—O(8) | 79.34(3) | O(1)#7—V(1)—V(1)#8 | 111.19(3) |
| O(2)#3—Pb(1)—O(8) | 79.34(3) | O(1)#8—V(1)—V(1)#8 | 36.968(8) |
| Pb(1)#1—Pb(1)—O(4)#4 | 129.13(3) | O(2)—V(1)—V(1)#9 | 109.76(4) |
| O(2)#2—Pb(1)—O(4)#4 | 154.88(4) | O(3)—V(1)—V(1)#9 | 129.35(2) |
| O(2)#3—Pb(1)—O(4)#4 | 81.29(3) | O(1)—V(1)—V(1)#9 | 111.19(3) |
| O(8)—Pb(1)—O(4)#4 | 75.55(3) | O(1)#7—V(1)—V(1)#9 | 39.41(3) |
| Pb(1)#1—Pb(1)—O(4)#5 | 129.13(3) | O(1)#8—V(1)—V(1)#9 | 36.968(8) |
| O(2)#2—Pb(1)—O(4)#5 | 81.29(3) | V(1)#8—V(1)—V(1)#9 | 72.651(11) |
| O(2)#3—Pb(1)—O(4)#5 | 154.88(4) | O(4)—V(2)—O(5) | 103.89(3) |
| O(8)—Pb(1)—O(4)#5 | 75.55(3) | O(4)—V(2)—O(5)#7 | 103.89(3) |
| O(4)#4—Pb(1)—O(4)#5 | 91.56(4) | O(5)—V(2)—O(5)#7 | 150.46(7) |
| Pb(1)#1—Pb(1)—O(2)#4 | 64.24(3) | O(4)—V(2)—O(3) | 98.19(6) |
| O(2)#2—Pb(1)—O(2)#4 | 135.78(2) | O(5)—V(2)—O(3) | 92.91(4) |
| O(2)#3—Pb(1)—O(2)#4 | 71.59(4) | O(5)#7—V(2)—O(3) | 92.91(4) |
| O(8)—Pb(1)—O(2)#4 | 134.90(2) | O(4)—V(2)—O(7) | 103.43(6) |
| O(4)#4—Pb(1)—O(2)#4 | 66.70(4) | O(5)—V(2)—O(7) | 81.99(4) |
| O(4)#5—Pb(1)—O(2)#4 | 127.43(4) | O(5)#7—V(2)—O(7) | 81.99(4) |
| Pb(1)#1—Pb(1)—O(2)#5 | 64.24(3) | O(3)—V(2)—O(7) | 158.38(4) |
| O(2)#2—Pb(1)—O(2)#5 | 71.59(4) | O(1)#7—V(1)—O(1)#8 | 76.37(3) |
| O(2)#3—Pb(1)—O(2)#5 | 135.78(2) | O(2)—V(1)—V(1)#8 | 109.76(4) |
| O(8)—Pb(1)—O(2)#5 | 134.90(2) | O(3)—V(1)—V(1)#8 | 129.35(2) |
| O(4)#4—Pb(1)—O(2)#5 | 127.43(4) | O(1)—V(1)—V(1)#8 | 39.41(3) |
| O(4)#5—Pb(1)—O(2)#5 | 66.70(4) | O(1)#7—V(1)—V(1)#8 | 111.19(3) |
| O(2)#4—Pb(1)—O(2)#5 | 88.87(4) | O(1)#8—V(1)—V(1)#8 | 36.968(8) |

| | | | |
|---|---|---|---|
| Pb(1)#1—Pb(1)—Pb(1)#6 | 90.000 | O(2)—V(1)—V(1)#9 | 109.76(4) |
| O(2)#2—Pb(1)—Pb(1)#6 | 137.51(2) | O(3)—V(1)—V(1)#9 | 129.35(2) |
| O(2)#3—Pb(1)—Pb(1)#6 | 42.49(2) | O(1)—V(1)—V(1)#9 | 111.19(3) |
| O(8)—Pb(1)—Pb(1)#6 | 89.999(1) | O(1)#7—V(1)—V(1)#9 | 39.41(3) |
| O(4)#4—Pb(1)—Pb(1)#6 | 44.22(2) | O(1)#8—V(1)—V(1)#9 | 36.968(8) |
| O(4)#5—Pb(1)—Pb(1)#6 | 135.78(2) | V(1)#8—V(1)—V(1)#9 | 72.651(11) |
| O(2)#4—Pb(1)—Pb(1)#6 | 45.57(2) | O(4)—V(2)—O(5) | 103.89(3) |
| O(2)#5—Pb(1)—Pb(1)#6 | 134.44(2) | O(4)—V(2)—O(5)#7 | 103.89(3) |
| Pb(1)#1—Pb(1)—Pb(1)#7 | 90.000 | O(5)—V(2)—O(5)#7 | 150.46(7) |
| O(2)#2—Pb(1)—Pb(1)#7 | 42.49(2) | O(4)—V(2)—O(3) | 98.19(6) |

**Symmetry transformations used to generate equivalent atoms:**
(i) 1-x, 1-y, 2-z; (ii) x, y, 1+z; (iii) x, -1+y, 1+z; (iv) 1-x, 1-y, 1-z;
(v) 1-x, 2-y, 1-z; (vi) x, -1+y, z; (vii) x, 1+y, z; (viii) 1.5-x, 1.5-y, -z;
(ix) 1.5-x, 2.5-y, -z; (x) 1.5-x, 1.5-y, 1-z; (xi) x, y, -1+z; (xii) x, 1+y, -1+z.

**Table S27. Interatomic distances (Å) and bond valence sums (BVS) for β-$Pb_x$/β'-$Cu_yV_2O_5$, β-$Pb_{0.33}V_2O_5$, and β'-$Cu_{0.65}V_2O_5$**

| Bond | distance (Å) | Bond | distance (Å) | Bond | distance (Å) | Bond | distance (Å) | Bond | distance (Å) |
|---|---|---|---|---|---|---|---|---|---|
| **β-$Pb_{0.01}$/β'-$Cu_{0.47}V_2O_5$** | | **β-$Pb_{0.08}$/β'-$Cu_{0.33}V_2O_5$** | | **β-$Pb_{0.11}$/β'-$Cu_{0.41}V_2O_5$** | | **β-$Pb_{0.33}V_2O_5$** | | **β'-$Cu_{0.65}V_2O_5$** | |
| V1—O1 (x 2) | 1.898 | V1—O1 (× 2) | 1.896 | V1—O1 (x 2) | 1.896 | V1—O2 (x 2) | 1.888 | V1—O1 | 1.656 |
| V1—O1 | 1.987 | V1—O1 | 1.990 | V1—O1 | 1.987 | V1—O2 | 2.297 | V1—O2 | 1.817 |
| V1—O2 | 1.622 | V1—O2 | 1.616 | V1—O2 | 1.632 | V1—O3 | 1.988 | V1—O3 | 2.175 |
| V1—O3 | 1.778 | V1—O3 | 1.780 | V1—O3 | 1.788 | V1—O4 | 1.612 | V1—O4 | 2.242 |
| V1—O8 | 2.801 | V1—O8 | 2.733 | V1—O8 | 2.713 | V1—O5 | 1.924 | V1—O6(x2) | 1.885 |
| **BVS** | **4.679** | **BVS** | **4.711** | **BVS** | **4.634** | **BVS** | **4.612** | **BVS** | **4.491** |
| | | | | | | | | | |
| V2—O3 | 1.950 | V2—O3 | 1.949 | V2—O3 | 1.928 | V2—O1 | 1.805 | V2—O3 | 1.934 |
| V2—O4 | 1.612 | V2—O4 | 1.612 | V2—O4 | 1.617 | V2—O2 | 2.266 | V2—O4 | 2.216 |
| V2—O5 (x 2) | 1.876 | V2—O5 (x 2) | 1.877 | V2—O5 (x 2) | 1.881 | V2—O3 (x 2) | 1.898 | V2—O4(x2) | 1.882 |
| V2—O5 | 2.251 | V2—O5 | 2.272 | V2—O5 | 2.247 | V2—O5 | 2.163 | V2—O5 | 1.621 |
| V2—O7 | 1.985 | V2—O7 | 1.984 | V2—O7 | 1.985 | V2—O6 | 1.612 | V2—O6 | 1.988 |
| **BVS** | **4.655** | **BVS** | **4.643** | **BVS** | **4.654** | **BVS** | **4.637** | **BVS** | **4.644** |
| | | | | | | | | | |
| V3—O3 | 2.171 | V3—O3 | 2.167 | V3—O3 | 2.170 | V3—O5 | 1.807 | V3—O1 | 2.827 |
| V3—O5 | 2.280 | V3—O5 | 2.286 | V3—O5 | 2.245 | V3—O6 | 2.601 | V3—O3 | 1.785 |
| V3—O6 | 1.811 | V3—O6 | 1.809 | V3—O6 | 1.812 | V3—O7 | 1.983 | V3—O7 | 1.639 |
| V3—O7 (x 2) | 1.888 | V3—O7 (x 2) | 1.890 | V3—O7 (x 2) | 1.887 | V3—O7 (x 2) | 1.895 | V3—O8(x2) | 1.979 |
| V3—O8 | 1.640 | V3—O8 | 1.635 | V3—O8 | 1.650 | V3—O8 | 1.600 | V3—O8 | 1.899 |
| **BVS** | **4.529** | **BVS** | **4.544** | **BVS** | **4.523** | **BVS** | **4.762** | **BVS** | **4.592** |

BV = $e^{(r0-r)/b}$ with the following parameters: b = 0.37, $r_0$(V—O) = 1.784Å

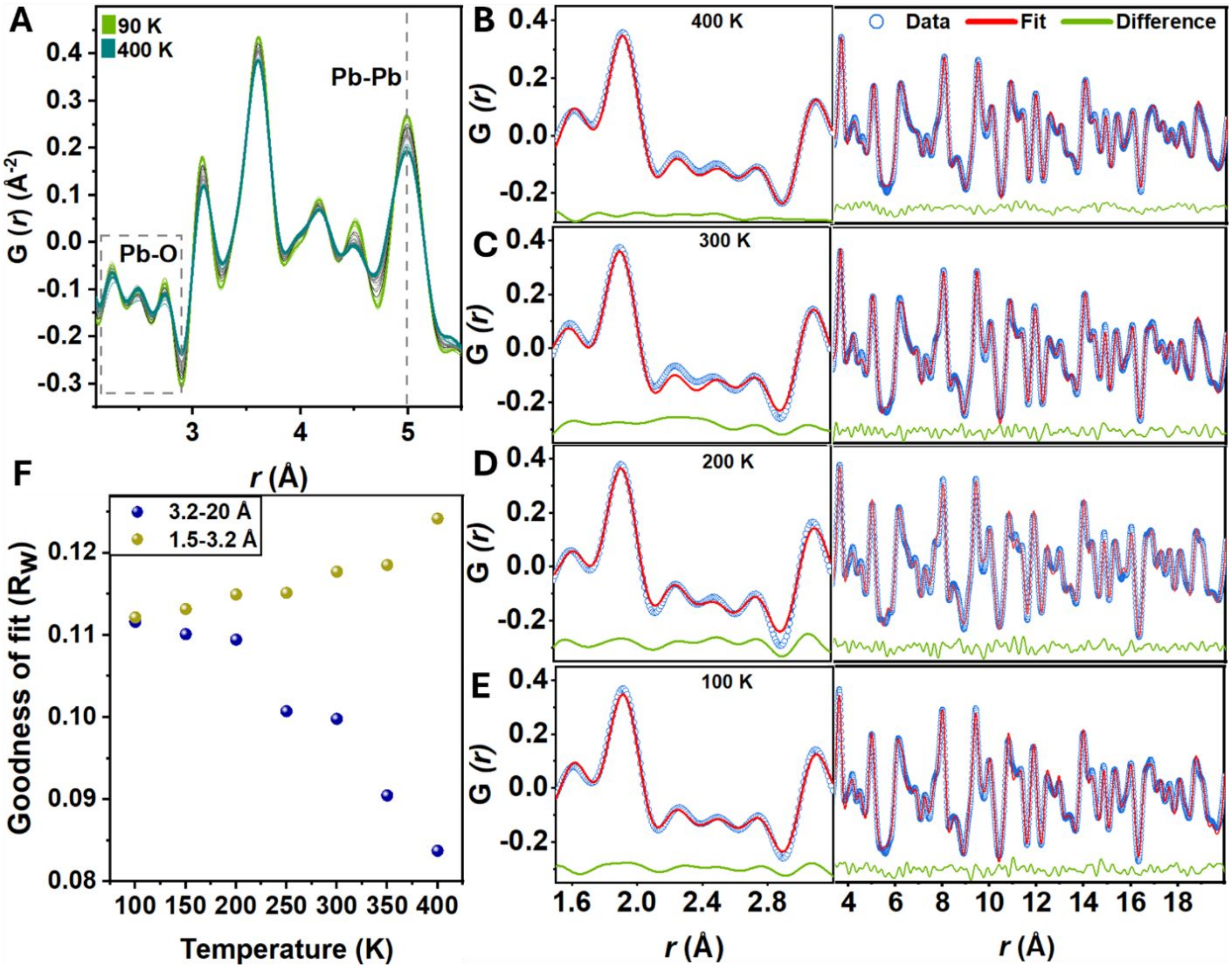


***Figure S6. X-ray pair distribution function analysis of β-$Pb_x$/β'-$Cu_yV_2O_5$.*** (**A**) Temperature dependence of synchrotron X-PDF data of β-$Pb_{0.05}$/β'-$Cu_{0.33}V_2O_5$ in the 90 K(green)– 400 K(cyan) range in 5 K increments. The dashed lines centered at ∼2.5−2.8 Å and 5.0 Å, corresponding to Pb−O and Pb−Pb correlations, respectively, exhibit pronounced asymmetry with increasing temperature. Fits of XPDF data to the average monoclinic C2/m structure model at (**B**) 400 K, (**C**) 300 K, (**D**) 200 K and (**E**) 100 K. The experimental PDF (blue open circles) is shown with the calculated C2/m model (red line). The difference between the observed and calculated data is indicated by the green residual curve, offset for clarity. (**F**) Temperature-dependent goodness of fit ($R_w$) value obtained from the monoclinic C2/m of the X-PDF data for low r peaks (r = 1.5− 3.2 Å) and high r peaks (r = 3.2−20 Å).

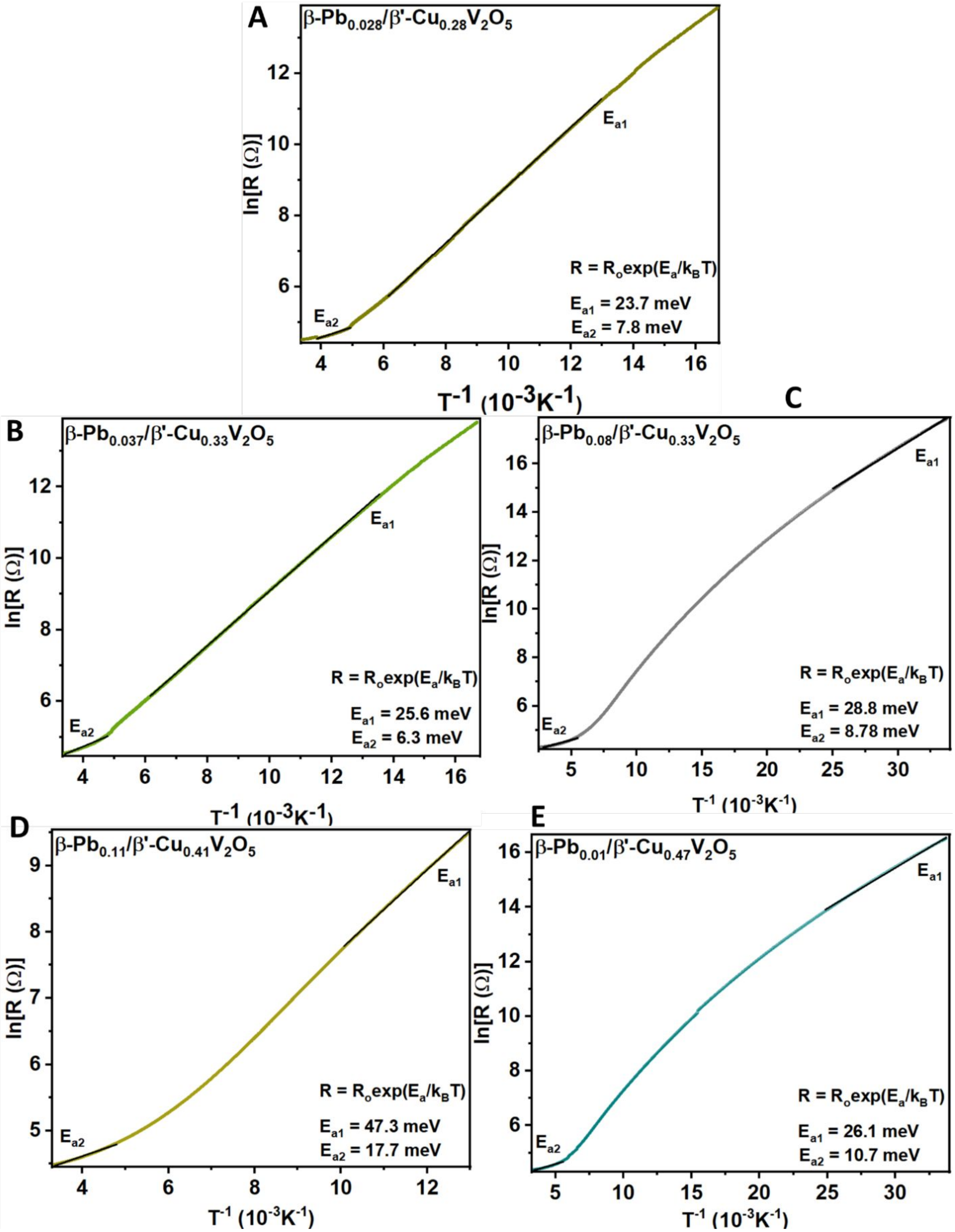


***Figure S7*. *Probing the activation energy of $\beta$-$Pb_x$/$\beta'$-$Cu_yV_2O_5$*.** Exponential fit with thermal activation energies for single crystals of **(A)** $\beta$-$Pb_{0.028}$/$\beta'$-$Cu_{0.28}V_2O_5$, and **(B)** $\beta$-$Pb_{0.037}$/$\beta'$-$Cu_{0.33}V_2O_5$, **(C)** $\beta$-$Pb_{0.08}$/$\beta'$-$Cu_{0.33}V_2O_5$, **(D)** $\beta$-$Pb_{0.11}$/$\beta'$-$Cu_{0.41}V_2O_5$, and **(E)** $\beta$-$Pb_{0.01}$/$\beta'$-$Cu_{0.47}V_2O_5$. The activation energy increases with increasing Pb stoichiometry from 23.7 to 7.8 meV for $Pb_{0.028}$/$\beta'$-$Cu_{0.28}V_2O_5$ and 47.3 to 17.7 meV for $\beta$-$Pb_{0.11}$/$\beta'$-$Cu_{0.41}V_2O_5$. We define $\Delta E_a$ as the difference in activation energies measured in temperature ranges 40—99K and 207—299K. For compound **e**, $\beta$-$Pb_{0.11}$/$\beta'$-$Cu_{0.41}V_2O_5$ (**Figure S5D**), the activation energy decreases from 47.3 to

17.7 meV with an increase in temperature. We define $\Delta E_a$ as the difference in activation energies measured in temperature ranges 40—99K and 207—299K (**Table 1**). $\Delta E_a$ increases with increasing Pb-ion stoichiometry (**Figure S5A-E**), which suggests that the p-block cation stabilizes a more insulating charge-localized electronic phase.

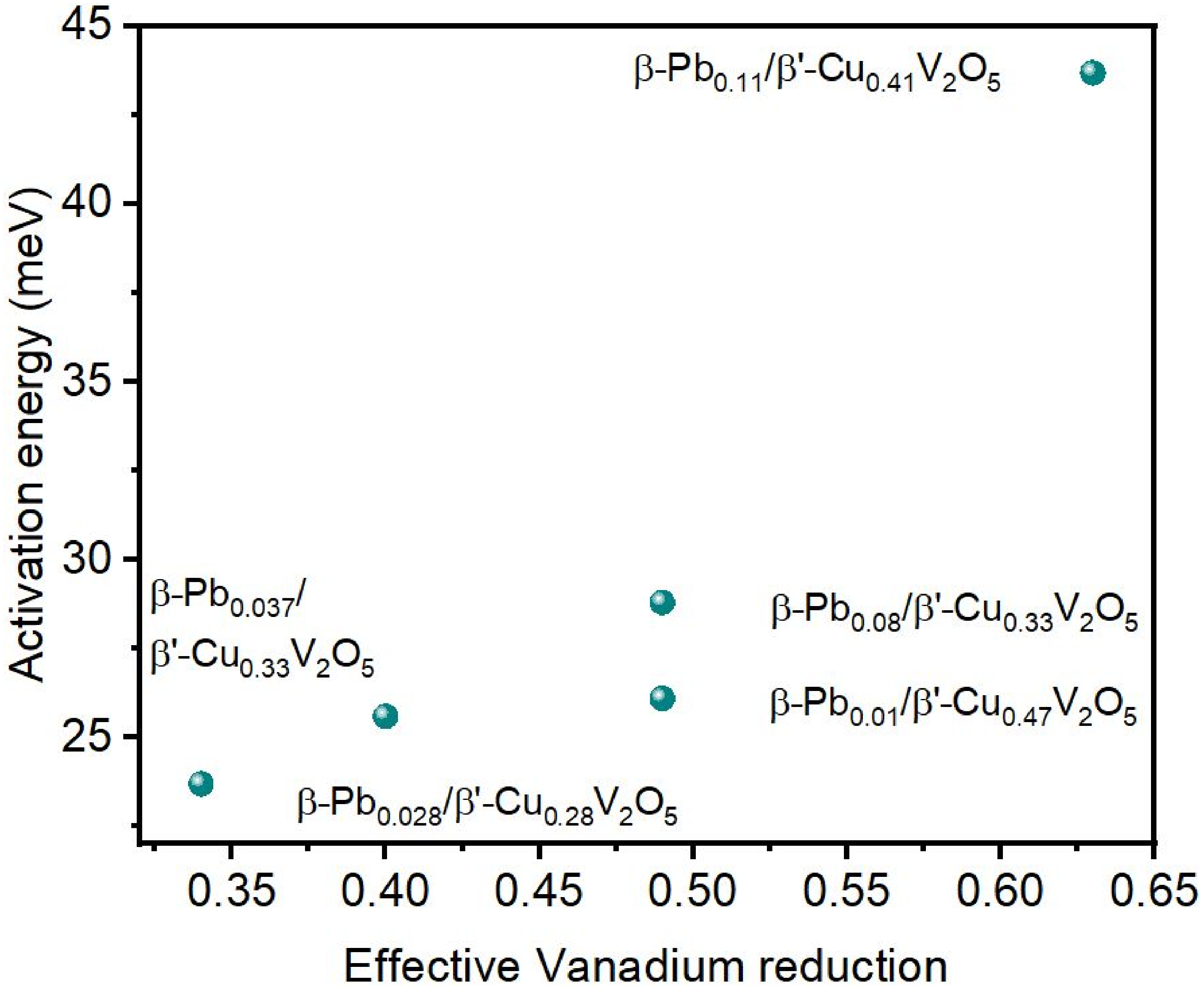


***Figure S8.*** Activation energy ($E_a$) as a function of effective vanadium reduction plotted for $\beta$-$Pb_{0.028}/\beta'$-$Cu_{0.28}V_2O_5$, $\beta$-$Pb_{0.037}/\beta'$-$Cu_{0.33}V_2O_5$, $\beta$-$Pb_{0.08}/\beta'$-$Cu_{0.33}V_2O_5$, $\beta$-$Pb_{0.11}/\beta'$-$Cu_{0.41}V_2O_5$, and $\beta$-$Pb_{0.01}/\beta'$-$Cu_{0.47}V_2O_5$ crystals. A correlation is observed between increasing formal vanadium reduction and higher activation energies for conduction. This trend reflects enhanced charge localization with increasing Pb insertion, which stabilizes the insulating state, and thereby increases the energy barrier for carrier hopping between vanadium sites. Activation energies are extracted from the low-temperature regime, where intrinsic transport behavior dominates; at higher temperatures, defect-dependent scattering mechanisms are predominant.

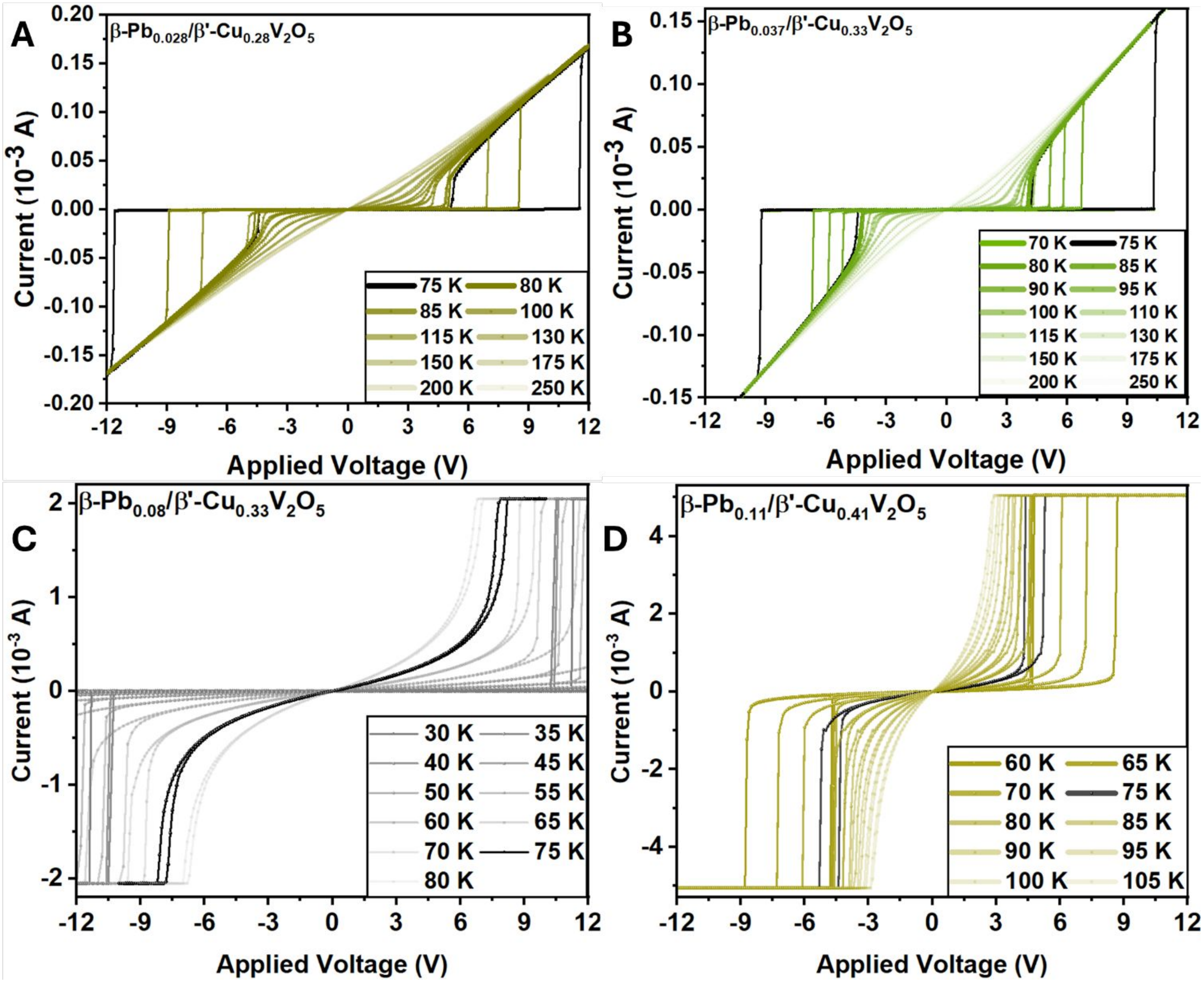


***Figure S9**. **Electronic instabilities in single crystals of $\beta$-Pb$_x$/$\beta$'-Cu$_y$V$_2$O$_5$.*** Current versus voltage characteristics measured for a single crystal of **(A)** $\beta$-Pb$_{0.028}$/$\beta$'-Cu$_{0.28}$V$_2$O$_5$ between 75 and 250K; **(B)** $\beta$-Pb$_{0.037}$/$\beta$'-Cu$_{0.33}$V$_2$O$_5$ between 70 and 250K, **(C)** $\beta$-Pb$_{0.08}$/$\beta$'-Cu$_{0.33}$V$_2$O$_5$ between 30 and 80K; and **(D)** $\beta$-Pb$_{0.11}$/$\beta$'-Cu$_{0.41}$V$_2$O$_5$ between 55 and 105K. With increasing temperature, the threshold voltage for the discontinuous jump decreases monotonically across all compounds until 70K for $\beta$-Pb$_{0.08}$/$\beta$'-Cu$_{0.33}$V$_2$O$_5$, 90K $\beta$-Pb$_{0.11}$/$\beta$'-Cu$_{0.41}$V$_2$O$_5$, 130K for $\beta$-Pb$_{0.028}$/$\beta$'-Cu$_{0.28}$V$_2$O$_5$, and 115K for $\beta$-Pb$_{0.037}$/$\beta$'-Cu$_{0.33}$V$_2$O$_5$, where a pronounced jump is no longer observed. The abrupt jump in current corresponds to strongly nonlinear dynamical response.

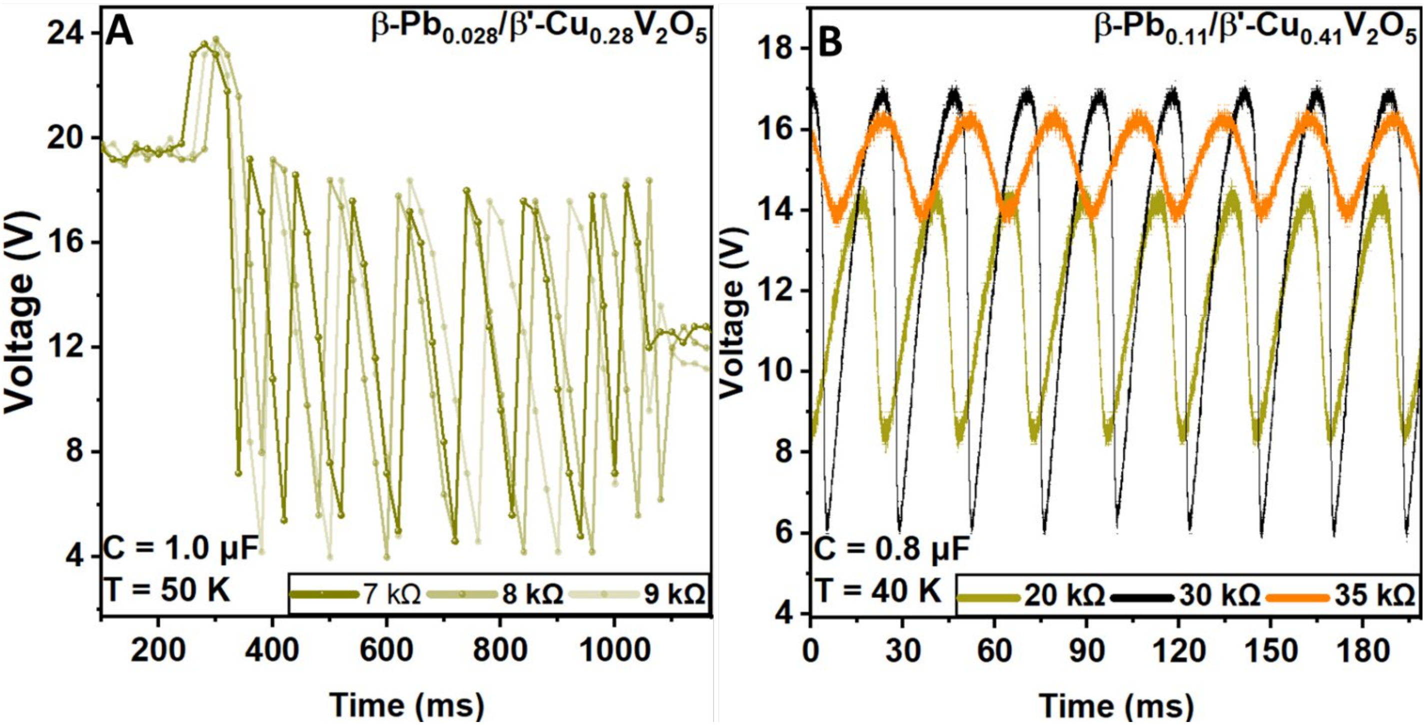


***Figure S10. Characterization of Single Crystal Oscillators of β-$Pb_{0.028}$/β'-$Cu_{0.28}V_2O_5$ and β-$Pb_{0.11}$/β'-$Cu_{0.41}V_2O_5$** .* Oscillations exhibited by a single crystal of **(A)** β-$Pb_{0.028}$/β'-$Cu_{0.28}V_2O_5$ at T = 50K, V = 40 V, $R_S$ = 7, 8, 9 kΩ, C = 1000 nF; and **(B)** β-$Pb_{0.11}$/β'-$Cu_{0.41}V_2O_5$ at T = 40K, V = 25 V, $R_S$ = 20, 30, 35 kΩ, C = 800 nF.

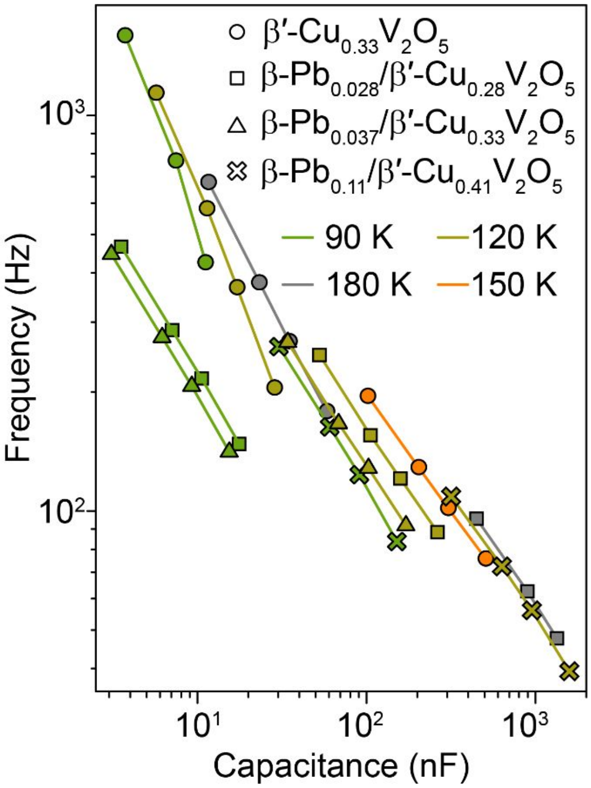


***Figure S11. Compact Model of Electrothermal Single Crystal Oscillators***. Frequency plotted versus total circuit capacitance predicted in an electrothermal oscillator simulation for β'-$Cu_xV_2O_5$, β-$Pb_{0.028}$/β'-$Cu_{0.28}V_2O_5$, β-$Pb_{0.037}$/β'-$Cu_{0.33}V_2O_5$, and β-$Pb_{0.11}$/β'-$Cu_{0.41}V_2O_5$ crystals with dimensions of 500 × 100 × 50 μm at varying temperatures and for the experimental results in (**Figures 2A, 3D, S10**).

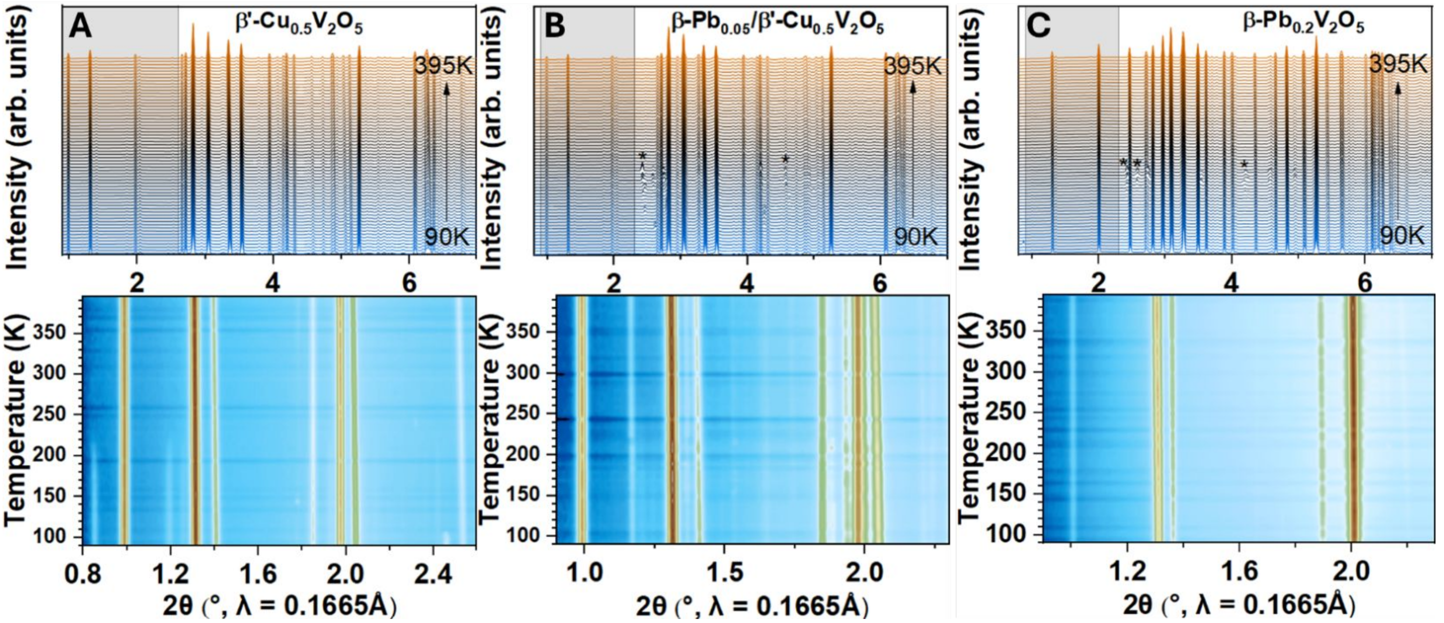


***Figure S12. Structural evolution of end-member $M_xV_2O_5$ (M =Cu) and co-intercalated $M_XM'_yV_2O_5$.*** Synchrotron X-ray diffraction contour color plots as a function of temperature (90-395K) collected using a ramp rate of 1 K for(**A**) β'-$Cu_{0.50}V_2O_5$, (**B**) β-$Pb_{0.05}$/β'-$Cu_{0.5}V_2O_5$, and (**C**) β-$Pb_{0.2}V_2O_5$, powders.

**Table S28.** Lattice parameters, standard deviations, unit cell volumes, and statistics of the sequential refinement of β-$Pb_{0.2}V_2O_5$

| Temp. (K) | $R_{wp}$ (%) | *a* (Å) | std. dev. (Å) | *b* (Å) | std. dev. (Å) | *c* (Å) | std. dev. (Å) | β (°) | std. dev. (°) | Vol. (Å³) | std. dev. (Å³) |
|---|---|---|---|---|---|---|---|---|---|---|---|
| 90 | 4.23 | 15.44461 | 6.56E-04 | 3.626864 | 3.15E-05 | 10.07459 | 3.27E-04 | 109.2828 | 1.17E-03 | 532.6737 | 6.99E-03 |
| 95 | 4.15 | 15.44513 | 6.45E-04 | 3.626818 | 3.10E-05 | 10.07467 | 3.22E-04 | 109.2833 | 1.15E-03 | 532.6879 | 6.88E-03 |
| 100 | 4.35 | 15.44579 | 6.76E-04 | 3.626877 | 3.27E-05 | 10.0746 | 3.38E-04 | 109.2814 | 1.21E-03 | 532.7218 | 7.23E-03 |
| 105 | 5.21 | 15.44706 | 8.11E-04 | 3.626842 | 3.93E-05 | 10.07486 | 4.05E-04 | 109.2824 | 1.45E-03 | 532.7711 | 8.67E-03 |
| 110 | 4.30 | 15.44789 | 6.69E-04 | 3.626801 | 3.24E-05 | 10.07507 | 3.34E-04 | 109.2819 | 1.19E-03 | 532.8064 | 7.14E-03 |
| 115 | 4.26 | 15.44865 | 6.64E-04 | 3.626785 | 3.21E-05 | 10.07525 | 3.31E-04 | 109.2813 | 1.18E-03 | 532.8415 | 7.09E-03 |
| 120 | 4.27 | 15.44954 | 6.68E-04 | 3.626744 | 3.23E-05 | 10.07561 | 3.33E-04 | 109.2807 | 1.19E-03 | 532.8875 | 7.13E-03 |
| 125 | 4.25 | 15.4506 | 6.66E-04 | 3.626732 | 3.22E-05 | 10.07577 | 3.33E-04 | 109.2802 | 1.19E-03 | 532.9323 | 7.12E-03 |
| 130 | 4.30 | 15.4518 | 6.73E-04 | 3.626679 | 3.25E-05 | 10.07612 | 3.36E-04 | 109.281 | 1.20E-03 | 532.9815 | 7.19E-03 |
| 135 | 4.22 | 15.45258 | 6.62E-04 | 3.626643 | 3.20E-05 | 10.07634 | 3.31E-04 | 109.2778 | 1.18E-03 | 533.0253 | 7.09E-03 |
| 140 | 4.19 | 15.45386 | 6.54E-04 | 3.626597 | 3.17E-05 | 10.07661 | 3.27E-04 | 109.278 | 1.17E-03 | 533.076 | 7.01E-03 |
| 145 | 4.15 | 15.45486 | 6.49E-04 | 3.626524 | 3.13E-05 | 10.07694 | 3.24E-04 | 109.2783 | 1.16E-03 | 533.1164 | 6.96E-03 |
| 150 | 4.16 | 15.45613 | 6.52E-04 | 3.626456 | 3.14E-05 | 10.07736 | 3.26E-04 | 109.2773 | 1.16E-03 | 533.176 | 6.99E-03 |
| 155 | 4.25 | 15.45715 | 6.68E-04 | 3.626419 | 3.19E-05 | 10.07769 | 3.33E-04 | 109.2762 | 1.19E-03 | 533.2269 | 7.14E-03 |
| 160 | 4.12 | 15.45865 | 6.46E-04 | 3.626398 | 3.11E-05 | 10.07812 | 3.22E-04 | 109.2791 | 1.15E-03 | 533.2888 | 6.93E-03 |
| 165 | 4.18 | 15.45937 | 6.59E-04 | 3.626378 | 3.16E-05 | 10.07837 | 3.29E-04 | 109.2771 | 1.17E-03 | 533.3303 | 7.05E-03 |
| 170 | 4.16 | 15.46054 | 6.54E-04 | 3.626341 | 3.16E-05 | 10.07864 | 3.27E-04 | 109.2746 | 1.17E-03 | 533.3878 | 7.01E-03 |
| 175 | 4.59 | 15.46175 | 7.28E-04 | 3.626289 | 3.47E-05 | 10.07906 | 3.63E-04 | 109.2742 | 1.30E-03 | 533.445 | 7.80E-03 |
| 180 | 4.59 | 15.46274 | 7.27E-04 | 3.626275 | 3.50E-05 | 10.07931 | 3.63E-04 | 109.275 | 1.30E-03 | 533.4883 | 7.81E-03 |
| 185 | 5.32 | 15.46406 | 8.48E-04 | 3.626143 | 4.06E-05 | 10.07974 | 4.24E-04 | 109.2744 | 1.51E-03 | 533.5388 | 9.09E-03 |
| 190 | 4.59 | 15.46527 | 7.31E-04 | 3.626106 | 3.50E-05 | 10.08017 | 3.65E-04 | 109.2727 | 1.30E-03 | 533.6036 | 7.84E-03 |
| 195 | 6.07 | 15.46671 | 9.79E-04 | 3.626116 | 4.67E-05 | 10.08039 | 4.89E-04 | 109.2746 | 1.74E-03 | 533.6602 | 1.05E-02 |
| 200 | 7.64 | 15.46808 | 1.26E-03 | 3.626073 | 6.01E-05 | 10.08096 | 6.29E-04 | 109.2745 | 2.24E-03 | 533.732 | 1.35E-02 |
| 205 | 9.55 | 15.46995 | 1.59E-03 | 3.625949 | 7.51E-05 | 10.08145 | 7.95E-04 | 109.2763 | 2.83E-03 | 533.7977 | 1.70E-02 |
| 210 | 9.51 | 15.47073 | 1.62E-03 | 3.625985 | 7.64E-05 | 10.08179 | 8.09E-04 | 109.2763 | 2.88E-03 | 533.848 | 1.73E-02 |
| 215 | 11.87 | 15.47214 | 2.04E-03 | 3.625918 | 9.72E-05 | 10.082 | 1.02E-03 | 109.2741 | 3.64E-03 | 533.9052 | 2.19E-02 |
| 220 | 4.27 | 15.47248 | 6.76E-04 | 3.625971 | 3.22E-05 | 10.08271 | 3.37E-04 | 109.2721 | 1.20E-03 | 533.9694 | 7.23E-03 |
| 225 | 4.27 | 15.47403 | 6.78E-04 | 3.625925 | 3.24E-05 | 10.08319 | 3.38E-04 | 109.2695 | 1.20E-03 | 534.0499 | 7.26E-03 |
| 230 | 4.16 | 15.47502 | 6.59E-04 | 3.625871 | 3.15E-05 | 10.0837 | 3.29E-04 | 109.2698 | 1.17E-03 | 534.1019 | 7.07E-03 |
| 235 | 4.21 | 15.47647 | 6.68E-04 | 3.625819 | 3.18E-05 | 10.08421 | 3.33E-04 | 109.2698 | 1.19E-03 | 534.1716 | 7.16E-03 |
| 240 | 4.19 | 15.47772 | 6.64E-04 | 3.625783 | 3.17E-05 | 10.08468 | 3.31E-04 | 109.2698 | 1.18E-03 | 534.2338 | 7.13E-03 |
| 245 | 4.23 | 15.47899 | 6.70E-04 | 3.625747 | 3.20E-05 | 10.08508 | 3.34E-04 | 109.2693 | 1.19E-03 | 534.2951 | 7.19E-03 |
| 250 | 4.21 | 15.48024 | 6.67E-04 | 3.625767 | 3.19E-05 | 10.08545 | 3.33E-04 | 109.2687 | 1.19E-03 | 534.3625 | 7.16E-03 |
| 255 | 4.23 | 15.48136 | 6.72E-04 | 3.625792 | 3.22E-05 | 10.08598 | 3.35E-04 | 109.2686 | 1.19E-03 | 534.4335 | 7.21E-03 |
| 260 | 4.24 | 15.48274 | 6.74E-04 | 3.625711 | 3.20E-05 | 10.08644 | 3.36E-04 | 109.2685 | 1.20E-03 | 534.4941 | 7.22E-03 |
| 265 | 4.30 | 15.48402 | 6.84E-04 | 3.625652 | 3.26E-05 | 10.08694 | 3.41E-04 | 109.269 | 1.22E-03 | 534.5547 | 7.34E-03 |
| 270 | 4.33 | 15.485 | 6.89E-04 | 3.625683 | 3.28E-05 | 10.08732 | 3.43E-04 | 109.2679 | 1.22E-03 | 534.6165 | 7.39E-03 |

| | | | | | | | | | | | |
|---|---|---|---|---|---|---|---|---|---|---|---|
| 275 | 4.42 | 15.48643 | 7.05E-04 | 3.625687 | 3.35E-05 | 10.08773 | 3.52E-04 | 109.269 | 1.25E-03 | 534.6846 | 7.56E-03 |
| 280 | 4.43 | 15.4878 | 7.06E-04 | 3.625649 | 3.35E-05 | 10.08833 | 3.52E-04 | 109.2686 | 1.25E-03 | 534.7593 | 7.56E-03 |
| 285 | 4.48 | 15.48922 | 7.17E-04 | 3.625635 | 3.40E-05 | 10.08885 | 3.57E-04 | 109.2682 | 1.27E-03 | 534.8354 | 7.68E-03 |
| 290 | 4.44 | 15.49053 | 7.11E-04 | 3.625596 | 3.36E-05 | 10.08926 | 3.55E-04 | 109.2672 | 1.26E-03 | 534.9 | 7.61E-03 |
| 295 | 4.50 | 15.49202 | 7.22E-04 | 3.625598 | 3.42E-05 | 10.08992 | 3.60E-04 | 109.2673 | 1.28E-03 | 534.986 | 7.72E-03 |
| 300 | 4.45 | 15.49337 | 7.12E-04 | 3.625576 | 3.39E-05 | 10.09041 | 3.55E-04 | 109.2678 | 1.26E-03 | 535.0538 | 7.63E-03 |
| 310 | 4.72 | 15.49538 | 7.60E-04 | 3.625496 | 3.57E-05 | 10.09105 | 3.78E-04 | 109.268 | 1.34E-03 | 535.145 | 8.10E-03 |
| 315 | 4.59 | 15.49689 | 7.41E-04 | 3.625463 | 3.49E-05 | 10.09153 | 3.69E-04 | 109.2664 | 1.31E-03 | 535.2232 | 7.91E-03 |
| 320 | 4.55 | 15.49844 | 7.34E-04 | 3.625419 | 3.46E-05 | 10.09206 | 3.66E-04 | 109.2664 | 1.30E-03 | 535.2977 | 7.85E-03 |
| 325 | 4.56 | 15.49983 | 7.37E-04 | 3.625395 | 3.48E-05 | 10.09259 | 3.68E-04 | 109.2664 | 1.31E-03 | 535.3706 | 7.88E-03 |
| 330 | 4.59 | 15.50114 | 7.42E-04 | 3.625397 | 3.51E-05 | 10.09297 | 3.70E-04 | 109.2667 | 1.31E-03 | 535.4352 | 7.93E-03 |
| 335 | 4.60 | 15.50233 | 7.44E-04 | 3.625376 | 3.52E-05 | 10.09354 | 3.71E-04 | 109.266 | 1.32E-03 | 535.5059 | 7.95E-03 |
| 340 | 4.63 | 15.50377 | 7.48E-04 | 3.625334 | 3.54E-05 | 10.09396 | 3.73E-04 | 109.2654 | 1.32E-03 | 535.5734 | 8.00E-03 |
| 345 | 4.66 | 15.50528 | 7.55E-04 | 3.625312 | 3.57E-05 | 10.09446 | 3.76E-04 | 109.2654 | 1.34E-03 | 535.6488 | 8.07E-03 |
| 350 | 4.67 | 15.50673 | 7.56E-04 | 3.625277 | 3.57E-05 | 10.09486 | 3.77E-04 | 109.2649 | 1.34E-03 | 535.7164 | 8.08E-03 |
| 355 | 4.74 | 15.50826 | 7.66E-04 | 3.625268 | 3.62E-05 | 10.09544 | 3.82E-04 | 109.2649 | 1.36E-03 | 535.7988 | 8.19E-03 |
| 360 | 4.79 | 15.50966 | 7.76E-04 | 3.625248 | 3.67E-05 | 10.09606 | 3.87E-04 | 109.2655 | 1.37E-03 | 535.8755 | 8.29E-03 |
| 365 | 4.84 | 15.51109 | 7.85E-04 | 3.625228 | 3.71E-05 | 10.09669 | 3.91E-04 | 109.2653 | 1.39E-03 | 535.9558 | 8.38E-03 |
| 370 | 4.99 | 15.51247 | 8.09E-04 | 3.625226 | 3.83E-05 | 10.09717 | 4.03E-04 | 109.2656 | 1.43E-03 | 536.0279 | 8.63E-03 |
| 375 | 5.01 | 15.51395 | 8.14E-04 | 3.625159 | 3.83E-05 | 10.09774 | 4.06E-04 | 109.2653 | 1.44E-03 | 536.1005 | 8.67E-03 |
| 380 | 5.02 | 15.51537 | 8.16E-04 | 3.625117 | 3.84E-05 | 10.09825 | 4.07E-04 | 109.2651 | 1.44E-03 | 536.1708 | 8.70E-03 |
| 385 | 5.12 | 15.51658 | 8.34E-04 | 3.625086 | 3.92E-05 | 10.09881 | 4.16E-04 | 109.2646 | 1.47E-03 | 536.2395 | 8.89E-03 |
| 390 | 5.25 | 15.51788 | 8.57E-04 | 3.625087 | 4.02E-05 | 10.0994 | 4.26E-04 | 109.2652 | 1.51E-03 | 536.3137 | 9.12E-03 |
| 395 | 5.14 | 15.51901 | 8.40E-04 | 3.625071 | 3.95E-05 | 10.09978 | 4.18E-04 | 109.2643 | 1.48E-03 | 536.3736 | 8.94E-03 |
| 400 | 4.88 | 15.52105 | 7.96E-04 | 3.62502 | 3.76E-05 | 10.10025 | 3.97E-04 | 109.2636 | 1.41E-03 | 536.4639 | 8.49E-03 |

**Table S29.** Refined atomic positions, thermal parameters, and occupancies of β-$Pb_{0.1890(6)}V_2O_5$

| Atom | Wyck. Pos. | *x* | *y* | *z* | $U_{iso}$ (Å$^2$) | occ. |
|---|---|---|---|---|---|---|
| Pb | 4*i* | 0.0034(5) | 0 | 0.3987(1) | 0.0068(5) | 0.2836 |
| V(1) | 4*i* | 0.3358(3) | 0 | 0.0971(4) | 0.0052(3) | 1 |
| V(2) | 4*i* | 0.1164(1) | 0 | 0.1165(5) | 0.0094(4) | 1 |
| V(3) | 4*i* | 0.2845(7) | 0 | 0.4070(1) | 0.0094(5) | 1 |
| O(1) | 2*a* | 0 | 0 | 0 | 0.0141(5) | 1 |
| O(2) | 4*i* | 0.1872(6) | 0 | -0.0502(9) | 0.0143(8) | 1 |
| O(3) | 4*i* | 0.3658(3) | 0 | -0.0795(1) | 0.0029(7) | 1 |
| O(4) | 4*i* | 0.4333(4) | 0 | 0.2122(8) | 0.0197(2) | 1 |
| O(5) | 4*i* | 0.2632(3) | 0 | 02.223(0) | 0.0076(2) | 1 |
| O(6) | 4*i* | 0.1092(5) | 0 | 0.2721(4) | 0.0252(9) | 1 |
| O(7) | 4*i* | 0.2437(3) | 0 | 0.5728(9) | 0.0012(2) | 1 |
| O(8) | 4*i* | 0.3948(9) | 0 | 0.4731(6) | 0.0117(9) | 1 |

**Table S30**. Lattice parameters, standard deviations, unit cell volumes, and statistics of the sequential refinement of β-$Pb_{0.05}$ β'-$Cu_{0.5}V_2O_5$

| Temp. (K) | $R_{wp}$ (%) | *a* (Å) | std. dev. (Å) | *b* (Å) | std. dev. (Å) | *c* (Å) | std. dev. (Å) | β (°) | std. dev. (°) | Vol. (Å³) | std. dev. (Å³) |
|---|---|---|---|---|---|---|---|---|---|---|---|
| 90 | 14.13 | 15.1431507 | 2.01E-03 | 3.63277096 | 1.31E-04 | 10.0687525 | 1.24E-03 | 106.003827 | 4.57E-03 | 532.430889 | 3.13E-02 |
| 95 | 15.02 | 15.1439288 | 1.79E-03 | 3.63279394 | 1.18E-04 | 10.0685576 | 1.05E-03 | 105.999809 | 4.00E-03 | 532.462018 | 2.71E-02 |
| 100 | 15.00 | 15.1453974 | 1.79E-03 | 3.6327269 | 1.18E-04 | 10.0690681 | 1.05E-03 | 106.008219 | 3.99E-03 | 532.508409 | 2.71E-02 |
| 105 | 15.02 | 15.146847 | 1.79E-03 | 3.632704 | 1.18E-04 | 10.0694085 | 1.05E-03 | 106.015136 | 3.98E-03 | 532.555573 | 2.71E-02 |
| 110 | 14.96 | 15.148086 | 1.78E-03 | 3.63265143 | 1.18E-04 | 10.0698079 | 1.04E-03 | 106.021411 | 3.97E-03 | 532.595808 | 2.71E-02 |
| 115 | 14.90 | 15.1494251 | 1.78E-03 | 3.63261796 | 1.17E-04 | 10.0702775 | 1.04E-03 | 106.028356 | 3.95E-03 | 532.644274 | 2.70E-02 |
| 120 | 14.81 | 15.1520604 | 1.77E-03 | 3.63255959 | 1.17E-04 | 10.071107 | 1.03E-03 | 106.040663 | 3.93E-03 | 532.739361 | 2.70E-02 |
| 125 | 13.82 | 15.1507976 | 1.64E-03 | 3.63239883 | 1.08E-04 | 10.0715882 | 9.60E-04 | 106.037339 | 3.64E-03 | 532.705729 | 2.50E-02 |
| 130 | 13.62 | 15.1524296 | 1.61E-03 | 3.63234432 | 1.06E-04 | 10.0720721 | 9.45E-04 | 106.043381 | 3.58E-03 | 532.764558 | 2.46E-02 |
| 135 | 13.23 | 15.1536188 | 1.56E-03 | 3.63229174 | 1.03E-04 | 10.0725822 | 9.14E-04 | 106.049237 | 3.46E-03 | 532.809977 | 2.39E-02 |
| 140 | 13.51 | 15.1552729 | 1.60E-03 | 3.6322637 | 1.06E-04 | 10.0731991 | 9.36E-04 | 106.058327 | 3.55E-03 | 532.872327 | 2.45E-02 |
| 145 | 14.11 | 15.1576839 | 1.67E-03 | 3.63238595 | 1.10E-04 | 10.0737916 | 9.75E-04 | 106.070922 | 3.69E-03 | 532.972649 | 2.56E-02 |
| 150 | 14.56 | 15.1589006 | 1.74E-03 | 3.63236791 | 1.15E-04 | 10.0742322 | 1.01E-03 | 106.078462 | 3.83E-03 | 533.015888 | 2.66E-02 |
| 155 | 13.46 | 15.1606222 | 1.59E-03 | 3.63232566 | 1.04E-04 | 10.074713 | 9.22E-04 | 106.087036 | 3.49E-03 | 533.072662 | 2.43E-02 |
| 160 | 13.22 | 15.1622952 | 1.55E-03 | 3.63228756 | 1.02E-04 | 10.0752266 | 9.02E-04 | 106.095463 | 3.42E-03 | 533.130453 | 2.39E-02 |
| 165 | 13.99 | 15.1635341 | 1.66E-03 | 3.63226394 | 1.09E-04 | 10.0757876 | 9.64E-04 | 106.100643 | 3.65E-03 | 533.186324 | 2.56E-02 |
| 170 | 13.26 | 15.1648608 | 1.58E-03 | 3.63217941 | 1.04E-04 | 10.0764144 | 9.13E-04 | 106.107517 | 3.46E-03 | 533.235262 | 2.43E-02 |
| 175 | 13.72 | 15.1666137 | 1.64E-03 | 3.6321029 | 1.08E-04 | 10.07679 | 9.51E-04 | 106.11484 | 3.60E-03 | 533.285857 | 2.53E-02 |
| 180 | 13.43 | 15.1688264 | 1.61E-03 | 3.63215151 | 1.05E-04 | 10.0767296 | 9.30E-04 | 106.123848 | 3.52E-03 | 533.343364 | 2.48E-02 |
| 185 | 12.88 | 15.1699854 | 1.54E-03 | 3.63218249 | 1.01E-04 | 10.0771413 | 8.90E-04 | 106.127566 | 3.37E-03 | 533.400452 | 2.39E-02 |
| 190 | 13.35 | 15.1710658 | 1.59E-03 | 3.63213603 | 1.04E-04 | 10.0784475 | 9.15E-04 | 106.137988 | 3.46E-03 | 533.472691 | 2.46E-02 |
| 195 | 12.97 | 15.1728065 | 1.56E-03 | 3.6320369 | 1.01E-04 | 10.0788981 | 8.96E-04 | 106.142831 | 3.40E-03 | 533.530143 | 2.41E-02 |
| 200 | 13.40 | 15.1752932 | 1.62E-03 | 3.63212162 | 1.05E-04 | 10.0795723 | 9.28E-04 | 106.152725 | 3.51E-03 | 533.639043 | 2.50E-02 |
| 205 | 12.93 | 15.1757484 | 1.58E-03 | 3.63194678 | 1.02E-04 | 10.0800963 | 9.06E-04 | 106.156438 | 3.43E-03 | 533.647083 | 2.44E-02 |
| 210 | 13.33 | 15.1783773 | 1.62E-03 | 3.63200981 | 1.05E-04 | 10.0804715 | 9.28E-04 | 106.166093 | 3.51E-03 | 533.742596 | 2.51E-02 |
| 215 | 14.37 | 15.1804522 | 1.73E-03 | 3.63195658 | 1.13E-04 | 10.0811606 | 9.92E-04 | 106.174503 | 3.76E-03 | 533.821504 | 2.68E-02 |
| 220 | 14.08 | 15.1817694 | 1.70E-03 | 3.63183537 | 1.09E-04 | 10.0819048 | 9.71E-04 | 106.184906 | 3.67E-03 | 533.861294 | 2.63E-02 |
| 225 | 12.18 | 15.1834765 | 1.46E-03 | 3.63199032 | 9.43E-05 | 10.0828253 | 8.30E-04 | 106.194294 | 3.14E-03 | 533.967448 | 2.25E-02 |
| 230 | 14.75 | 15.1865256 | 1.78E-03 | 3.6320081 | 1.16E-04 | 10.0833114 | 1.02E-03 | 106.206592 | 3.84E-03 | 534.069736 | 2.76E-02 |
| 235 | 14.81 | 15.1886016 | 1.79E-03 | 3.63201213 | 1.16E-04 | 10.0839206 | 1.02E-03 | 106.2161 | 3.86E-03 | 534.149834 | 2.77E-02 |
| 240 | 14.75 | 15.1903557 | 1.79E-03 | 3.63200641 | 1.15E-04 | 10.0844737 | 1.02E-03 | 106.224358 | 3.84E-03 | 534.217585 | 2.76E-02 |
| 245 | 14.71 | 15.1923299 | 1.78E-03 | 3.63202121 | 1.15E-04 | 10.0850685 | 1.02E-03 | 106.233026 | 3.84E-03 | 534.297175 | 2.76E-02 |
| 250 | 14.68 | 15.1942557 | 1.78E-03 | 3.63202083 | 1.15E-04 | 10.0856188 | 1.02E-03 | 106.241424 | 3.83E-03 | 534.371192 | 2.75E-02 |
| 255 | 14.67 | 15.1961763 | 1.78E-03 | 3.63203919 | 1.14E-04 | 10.0861905 | 1.02E-03 | 106.250384 | 3.83E-03 | 534.447382 | 2.75E-02 |
| 260 | 14.62 | 15.1980762 | 1.78E-03 | 3.63205766 | 1.14E-04 | 10.0867658 | 1.01E-03 | 106.25949 | 3.82E-03 | 534.522635 | 2.74E-02 |
| 265 | 14.59 | 15.1999988 | 1.77E-03 | 3.63206789 | 1.14E-04 | 10.0873215 | 1.01E-03 | 106.268486 | 3.81E-03 | 534.596726 | 2.73E-02 |
| 270 | 14.60 | 15.2019566 | 1.78E-03 | 3.63206777 | 1.14E-04 | 10.0879459 | 1.02E-03 | 106.277787 | 3.83E-03 | 534.673324 | 2.74E-02 |
| 275 | 14.54 | 15.2040155 | 1.78E-03 | 3.63203669 | 1.14E-04 | 10.0886102 | 1.02E-03 | 106.285841 | 3.81E-03 | 534.754418 | 2.73E-02 |
| 280 | 14.56 | 15.2059043 | 1.78E-03 | 3.63206023 | 1.14E-04 | 10.0892793 | 1.02E-03 | 106.293633 | 3.83E-03 | 534.838536 | 2.73E-02 |
| 285 | 14.55 | 15.2078188 | 1.78E-03 | 3.63203327 | 1.14E-04 | 10.0899678 | 1.02E-03 | 106.301479 | 3.83E-03 | 534.916987 | 2.73E-02 |
| 290 | 14.55 | 15.2098094 | 1.79E-03 | 3.63200855 | 1.14E-04 | 10.090615 | 1.02E-03 | 106.309217 | 3.84E-03 | 534.996539 | 2.73E-02 |
| 295 | 14.54 | 15.2116456 | 1.79E-03 | 3.63200302 | 1.14E-04 | 10.0912272 | 1.03E-03 | 106.316543 | 3.84E-03 | 535.07275 | 2.73E-02 |
| 300 | 14.61 | 15.213572 | 1.80E-03 | 3.63202519 | 1.15E-04 | 10.091878 | 1.03E-03 | 106.323543 | 3.87E-03 | 535.159149 | 2.74E-02 |
| 310 | 14.56 | 15.2155614 | 1.80E-03 | 3.63197275 | 1.14E-04 | 10.0925603 | 1.03E-03 | 106.330693 | 3.87E-03 | 535.238018 | 2.73E-02 |
| 315 | 14.60 | 15.2175758 | 1.81E-03 | 3.63197844 | 1.15E-04 | 10.0932406 | 1.04E-03 | 106.337901 | 3.89E-03 | 535.326064 | 2.74E-02 |
| 320 | 14.59 | 15.2195646 | 1.81E-03 | 3.63195554 | 1.15E-04 | 10.0938973 | 1.04E-03 | 106.345126 | 3.90E-03 | 535.40769 | 2.74E-02 |
| 325 | 14.58 | 15.2214897 | 1.82E-03 | 3.63194795 | 1.15E-04 | 10.0945871 | 1.04E-03 | 106.35215 | 3.90E-03 | 535.491626 | 2.73E-02 |
| 330 | 14.63 | 15.223089 | 1.83E-03 | 3.63192937 | 1.15E-04 | 10.0951612 | 1.05E-03 | 106.358146 | 3.93E-03 | 535.559166 | 2.74E-02 |
| 335 | 14.62 | 15.2248797 | 1.83E-03 | 3.63190879 | 1.15E-04 | 10.0957883 | 1.05E-03 | 106.364967 | 3.93E-03 | 535.633675 | 2.74E-02 |
| 340 | 14.65 | 15.2268513 | 1.84E-03 | 3.63188237 | 1.16E-04 | 10.0964301 | 1.06E-03 | 106.372039 | 3.95E-03 | 535.713779 | 2.75E-02 |
| 345 | 14.62 | 15.2287709 | 1.84E-03 | 3.63182826 | 1.15E-04 | 10.0970866 | 1.06E-03 | 106.378906 | 3.95E-03 | 535.789298 | 2.74E-02 |
| 350 | 14.69 | 15.2309184 | 1.85E-03 | 3.63177013 | 1.16E-04 | 10.0974533 | 1.07E-03 | 106.386537 | 3.99E-03 | 535.854755 | 2.75E-02 |
| 355 | 14.69 | 15.232971 | 1.86E-03 | 3.63171446 | 1.16E-04 | 10.0981843 | 1.07E-03 | 106.393663 | 3.99E-03 | 535.937947 | 2.75E-02 |
| 360 | 14.73 | 15.2348381 | 1.86E-03 | 3.63165253 | 1.16E-04 | 10.0988628 | 1.08E-03 | 106.400475 | 4.01E-03 | 536.011757 | 2.75E-02 |
| 365 | 14.75 | 15.2367442 | 1.87E-03 | 3.63162572 | 1.16E-04 | 10.0994852 | 1.08E-03 | 106.407574 | 4.02E-03 | 536.088343 | 2.75E-02 |
| 370 | 14.76 | 15.2386591 | 1.87E-03 | 3.63160001 | 1.16E-04 | 10.1001227 | 1.08E-03 | 106.414619 | 4.04E-03 | 536.166352 | 2.75E-02 |
| 375 | 14.82 | 15.2406162 | 1.89E-03 | 3.63154764 | 1.17E-04 | 10.1007546 | 1.09E-03 | 106.421491 | 4.06E-03 | 536.242078 | 2.76E-02 |
| 380 | 14.73 | 15.2424944 | 1.88E-03 | 3.63153314 | 1.16E-04 | 10.1014092 | 1.09E-03 | 106.427785 | 4.05E-03 | 536.323403 | 2.74E-02 |
| 385 | 14.73 | 15.2442107 | 1.88E-03 | 3.63152203 | 1.17E-04 | 10.1020291 | 1.09E-03 | 106.434033 | 4.05E-03 | 536.397819 | 2.74E-02 |
| 390 | 14.75 | 15.2464937 | 1.89E-03 | 3.63147197 | 1.17E-04 | 10.1027997 | 1.09E-03 | 106.442315 | 4.07E-03 | 536.488796 | 2.75E-02 |
| 395 | 14.72 | 15.2484126 | 1.89E-03 | 3.63148067 | 1.17E-04 | 10.1034261 | 1.09E-03 | 106.449022 | 4.07E-03 | 536.572335 | 2.74E-02 |
| 400 | 14.74 | 15.2503407 | 1.90E-03 | 3.63146301 | 1.18E-04 | 10.104051 | 1.10E-03 | 106.456246 | 4.09E-03 | 536.650781 | 2.75E-02 |

**Table S31.** Refined atomic positions, thermal parameters, and occupancies of β-$Pb_{0.0253(3)}$ β’-$Cu_{0.452(9)}V_2O_5$

| Atom | Wyck. Pos. | *x* | *y* | *z* | $U_{iso}$ (Å$^2$) | occ. |
|---|---|---|---|---|---|---|
| Cu(1) | 4*i* | 0.5478(7) | 0 | 0.3409(2) | 0.1158(2) | 0.1158(2) |
| Cu(2) | 8*j* | 0.5247(3) | 0.0161(9) | 0.3644(5) | 0.0143(9) | 0.0143(9) |
| Pb | 4*i* | 0.0220(1) | 0 | 0.3927(6) | 0.0522(0) | 0.0041(7) |
| V(1) | 4*i* | 0.3355(1) | 0 | 0.0888(0) | 0.0041(7) | 1 |
| V(2) | 4*i* | 0.1128(6) | 0 | 0.1237(0) | 0.0106(7) | 1 |
| V(3) | 4*i* | 0.2878(4) | 0 | 0.4070(1) | 0.0173(4) | 1 |
| O(1) | 2*a* | 0 | 0 | 0 | 0.0140(0) | 1 |
| O(2) | 4*i* | 0.1916(4) | 0 | -0.0281(3) | 0.0227(7) | 1 |
| O(3) | 4*i* | 0.3568(8) | 0 | -0.0749() | 0.0051(0) | 1 |
| O(4) | 4*i* | 0.4335(3) | 0 | 0.1978(9) | 0.0107(2) | 1 |
| O(5) | 4*i* | 0.2691(4) | 0 | 0.2406(0) | 0.0520(0) | 1 |
| O(6) | 4*i* | 0.0851(0) | 0 | 0.2842(5) | 0.0604(9) | 1 |
| O(7) | 4*i* | 0.2499(3) | 0 | 0.5946(7) | 0.0184(5) | 1 |
| O(8) | 4*i* | 0.3973(4) | 0 | 0.4518(9) | 0.0303(2) | 1 |

| **Table S32.** Lattice paramters, unit cell volumes, standard deviations and refinement statistics for the sequential refinement of β'-$Cu_{0.33}V_2O_5$ | | | | | | | | | | | |
|---|---|---|---|---|---|---|---|---|---|---|---|
| Temp. (K) | Rwp (%) | *a* (Å) | std. dev. (Å) | *b* (Å) | std. dev. (Å) | *c* (Å) | std. dev (Å) | β (°) | std. dev. (°) | Vol (Å³) | std. dev. (Å³) |
| 90 | 12.91 | 15.0880 | 1.32E-03 | 3.62172 | 8.31E-05 | 10.0608 | 7.47E-04 | 106.510 | 2.82E-03 | 527.098 | 1.87E-02 |
| 95 | 12.90 | 15.0893 | 1.31E-03 | 3.62171 | 8.31E-05 | 10.0610 | 7.46E-04 | 106.514 | 2.82E-03 | 527.144 | 1.87E-02 |
| 100 | 12.91 | 15.0914 | 1.32E-03 | 3.62163 | 8.32E-05 | 10.0615 | 7.48E-04 | 106.521 | 2.82E-03 | 527.212 | 1.87E-02 |
| 105 | 12.84 | 15.0936 | 1.31E-03 | 3.62155 | 8.28E-05 | 10.0619 | 7.45E-04 | 106.528 | 2.81E-03 | 527.277 | 1.86E-02 |
| 110 | 12.74 | 15.0957 | 1.30E-03 | 3.62143 | 8.23E-05 | 10.0623 | 7.40E-04 | 106.535 | 2.79E-03 | 527.339 | 1.85E-02 |
| 115 | 12.67 | 15.0980 | 1.31E-03 | 3.62130 | 8.22E-05 | 10.0627 | 7.39E-04 | 106.541 | 2.80E-03 | 527.406 | 1.85E-02 |
| 120 | 12.70 | 15.1003 | 1.31E-03 | 3.62132 | 8.26E-05 | 10.0632 | 7.46E-04 | 106.548 | 2.81E-03 | 527.492 | 1.86E-02 |
| 125 | 12.66 | 15.1023 | 1.31E-03 | 3.62117 | 8.22E-05 | 10.0636 | 7.45E-04 | 106.555 | 2.81E-03 | 527.546 | 1.85E-02 |
| 130 | 12.66 | 15.1049 | 1.31E-03 | 3.62110 | 8.20E-05 | 10.0641 | 7.46E-04 | 106.563 | 2.81E-03 | 527.631 | 1.85E-02 |
| 135 | 12.51 | 15.1074 | 1.29E-03 | 3.62106 | 8.03E-05 | 10.0645 | 7.30E-04 | 106.568 | 2.75E-03 | 527.717 | 1.81E-02 |
| 140 | 12.40 | 15.1100 | 1.27E-03 | 3.62097 | 7.99E-05 | 10.0650 | 7.24E-04 | 106.574 | 2.73E-03 | 527.806 | 1.80E-02 |
| 145 | 12.17 | 15.1130 | 1.26E-03 | 3.62087 | 7.86E-05 | 10.0656 | 7.14E-04 | 106.582 | 2.69E-03 | 527.906 | 1.77E-02 |
| 150 | 12.03 | 15.1156 | 1.24E-03 | 3.62078 | 7.74E-05 | 10.0662 | 7.06E-04 | 106.591 | 2.65E-03 | 527.989 | 1.75E-02 |
| 155 | 11.86 | 15.1182 | 1.22E-03 | 3.62069 | 7.65E-05 | 10.0670 | 6.94E-04 | 106.600 | 2.61E-03 | 528.083 | 1.73E-02 |
| 160 | 11.77 | 15.1213 | 1.22E-03 | 3.62061 | 7.58E-05 | 10.0675 | 6.94E-04 | 106.610 | 2.60E-03 | 528.182 | 1.72E-02 |
| 165 | 11.64 | 15.1241 | 1.21E-03 | 3.62052 | 7.59E-05 | 10.0682 | 6.86E-04 | 106.617 | 2.58E-03 | 528.282 | 1.71E-02 |
| 170 | 11.82 | 15.1269 | 1.22E-03 | 3.62045 | 7.80E-05 | 10.0690 | 6.86E-04 | 106.626 | 2.59E-03 | 528.386 | 1.74E-02 |
| 175 | 11.82 | 15.1303 | 1.22E-03 | 3.62033 | 7.75E-05 | 10.0697 | 6.92E-04 | 106.635 | 2.60E-03 | 528.499 | 1.74E-02 |
| 180 | 12.03 | 15.1338 | 1.27E-03 | 3.62025 | 7.93E-05 | 10.0702 | 7.22E-04 | 106.645 | 2.71E-03 | 528.609 | 1.80E-02 |
| 185 | 13.11 | 15.1370 | 1.40E-03 | 3.62010 | 8.73E-05 | 10.0709 | 7.94E-04 | 106.654 | 2.97E-03 | 528.712 | 1.99E-02 |
| 190 | 12.29 | 15.1399 | 1.31E-03 | 3.62000 | 8.20E-05 | 10.0720 | 7.45E-04 | 106.662 | 2.78E-03 | 528.835 | 1.87E-02 |
| 195 | 14.47 | 15.1436 | 1.62E-03 | 3.61987 | 1.01E-04 | 10.0731 | 9.30E-04 | 106.671 | 3.45E-03 | 528.974 | 2.32E-02 |
| 200 | 14.22 | 15.1476 | 1.58E-03 | 3.61981 | 9.70E-05 | 10.0740 | 9.00E-04 | 106.683 | 3.34E-03 | 529.120 | 2.25E-02 |
| 205 | 13.10 | 15.1526 | 1.45E-03 | 3.61965 | 8.91E-05 | 10.0755 | 8.24E-04 | 106.698 | 3.05E-03 | 529.308 | 2.07E-02 |
| 210 | 14.02 | 15.1581 | 1.58E-03 | 3.61953 | 9.68E-05 | 10.0769 | 9.03E-04 | 106.714 | 3.33E-03 | 529.513 | 2.27E-02 |
| 215 | 11.91 | 15.1657 | 1.33E-03 | 3.61930 | 8.18E-05 | 10.0787 | 7.63E-04 | 106.735 | 2.81E-03 | 529.784 | 1.92E-02 |
| 220 | 12.70 | 15.1739 | 1.44E-03 | 3.61890 | 8.77E-05 | 10.0817 | 8.22E-04 | 106.757 | 3.02E-03 | 530.105 | 2.07E-02 |
| 225 | 13.83 | 15.1783 | 1.64E-03 | 3.61882 | 9.95E-05 | 10.0830 | 9.39E-04 | 106.768 | 3.43E-03 | 530.284 | 2.35E-02 |
| 230 | 14.87 | 15.1803 | 1.75E-03 | 3.61868 | 1.06E-04 | 10.0839 | 9.98E-04 | 106.789 | 3.66E-03 | 530.328 | 2.50E-02 |
| 235 | 6.67 | 15.1880 | 6.98E-04 | 3.61878 | 4.25E-05 | 10.0846 | 3.97E-04 | 106.802 | 1.46E-03 | 530.610 | 9.84E-03 |
| 240 | 6.73 | 15.1907 | 6.99E-04 | 3.61873 | 4.31E-05 | 10.0855 | 3.97E-04 | 106.811 | 1.46E-03 | 530.719 | 9.85E-03 |
| 245 | 6.74 | 15.1934 | 7.06E-04 | 3.61865 | 4.32E-05 | 10.0862 | 4.03E-04 | 106.822 | 1.48E-03 | 530.808 | 9.88E-03 |
| 250 | 6.82 | 15.1963 | 7.15E-04 | 3.61865 | 4.38E-05 | 10.0869 | 4.08E-04 | 106.833 | 1.50E-03 | 530.914 | 9.97E-03 |
| 255 | 6.75 | 15.1992 | 7.08E-04 | 3.61856 | 4.33E-05 | 10.0877 | 4.06E-04 | 106.844 | 1.49E-03 | 531.013 | 9.84E-03 |
| 260 | 6.80 | 15.2019 | 7.17E-04 | 3.61850 | 4.38E-05 | 10.0884 | 4.12E-04 | 106.854 | 1.51E-03 | 531.107 | 9.92E-03 |
| 265 | 6.85 | 15.2047 | 7.23E-04 | 3.61841 | 4.42E-05 | 10.0892 | 4.17E-04 | 106.864 | 1.52E-03 | 531.205 | 9.96E-03 |
| 270 | 6.80 | 15.2074 | 7.21E-04 | 3.61836 | 4.39E-05 | 10.0899 | 4.17E-04 | 106.874 | 1.52E-03 | 531.300 | 9.87E-03 |
| 275 | 6.83 | 15.2102 | 7.32E-04 | 3.61831 | 4.43E-05 | 10.0907 | 4.25E-04 | 106.885 | 1.55E-03 | 531.403 | 9.96E-03 |
| 280 | 6.75 | 15.2128 | 7.21E-04 | 3.61825 | 4.35E-05 | 10.0914 | 4.18E-04 | 106.894 | 1.52E-03 | 531.497 | 9.75E-03 |
| 285 | 6.76 | 15.2155 | 7.23E-04 | 3.61823 | 4.35E-05 | 10.0922 | 4.20E-04 | 106.905 | 1.53E-03 | 531.599 | 9.74E-03 |
| 290 | 6.82 | 15.2182 | 7.32E-04 | 3.61814 | 4.40E-05 | 10.0929 | 4.25E-04 | 106.914 | 1.55E-03 | 531.690 | 9.82E-03 |
| 295 | 6.80 | 15.2207 | 7.28E-04 | 3.61807 | 4.38E-05 | 10.0936 | 4.24E-04 | 106.924 | 1.54E-03 | 531.779 | 9.75E-03 |
| 300 | 6.76 | 15.2231 | 7.26E-04 | 3.61803 | 4.36E-05 | 10.0943 | 4.22E-04 | 106.933 | 1.53E-03 | 531.867 | 9.69E-03 |
| 305 | 6.76 | 15.2259 | 7.27E-04 | 3.61796 | 4.36E-05 | 10.0949 | 4.23E-04 | 106.942 | 1.54E-03 | 531.960 | 9.67E-03 |
| 310 | 6.70 | 15.2284 | 7.21E-04 | 3.61792 | 4.32E-05 | 10.0956 | 4.20E-04 | 106.953 | 1.52E-03 | 532.051 | 9.57E-03 |
| 315 | 6.74 | 15.2309 | 7.26E-04 | 3.61785 | 4.37E-05 | 10.0964 | 4.23E-04 | 106.962 | 1.53E-03 | 532.142 | 9.63E-03 |
| 320 | 6.76 | 15.2332 | 7.28E-04 | 3.61780 | 4.38E-05 | 10.0970 | 4.25E-04 | 106.970 | 1.54E-03 | 532.223 | 9.66E-03 |
| 325 | 6.76 | 15.2357 | 7.29E-04 | 3.61774 | 4.38E-05 | 10.0978 | 4.26E-04 | 106.980 | 1.54E-03 | 532.316 | 9.65E-03 |
| 330 | 6.69 | 15.2379 | 7.22E-04 | 3.61771 | 4.34E-05 | 10.0984 | 4.18E-04 | 106.988 | 1.52E-03 | 532.401 | 9.52E-03 |
| 335 | 6.67 | 15.2405 | 7.20E-04 | 3.61761 | 4.31E-05 | 10.0991 | 4.21E-04 | 106.998 | 1.52E-03 | 532.481 | 9.51E-03 |
| 340 | 6.77 | 15.2430 | 7.29E-04 | 3.61754 | 4.38E-05 | 10.0998 | 4.25E-04 | 107.007 | 1.54E-03 | 532.569 | 9.63E-03 |
| 345 | 6.78 | 15.2451 | 7.38E-04 | 3.61749 | 4.41E-05 | 10.1003 | 4.31E-04 | 107.015 | 1.55E-03 | 532.643 | 9.74E-03 |
| 350 | 6.82 | 15.2472 | 7.37E-04 | 3.61743 | 4.42E-05 | 10.1009 | 4.31E-04 | 107.023 | 1.55E-03 | 532.716 | 9.74E-03 |
| 355 | 6.85 | 15.2496 | 7.42E-04 | 3.61736 | 4.45E-05 | 10.1015 | 4.35E-04 | 107.031 | 1.56E-03 | 532.795 | 9.81E-03 |
| 360 | 6.91 | 15.2515 | 7.49E-04 | 3.61729 | 4.51E-05 | 10.1020 | 4.38E-04 | 107.040 | 1.57E-03 | 532.854 | 9.90E-03 |
| 365 | 6.92 | 15.2539 | 7.54E-04 | 3.61723 | 4.52E-05 | 10.1027 | 4.43E-04 | 107.049 | 1.59E-03 | 532.937 | 9.97E-03 |
| 370 | 6.85 | 15.2564 | 7.46E-04 | 3.61721 | 4.48E-05 | 10.1033 | 4.39E-04 | 107.058 | 1.57E-03 | 533.029 | 9.88E-03 |
| 375 | 6.90 | 15.2589 | 7.51E-04 | 3.61711 | 4.51E-05 | 10.1039 | 4.42E-04 | 107.067 | 1.58E-03 | 533.108 | 9.95E-03 |
| 380 | 6.84 | 15.2608 | 7.33E-04 | 3.61705 | 4.44E-05 | 10.1046 | 4.26E-04 | 107.075 | 1.53E-03 | 533.180 | 9.69E-03 |
| 385 | 6.78 | 15.2630 | 7.28E-04 | 3.61705 | 4.40E-05 | 10.1053 | 4.24E-04 | 107.083 | 1.52E-03 | 533.270 | 9.63E-03 |
| 390 | 6.83 | 15.2658 | 7.44E-04 | 3.61695 | 4.46E-05 | 10.1061 | 4.38E-04 | 107.094 | 1.56E-03 | 533.361 | 9.86E-03 |
| 395 | 7.14 | 15.2679 | 7.66E-04 | 3.61689 | 4.63E-05 | 10.1067 | 4.48E-04 | 107.101 | 1.60E-03 | 533.441 | 1.02E-02 |

**Table S33.** Refined atomic positions, thermal parameters and occupancies for β'-$Cu_{0.33(3)}V_2O_5$

| Atom | Wyck. Pos. | *x* | *y* | *z* | Occ. | $U_{iso}$ (Å$^2$) |
|---|---|---|---|---|---|---|
| Cu(1) | 4*i* | 0.5373(8) | 0 | 0.3531(11) | 0.206(23) | 0.010(5) |
| Cu(2) | 8*j* | 0.5210(12) | -0.130(8) | 0.3685(14) | 0.143(11) | 0.041(6) |
| V(1) | 4*i* | 0.11377(15) | 0 | 0.11967(23) | 1 | 0.0036(6) |
| V(2) | 4*i* | 0.33441(15) | 0 | 0.09439(22) | 1 | 0.0077(7) |
| V(3) | 4*i* | 0.28665(17) | 0 | 0.40689(26) | 1 | 0.0067(6) |
| O(1) | 4*i* | 0.0987(5) | 0 | 0.2738(6) | 1 | 0.0231(25) |
| O(2) | 2*a* | 0 | 0 | 0 | 1 | 0.013(3) |
| O(3) | 4*i* | 0.2601(5) | 0 | 0.2225(7) | 1 | 0.0182(23) |
| O(4) | 4*i* | 0.1869(5) | 0 | -0.0506(8) | 1 | 0.0196(24) |
| O(5) | 4*i* | 0.4304(5) | 0 | 0.2032(8) | 1 | 0.0357(29) |
| O(6) | 4*i* | 0.3674(5) | 0 | -0.0832(7) | 1 | 0.0140(21) |
| O(7) | 4*i* | 0.3920(5) | 0 | 0.4603(9) | 1 | 0.048(3) |
| O(8) | 4*i* | 0.2442(5) | 0 | 0.5749(7) | 1 | 0.0230(24) |

| **Table S34.** Lattice paramters, unit cell volumes, standard deviations and refinement statistics for the sequential refinement of β'-$Cu_{0.50}V_2O_5$ | | | | | | | | | | | |
|---|---|---|---|---|---|---|---|---|---|---|---|
| Temp. (K) | Rwp (%) | *a* (Å) | std. dev. (Å) | *b* (Å) | std. dev. (Å) | *c* (Å) | std. dev (Å) | β (°) | std. dev. (°) | Vol (Å$^3$) | std. dev. (Å$^3$) |
| 90 | 6.91 | 15.1545 | 6.33E-04 | 3.63021 | 4.38657E-05 | 10.0747 | 3.63E-04 | 106.144 | 1.37E-03 | 532.393 | 1.00E-02 |
| 95 | 6.90 | 15.1558 | 6.33E-04 | 3.63013 | 4.38571E-05 | 10.0749 | 3.63E-04 | 106.149 | 1.37E-03 | 532.425 | 1.00E-02 |
| 100 | 6.72 | 15.1574 | 6.13E-04 | 3.63005 | 4.25491E-05 | 10.0753 | 3.51E-04 | 106.155 | 1.33E-03 | 532.471 | 9.71E-03 |
| 105 | 6.57 | 15.1588 | 5.98E-04 | 3.62997 | 4.15086E-05 | 10.0756 | 3.41E-04 | 106.162 | 1.29E-03 | 532.511 | 9.48E-03 |
| 110 | 6.29 | 15.1603 | 5.69E-04 | 3.62994 | 3.95225E-05 | 10.0761 | 3.24E-04 | 106.169 | 1.23E-03 | 532.562 | 9.02E-03 |
| 115 | 6.26 | 15.1618 | 5.66E-04 | 3.62994 | 3.9303E-05 | 10.0764 | 3.22E-04 | 106.175 | 1.22E-03 | 532.619 | 8.96E-03 |
| 120 | 6.26 | 15.1631 | 5.66E-04 | 3.62991 | 3.93991E-05 | 10.0767 | 3.22E-04 | 106.181 | 1.22E-03 | 532.658 | 8.98E-03 |
| 125 | 6.15 | 15.1659 | 5.59E-04 | 3.62982 | 3.8673E-05 | 10.0770 | 3.20E-04 | 106.183 | 1.21E-03 | 532.754 | 8.87E-03 |
| 130 | 5.99 | 15.1663 | 5.41E-04 | 3.62981 | 3.74348E-05 | 10.0776 | 3.07E-04 | 106.196 | 1.16E-03 | 532.760 | 8.56E-03 |
| 135 | 5.91 | 15.1692 | 5.36E-04 | 3.62971 | 3.69006E-05 | 10.0778 | 3.06E-04 | 106.198 | 1.16E-03 | 532.855 | 8.50E-03 |
| 140 | 5.94 | 15.1706 | 5.40E-04 | 3.62966 | 3.70741E-05 | 10.0782 | 3.08E-04 | 106.204 | 1.16E-03 | 532.903 | 8.56E-03 |
| 145 | 6.12 | 15.1720 | 5.58E-04 | 3.62965 | 3.83264E-05 | 10.0788 | 3.18E-04 | 106.213 | 1.20E-03 | 532.958 | 8.83E-03 |
| 150 | 6.00 | 15.1735 | 5.47E-04 | 3.62960 | 3.75351E-05 | 10.0792 | 3.12E-04 | 106.220 | 1.18E-03 | 533.004 | 8.66E-03 |
| 155 | 5.95 | 15.1750 | 5.43E-04 | 3.62957 | 3.72304E-05 | 10.0796 | 3.10E-04 | 106.228 | 1.17E-03 | 533.055 | 8.59E-03 |
| 160 | 5.85 | 15.1768 | 5.36E-04 | 3.62954 | 3.6628E-05 | 10.0800 | 3.06E-04 | 106.233 | 1.15E-03 | 533.116 | 8.47E-03 |
| 165 | 5.74 | 15.1785 | 5.25E-04 | 3.62951 | 3.58939E-05 | 10.0805 | 2.99E-04 | 106.242 | 1.13E-03 | 533.178 | 8.29E-03 |
| 170 | 5.71 | 15.1802 | 5.24E-04 | 3.62947 | 3.57836E-05 | 10.0810 | 2.98E-04 | 106.250 | 1.12E-03 | 533.236 | 8.27E-03 |
| 175 | 5.69 | 15.1819 | 5.22E-04 | 3.62944 | 3.56367E-05 | 10.0815 | 2.96E-04 | 106.258 | 1.12E-03 | 533.295 | 8.23E-03 |
| 180 | 5.62 | 15.1836 | 5.17E-04 | 3.62941 | 3.52445E-05 | 10.0819 | 2.93E-04 | 106.265 | 1.11E-03 | 533.353 | 8.13E-03 |
| 185 | 5.52 | 15.1856 | 5.08E-04 | 3.62938 | 3.45994E-05 | 10.0825 | 2.88E-04 | 106.275 | 1.09E-03 | 533.423 | 7.99E-03 |
| 190 | 5.45 | 15.1870 | 5.02E-04 | 3.62935 | 3.41572E-05 | 10.0829 | 2.84E-04 | 106.282 | 1.07E-03 | 533.472 | 7.89E-03 |
| 195 | 5.45 | 15.1885 | 5.03E-04 | 3.62933 | 3.42056E-05 | 10.0834 | 2.85E-04 | 106.289 | 1.07E-03 | 533.525 | 7.88E-03 |
| 200 | 5.41 | 15.1902 | 4.99E-04 | 3.62930 | 3.39523E-05 | 10.0838 | 2.82E-04 | 106.297 | 1.07E-03 | 533.582 | 7.81E-03 |
| 205 | 5.31 | 15.1920 | 4.92E-04 | 3.62928 | 3.33369E-05 | 10.0843 | 2.78E-04 | 106.305 | 1.05E-03 | 533.645 | 7.68E-03 |
| 210 | 5.23 | 15.1937 | 4.85E-04 | 3.62926 | 3.28324E-05 | 10.0847 | 2.74E-04 | 106.313 | 1.03E-03 | 533.704 | 7.57E-03 |
| 215 | 5.10 | 15.1958 | 4.73E-04 | 3.62925 | 3.19125E-05 | 10.0853 | 2.67E-04 | 106.323 | 1.01E-03 | 533.778 | 7.36E-03 |
| 220 | 4.96 | 15.1994 | 4.63E-04 | 3.62925 | 3.111E-05 | 10.0863 | 2.62E-04 | 106.341 | 9.87E-04 | 533.911 | 7.17E-03 |
| 225 | 4.93 | 15.2000 | 4.60E-04 | 3.62926 | 3.09087E-05 | 10.0864 | 2.59E-04 | 106.344 | 9.77E-04 | 533.931 | 7.11E-03 |
| 230 | 4.92 | 15.2014 | 4.60E-04 | 3.62924 | 3.08948E-05 | 10.0868 | 2.59E-04 | 106.350 | 9.79E-04 | 533.983 | 7.11E-03 |
| 235 | 4.89 | 15.2032 | 4.58E-04 | 3.62922 | 3.06925E-05 | 10.0873 | 2.58E-04 | 106.358 | 9.75E-04 | 534.046 | 7.06E-03 |
| 240 | 4.84 | 15.2049 | 4.55E-04 | 3.62919 | 3.03794E-05 | 10.0879 | 2.56E-04 | 106.365 | 9.67E-04 | 534.110 | 6.99E-03 |
| 245 | 4.79 | 15.2067 | 4.51E-04 | 3.62915 | 3.00941E-05 | 10.0884 | 2.54E-04 | 106.373 | 9.59E-04 | 534.177 | 6.92E-03 |
| 250 | 4.76 | 15.2087 | 4.49E-04 | 3.62912 | 2.99033E-05 | 10.0890 | 2.53E-04 | 106.380 | 9.53E-04 | 534.252 | 6.86E-03 |
| 255 | 4.77 | 15.2106 | 4.51E-04 | 3.62909 | 3.00335E-05 | 10.0896 | 2.54E-04 | 106.388 | 9.58E-04 | 534.324 | 6.89E-03 |
| 260 | 4.71 | 15.2125 | 4.45E-04 | 3.62905 | 2.96091E-05 | 10.0902 | 2.50E-04 | 106.396 | 9.44E-04 | 534.395 | 6.77E-03 |
| 265 | 4.65 | 15.2144 | 4.40E-04 | 3.62901 | 2.92545E-05 | 10.0908 | 2.48E-04 | 106.403 | 9.34E-04 | 534.471 | 6.69E-03 |
| 270 | 4.69 | 15.2163 | 4.45E-04 | 3.62895 | 2.95256E-05 | 10.0914 | 2.50E-04 | 106.410 | 9.45E-04 | 534.541 | 6.75E-03 |
| 275 | 4.61 | 15.2182 | 4.38E-04 | 3.62891 | 2.89867E-05 | 10.0920 | 2.46E-04 | 106.417 | 9.28E-04 | 534.616 | 6.61E-03 |
| 280 | 4.61 | 15.2202 | 4.40E-04 | 3.62887 | 2.90799E-05 | 10.0927 | 2.48E-04 | 106.424 | 9.33E-04 | 534.693 | 6.63E-03 |
| 285 | 4.59 | 15.2222 | 4.39E-04 | 3.62884 | 2.8969E-05 | 10.0933 | 2.48E-04 | 106.432 | 9.31E-04 | 534.773 | 6.61E-03 |
| 290 | 4.57 | 15.2241 | 4.38E-04 | 3.62879 | 2.8876E-05 | 10.0939 | 2.47E-04 | 106.439 | 9.28E-04 | 534.844 | 6.57E-03 |
| 295 | 4.58 | 15.2260 | 4.38E-04 | 3.62874 | 2.89074E-05 | 10.0946 | 2.47E-04 | 106.446 | 9.29E-04 | 534.921 | 6.57E-03 |
| 300 | 4.56 | 15.2281 | 4.37E-04 | 3.62870 | 2.88532E-05 | 10.0953 | 2.46E-04 | 106.453 | 9.25E-04 | 535.003 | 6.53E-03 |
| 305 | 4.55 | 15.2300 | 4.37E-04 | 3.62867 | 2.88055E-05 | 10.0959 | 2.46E-04 | 106.460 | 9.25E-04 | 535.081 | 6.53E-03 |
| 310 | 4.61 | 15.2320 | 4.46E-04 | 3.62862 | 2.93925E-05 | 10.0965 | 2.52E-04 | 106.467 | 9.44E-04 | 535.155 | 6.65E-03 |
| 315 | 4.64 | 15.2334 | 4.46E-04 | 3.62857 | 2.94498E-05 | 10.0970 | 2.51E-04 | 106.472 | 9.43E-04 | 535.211 | 6.64E-03 |
| 320 | 4.62 | 15.2353 | 4.46E-04 | 3.62852 | 2.93605E-05 | 10.0977 | 2.51E-04 | 106.479 | 9.42E-04 | 535.285 | 6.63E-03 |
| 325 | 4.61 | 15.2371 | 4.45E-04 | 3.62848 | 2.92768E-05 | 10.0983 | 2.50E-04 | 106.486 | 9.39E-04 | 535.360 | 6.60E-03 |
| 330 | 4.65 | 15.2390 | 4.49E-04 | 3.62844 | 2.95458E-05 | 10.0989 | 2.53E-04 | 106.492 | 9.48E-04 | 535.437 | 6.66E-03 |
| 335 | 4.61 | 15.2408 | 4.47E-04 | 3.62840 | 2.9322E-05 | 10.0996 | 2.51E-04 | 106.498 | 9.42E-04 | 535.509 | 6.61E-03 |
| 340 | 4.64 | 15.2429 | 4.50E-04 | 3.62835 | 2.95345E-05 | 10.1002 | 2.53E-04 | 106.505 | 9.49E-04 | 535.593 | 6.65E-03 |
| 345 | 4.60 | 15.2449 | 4.47E-04 | 3.62831 | 2.93354E-05 | 10.1009 | 2.51E-04 | 106.512 | 9.42E-04 | 535.671 | 6.60E-03 |
| 350 | 4.66 | 15.2467 | 4.52E-04 | 3.62828 | 2.96475E-05 | 10.1015 | 2.55E-04 | 106.518 | 9.53E-04 | 535.746 | 6.68E-03 |
| 355 | 4.62 | 15.2483 | 4.51E-04 | 3.62824 | 2.95043E-05 | 10.1020 | 2.54E-04 | 106.523 | 9.49E-04 | 535.808 | 6.65E-03 |
| 360 | 4.56 | 15.2504 | 4.44E-04 | 3.62818 | 2.90739E-05 | 10.1027 | 2.50E-04 | 106.530 | 9.35E-04 | 535.888 | 6.54E-03 |
| 365 | 4.52 | 15.2527 | 4.42E-04 | 3.62811 | 2.88745E-05 | 10.1035 | 2.49E-04 | 106.538 | 9.29E-04 | 535.980 | 6.50E-03 |
| 370 | 4.57 | 15.2544 | 4.50E-04 | 3.62810 | 2.93543E-05 | 10.1041 | 2.54E-04 | 106.544 | 9.46E-04 | 536.053 | 6.62E-03 |
| 375 | 4.59 | 15.2559 | 4.52E-04 | 3.62806 | 2.95077E-05 | 10.1046 | 2.55E-04 | 106.549 | 9.50E-04 | 536.114 | 6.65E-03 |
| 380 | 4.59 | 15.2586 | 4.53E-04 | 3.62800 | 2.95605E-05 | 10.1055 | 2.55E-04 | 106.559 | 9.51E-04 | 536.225 | 6.66E-03 |
| 385 | 4.56 | 15.2596 | 4.50E-04 | 3.62798 | 2.93957E-05 | 10.1059 | 2.54E-04 | 106.562 | 9.45E-04 | 536.263 | 6.62E-03 |
| 390 | 4.65 | 15.2625 | 4.60E-04 | 3.62791 | 3.00801E-05 | 10.1068 | 2.60E-04 | 106.572 | 9.66E-04 | 536.382 | 6.77E-03 |
| 395 | 4.62 | 15.2621 | 4.57E-04 | 3.62793 | 2.98734E-05 | 10.1067 | 2.58E-04 | 106.570 | 9.59E-04 | 536.365 | 6.72E-03 |

**Table S35.** Refined atomic positions, thermal parameters and occupancies for β'-$Cu_{0.462(22)}V_2O_5$

| Atom | Wyck. Pos. | *x* | *y* | *z* | Occ. | $U_{iso}$ (Å$^2$) |
|---|---|---|---|---|---|---|
| Cu(1) | 4*i* | 0.5430(5) | 0 | 0.3439(6) | 0.269(17) | 0.0020(23) |
| Cu(2) | 8*j* | 0.5233(5) | -0.1043(25) | 0.3645(6) | 0.212(8) | 0.0188(20) |
| V(1) | 4*i* | 0.11379(9) | 0 | 0.11952(15) | 1 | 0.0051(4) |
| V(2) | 4*i* | 0.33286(10) | 0 | 0.09180(14) | 1 | 0.0054(5) |
| V(3) | 4*i* | 0.28605(12) | 0 | 0.40639(17) | 1 | 0.0094(4) |
| O(1) | 4*i* | 0.09732(29) | 0 | 0.2730(4) | 1 | 0.0243(17) |
| O(2) | 2*a* | 0 | 0 | 0 | 1 | 0.0111(20) |
| O(3) | 4*i* | 0.25893(29) | 0 | 0.2235(4) | 1 | 0.0232(16) |
| O(4) | 4*i* | 0.18697(31) | 0 | -0.0497(5) | 1 | 0.0179(16) |
| O(5) | 4*i* | 0.4301(3) | 0 | 0.2012(5) | 1 | 0.0396(20) |
| O(6) | 4*i* | 0.36742(29) | 0 | -0.0833(4) | 1 | 0.0181(15) |
| O(7) | 4*i* | 0.3931(3) | 0 | 0.4576(5) | 1 | 0.0421(19) |
| O(8) | 4*i* | 0.2441(3) | 0 | 0.5755(4) | 1 | 0.0227(17) |

| **Table S36.** Lattice paramters, unit cell volumes, standard deviations and refinement statistics for the sequential refinement of β'-$Cu_{0.33}$/β-$Pb_{0.01}V_2O_5$ | | | | | | | | | | | |
|---|---|---|---|---|---|---|---|---|---|---|---|
| Temp. (K) | Rwp (%) | *a* (Å) | std. dev. (Å) | *b* (Å) | std. dev. (Å) | *c* (Å) | std. dev (Å) | β (°) | std. dev. (°) | Vol (Å³) | std. dev. (Å³) |
| 90 | 10.53 | 15.1102 | 1.39E-03 | 3.62342 | 7.40012E-05 | 10.0653 | 8.11E-04 | 106.513 | 3.02E-03 | 528.352 | 1.93E-02 |
| 95 | 11.04 | 15.1124 | 1.45E-03 | 3.62331 | 7.68003E-05 | 10.0657 | 8.43E-04 | 106.521 | 3.14E-03 | 528.411 | 2.00E-02 |
| 100 | 10.48 | 15.1145 | 1.36E-03 | 3.62323 | 7.23068E-05 | 10.0662 | 7.96E-04 | 106.529 | 2.96E-03 | 528.478 | 1.89E-02 |
| 105 | 10.49 | 15.1163 | 1.37E-03 | 3.62316 | 7.23528E-05 | 10.0666 | 7.97E-04 | 106.535 | 2.96E-03 | 528.536 | 1.89E-02 |
| 110 | 10.35 | 15.1188 | 1.35E-03 | 3.62306 | 7.11899E-05 | 10.0672 | 7.84E-04 | 106.544 | 2.92E-03 | 528.614 | 1.86E-02 |
| 115 | 10.00 | 15.1208 | 1.29E-03 | 3.62297 | 6.79993E-05 | 10.0676 | 7.49E-04 | 106.551 | 2.79E-03 | 528.676 | 1.78E-02 |
| 120 | 9.87 | 15.1232 | 1.27E-03 | 3.62292 | 6.70448E-05 | 10.0681 | 7.40E-04 | 106.558 | 2.76E-03 | 528.757 | 1.75E-02 |
| 125 | 9.85 | 15.1254 | 1.28E-03 | 3.62279 | 6.72284E-05 | 10.0685 | 7.41E-04 | 106.565 | 2.76E-03 | 528.816 | 1.75E-02 |
| 130 | 10.24 | 15.1278 | 1.32E-03 | 3.62278 | 7.08373E-05 | 10.0691 | 7.67E-04 | 106.574 | 2.85E-03 | 528.907 | 1.82E-02 |
| 135 | 9.91 | 15.1301 | 1.28E-03 | 3.62264 | 6.73467E-05 | 10.0696 | 7.42E-04 | 106.579 | 2.76E-03 | 528.979 | 1.76E-02 |
| 140 | 9.83 | 15.1326 | 1.27E-03 | 3.62257 | 6.6822E-05 | 10.0701 | 7.35E-04 | 106.587 | 2.73E-03 | 529.062 | 1.74E-02 |
| 145 | 9.73 | 15.1355 | 1.25E-03 | 3.62248 | 6.63204E-05 | 10.0709 | 7.27E-04 | 106.596 | 2.70E-03 | 529.163 | 1.72E-02 |
| 150 | 9.63 | 15.1382 | 1.24E-03 | 3.62243 | 6.59163E-05 | 10.0715 | 7.20E-04 | 106.605 | 2.67E-03 | 529.262 | 1.71E-02 |
| 155 | 9.59 | 15.1408 | 1.24E-03 | 3.62231 | 6.57802E-05 | 10.0722 | 7.19E-04 | 106.613 | 2.67E-03 | 529.346 | 1.71E-02 |
| 160 | 9.51 | 15.1437 | 1.24E-03 | 3.62222 | 6.52053E-05 | 10.0729 | 7.17E-04 | 106.622 | 2.66E-03 | 529.449 | 1.70E-02 |
| 165 | 9.24 | 15.1468 | 1.20E-03 | 3.62215 | 6.32325E-05 | 10.0736 | 6.94E-04 | 106.631 | 2.57E-03 | 529.561 | 1.65E-02 |
| 170 | 9.11 | 15.1500 | 1.18E-03 | 3.62209 | 6.24568E-05 | 10.0744 | 6.85E-04 | 106.640 | 2.53E-03 | 529.676 | 1.63E-02 |
| 175 | 9.01 | 15.1532 | 1.17E-03 | 3.62203 | 6.16845E-05 | 10.0752 | 6.76E-04 | 106.650 | 2.50E-03 | 529.795 | 1.61E-02 |
| 180 | 8.78 | 15.1567 | 1.14E-03 | 3.62196 | 6.00774E-05 | 10.0762 | 6.59E-04 | 106.660 | 2.43E-03 | 529.930 | 1.57E-02 |
| 185 | 8.60 | 15.1599 | 1.11E-03 | 3.62186 | 5.8672E-05 | 10.0771 | 6.43E-04 | 106.671 | 2.37E-03 | 530.048 | 1.53E-02 |
| 190 | 8.39 | 15.1635 | 1.08E-03 | 3.62176 | 5.73485E-05 | 10.0781 | 6.26E-04 | 106.682 | 2.31E-03 | 530.180 | 1.49E-02 |
| 195 | 8.23 | 15.1673 | 1.06E-03 | 3.62163 | 5.64012E-05 | 10.0794 | 6.15E-04 | 106.694 | 2.26E-03 | 530.329 | 1.47E-02 |
| 200 | 7.82 | 15.1721 | 1.01E-03 | 3.62153 | 5.36663E-05 | 10.0808 | 5.85E-04 | 106.710 | 2.15E-03 | 530.511 | 1.40E-02 |
| 205 | 7.54 | 15.1774 | 9.72E-04 | 3.62139 | 5.1511E-05 | 10.0824 | 5.62E-04 | 106.728 | 2.06E-03 | 530.710 | 1.34E-02 |
| 210 | 7.29 | 15.1832 | 9.39E-04 | 3.62120 | 4.97669E-05 | 10.0841 | 5.43E-04 | 106.747 | 1.99E-03 | 530.923 | 1.30E-02 |
| 215 | 7.08 | 15.1912 | 9.10E-04 | 3.62103 | 4.8534E-05 | 10.0865 | 5.28E-04 | 106.776 | 1.93E-03 | 531.220 | 1.25E-02 |
| 220 | 6.91 | 15.1938 | 8.86E-04 | 3.62100 | 4.7297E-05 | 10.0872 | 5.15E-04 | 106.785 | 1.88E-03 | 531.318 | 1.22E-02 |
| 225 | 6.85 | 15.1962 | 8.80E-04 | 3.62096 | 4.69035E-05 | 10.0878 | 5.12E-04 | 106.794 | 1.87E-03 | 531.402 | 1.20E-02 |
| 230 | 6.84 | 15.1985 | 8.78E-04 | 3.62090 | 4.65611E-05 | 10.0884 | 5.12E-04 | 106.803 | 1.87E-03 | 531.483 | 1.20E-02 |
| 235 | 6.79 | 15.2017 | 8.75E-04 | 3.62083 | 4.64798E-05 | 10.0891 | 5.11E-04 | 106.815 | 1.86E-03 | 531.589 | 1.19E-02 |
| 240 | 6.75 | 15.2042 | 8.72E-04 | 3.62077 | 4.61947E-05 | 10.0898 | 5.10E-04 | 106.825 | 1.86E-03 | 531.677 | 1.18E-02 |
| 245 | 6.67 | 15.2070 | 8.62E-04 | 3.62068 | 4.56385E-05 | 10.0905 | 5.05E-04 | 106.835 | 1.84E-03 | 531.766 | 1.16E-02 |
| 250 | 6.65 | 15.2099 | 8.64E-04 | 3.62063 | 4.56371E-05 | 10.0912 | 5.07E-04 | 106.846 | 1.84E-03 | 531.869 | 1.15E-02 |
| 255 | 6.62 | 15.2125 | 8.64E-04 | 3.62057 | 4.5491E-05 | 10.0919 | 5.08E-04 | 106.857 | 1.84E-03 | 531.957 | 1.15E-02 |
| 260 | 6.56 | 15.2152 | 8.58E-04 | 3.62054 | 4.51535E-05 | 10.0926 | 5.05E-04 | 106.866 | 1.83E-03 | 532.059 | 1.14E-02 |
| 265 | 6.50 | 15.2177 | 8.50E-04 | 3.62048 | 4.46054E-05 | 10.0932 | 5.02E-04 | 106.876 | 1.82E-03 | 532.143 | 1.12E-02 |
| 270 | 6.48 | 15.2204 | 8.51E-04 | 3.62040 | 4.44205E-05 | 10.0939 | 5.04E-04 | 106.887 | 1.82E-03 | 532.232 | 1.12E-02 |
| 275 | 6.43 | 15.2230 | 8.47E-04 | 3.62036 | 4.43296E-05 | 10.0945 | 5.02E-04 | 106.896 | 1.82E-03 | 532.323 | 1.11E-02 |
| 280 | 6.41 | 15.2256 | 8.45E-04 | 3.62032 | 4.42601E-05 | 10.0952 | 5.01E-04 | 106.906 | 1.81E-03 | 532.414 | 1.10E-02 |
| 285 | 6.37 | 15.2279 | 8.41E-04 | 3.62028 | 4.3947E-05 | 10.0958 | 4.99E-04 | 106.916 | 1.80E-03 | 532.494 | 1.09E-02 |
| 290 | 6.30 | 15.2306 | 8.33E-04 | 3.62023 | 4.35395E-05 | 10.0965 | 4.95E-04 | 106.926 | 1.79E-03 | 532.590 | 1.08E-02 |
| 295 | 6.31 | 15.2330 | 8.35E-04 | 3.62015 | 4.36697E-05 | 10.0972 | 4.97E-04 | 106.935 | 1.79E-03 | 532.673 | 1.08E-02 |
| 300 | 6.30 | 15.2355 | 8.35E-04 | 3.62010 | 4.36093E-05 | 10.0979 | 4.97E-04 | 106.945 | 1.79E-03 | 532.760 | 1.08E-02 |
| 305 | 6.29 | 15.2380 | 8.35E-04 | 3.62005 | 4.35553E-05 | 10.0986 | 4.98E-04 | 106.955 | 1.79E-03 | 532.849 | 1.07E-02 |
| 310 | 6.24 | 15.2404 | 8.29E-04 | 3.62001 | 4.31425E-05 | 10.0993 | 4.94E-04 | 106.963 | 1.78E-03 | 532.940 | 1.06E-02 |
| 315 | 6.27 | 15.2419 | 8.32E-04 | 3.61997 | 4.33592E-05 | 10.0997 | 4.96E-04 | 106.969 | 1.78E-03 | 532.992 | 1.07E-02 |
| 320 | 6.20 | 15.2443 | 8.23E-04 | 3.61993 | 4.28576E-05 | 10.1002 | 4.90E-04 | 106.977 | 1.76E-03 | 533.073 | 1.05E-02 |
| 325 | 6.23 | 15.2466 | 8.28E-04 | 3.61987 | 4.32261E-05 | 10.1009 | 4.93E-04 | 106.986 | 1.77E-03 | 533.157 | 1.06E-02 |
| 330 | 6.25 | 15.2487 | 8.29E-04 | 3.61984 | 4.3493E-05 | 10.1015 | 4.94E-04 | 106.993 | 1.77E-03 | 533.236 | 1.06E-02 |
| 335 | 6.21 | 15.2508 | 8.23E-04 | 3.61978 | 4.3249E-05 | 10.1021 | 4.91E-04 | 107.001 | 1.76E-03 | 533.314 | 1.06E-02 |
| 340 | 6.22 | 15.2529 | 8.24E-04 | 3.61973 | 4.33414E-05 | 10.1027 | 4.91E-04 | 107.008 | 1.76E-03 | 533.385 | 1.06E-02 |
| 345 | 6.24 | 15.2551 | 8.28E-04 | 3.61970 | 4.35638E-05 | 10.1033 | 4.94E-04 | 107.017 | 1.77E-03 | 533.470 | 1.06E-02 |
| 350 | 6.22 | 15.2573 | 8.25E-04 | 3.61965 | 4.33897E-05 | 10.1039 | 4.92E-04 | 107.024 | 1.76E-03 | 533.547 | 1.06E-02 |
| 355 | 6.21 | 15.2593 | 8.24E-04 | 3.61962 | 4.33297E-05 | 10.1045 | 4.91E-04 | 107.032 | 1.76E-03 | 533.622 | 1.06E-02 |
| 360 | 6.17 | 15.2620 | 8.17E-04 | 3.61959 | 4.32243E-05 | 10.1053 | 4.87E-04 | 107.042 | 1.74E-03 | 533.726 | 1.05E-02 |
| 365 | 6.23 | 15.2633 | 8.25E-04 | 3.61955 | 4.36722E-05 | 10.1056 | 4.92E-04 | 107.047 | 1.76E-03 | 533.769 | 1.06E-02 |
| 370 | 6.18 | 15.2664 | 8.19E-04 | 3.61949 | 4.33987E-05 | 10.1066 | 4.89E-04 | 107.058 | 1.75E-03 | 533.885 | 1.05E-02 |
| 375 | 6.21 | 15.2679 | 8.23E-04 | 3.61943 | 4.37293E-05 | 10.1070 | 4.91E-04 | 107.064 | 1.75E-03 | 533.936 | 1.06E-02 |
| 380 | 6.23 | 15.2696 | 8.25E-04 | 3.61941 | 4.38822E-05 | 10.1075 | 4.93E-04 | 107.071 | 1.76E-03 | 533.998 | 1.06E-02 |
| 385 | 6.24 | 15.2717 | 8.25E-04 | 3.61937 | 4.40258E-05 | 10.1080 | 4.93E-04 | 107.078 | 1.76E-03 | 534.074 | 1.07E-02 |
| 390 | 6.16 | 15.2747 | 8.16E-04 | 3.61932 | 4.35759E-05 | 10.1090 | 4.88E-04 | 107.089 | 1.74E-03 | 534.189 | 1.06E-02 |
| 395 | 6.26 | 15.2746 | 8.30E-04 | 3.61931 | 4.441E-05 | 10.1090 | 4.97E-04 | 107.090 | 1.77E-03 | 534.187 | 1.07E-02 |

**Table S37.** Refined atomic positions, thermal parameters and occupancies for β′-$Cu_{0.283(16)}$/β-$Pb_{0.0131(5)}V_2O_5$

| Atom | Wyck. Pos. | *x* | *y* | *z* | Occ. | $U_{iso}$ (Å$^2$) |
|---|---|---|---|---|---|---|
| Cu(1) | 4*i* | 0.5406(11) | 0 | 0.3469(15) | 0.165(12) | 0.0057 |
| Cu(2) | 8*j* | 0.5247(8) | -0.113(4) | 0.3679(11) | 0.130(6) | 0.0043 |
| Pb(1) | 4*i* | 0.5192(30) | 0.5 | 0.427(3) | 0.0196(8) | 0.0453 |
| V(1) | 4*i* | 0.11448(17) | 0 | 0.12073(25) | 1 | 0.008 |
| V(2) | 4*i* | 0.33468(17) | 0 | 0.09425(24) | 1 | 0.0058 |
| V(3) | 4*i* | 0.28702(19) | 0 | 0.40724(26) | 1 | 0.006 |
| O(1) | 4*i* | 0.0947(6) | 0 | 0.2695(8) | 1 | 0.0394 |
| O(2) | 2*a* | 0 | 0 | 0 | 1 | 0.0008 |
| O(3) | 4*i* | 0.2646(6) | 0 | 0.2219(9) | 1 | 0.0473 |
| O(4) | 4*i* | 0.1870(5) | 0 | -0.0519(8) | 1 | 0.0241 |
| O(5) | 4*i* | 0.4307(5) | 0 | 0.2044(8) | 1 | 0.0397 |
| O(6) | 4*i* | 0.3653(6) | 0 | -0.0862(8) | 1 | 0.0253 |
| O(7) | 4*i* | 0.3947(6) | 0 | 0.4631(9) | 1 | 0.059 |
| O(8) | 4*i* | 0.2476(6) | 0 | 0.5772(7) | 1 | 0.018 |

| **Table S38.** Lattice paramters, unit cell volumes, standard deviations and refinement statistics for the sequential refinement of β'-$Cu_{0.33}$/β-$Pb_{0.05}V_2O_5$ | | | | | | | | | | | |
|---|---|---|---|---|---|---|---|---|---|---|---|
| Temp. (K) | Rwp (%) | *a* (Å) | std. dev. (Å) | *b* (Å) | std. dev. (Å) | *c* (Å) | std. dev (Å) | β (°) | std. dev. (°) | Vol (Å³) | std. dev. (Å³) |
| 90 | 12.44 | 15.1496 | 2.44E-03 | 3.62403 | 9.89051E-05 | 10.0733 | 1.49E-03 | 106.629 | 5.36E-03 | 529.919 | 3.24E-02 |
| 95 | 12.36 | 15.1517 | 2.39E-03 | 3.62398 | 9.79352E-05 | 10.0737 | 1.46E-03 | 106.635 | 5.25E-03 | 529.989 | 3.18E-02 |
| 100 | 12.29 | 15.1535 | 2.38E-03 | 3.62395 | 9.74832E-05 | 10.0740 | 1.45E-03 | 106.641 | 5.21E-03 | 530.049 | 3.16E-02 |
| 105 | 12.22 | 15.1553 | 2.36E-03 | 3.62390 | 9.65807E-05 | 10.0744 | 1.44E-03 | 106.646 | 5.16E-03 | 530.115 | 3.13E-02 |
| 110 | 12.30 | 15.1573 | 2.37E-03 | 3.62382 | 9.70203E-05 | 10.0748 | 1.45E-03 | 106.655 | 5.19E-03 | 530.165 | 3.15E-02 |
| 115 | 12.30 | 15.1592 | 2.37E-03 | 3.62376 | 9.70447E-05 | 10.0752 | 1.44E-03 | 106.662 | 5.19E-03 | 530.226 | 3.15E-02 |
| 120 | 12.14 | 15.1610 | 2.35E-03 | 3.62371 | 9.56171E-05 | 10.0756 | 1.43E-03 | 106.669 | 5.13E-03 | 530.286 | 3.11E-02 |
| 125 | 12.14 | 15.1628 | 2.35E-03 | 3.62366 | 9.54887E-05 | 10.0760 | 1.43E-03 | 106.677 | 5.12E-03 | 530.342 | 3.11E-02 |
| 130 | 12.22 | 15.1647 | 2.35E-03 | 3.62361 | 9.59511E-05 | 10.0765 | 1.43E-03 | 106.683 | 5.14E-03 | 530.409 | 3.12E-02 |
| 135 | 12.14 | 15.1666 | 2.34E-03 | 3.62356 | 9.51345E-05 | 10.0769 | 1.42E-03 | 106.690 | 5.10E-03 | 530.470 | 3.10E-02 |
| 140 | 12.12 | 15.1686 | 2.33E-03 | 3.62352 | 9.49976E-05 | 10.0775 | 1.42E-03 | 106.695 | 5.08E-03 | 530.546 | 3.08E-02 |
| 145 | 12.08 | 15.1703 | 2.31E-03 | 3.62347 | 9.44069E-05 | 10.0779 | 1.41E-03 | 106.701 | 5.04E-03 | 530.607 | 3.06E-02 |
| 150 | 11.99 | 15.1727 | 2.32E-03 | 3.62337 | 9.36393E-05 | 10.0788 | 1.41E-03 | 106.714 | 5.05E-03 | 530.682 | 3.06E-02 |
| 155 | 11.93 | 15.1743 | 2.29E-03 | 3.62335 | 9.27346E-05 | 10.0792 | 1.39E-03 | 106.716 | 4.98E-03 | 530.754 | 3.02E-02 |
| 160 | 11.91 | 15.1768 | 2.30E-03 | 3.62327 | 9.22692E-05 | 10.0800 | 1.40E-03 | 106.728 | 5.00E-03 | 530.837 | 3.03E-02 |
| 165 | 11.76 | 15.1788 | 2.26E-03 | 3.62322 | 9.10884E-05 | 10.0804 | 1.37E-03 | 106.732 | 4.90E-03 | 530.915 | 2.97E-02 |
| 170 | 11.70 | 15.1814 | 2.25E-03 | 3.62316 | 9.07728E-05 | 10.0812 | 1.36E-03 | 106.741 | 4.88E-03 | 531.011 | 2.95E-02 |
| 175 | 11.65 | 15.1840 | 2.24E-03 | 3.62310 | 9.04382E-05 | 10.0819 | 1.36E-03 | 106.749 | 4.88E-03 | 531.107 | 2.95E-02 |
| 180 | 11.58 | 15.1869 | 2.23E-03 | 3.62301 | 8.98919E-05 | 10.0828 | 1.35E-03 | 106.760 | 4.84E-03 | 531.213 | 2.92E-02 |
| 185 | 11.50 | 15.1899 | 2.22E-03 | 3.62291 | 8.88926E-05 | 10.0837 | 1.35E-03 | 106.770 | 4.81E-03 | 531.320 | 2.89E-02 |
| 190 | 11.41 | 15.1927 | 2.19E-03 | 3.62286 | 8.81315E-05 | 10.0846 | 1.33E-03 | 106.780 | 4.76E-03 | 531.433 | 2.86E-02 |
| 195 | 11.36 | 15.1961 | 2.20E-03 | 3.62277 | 8.77547E-05 | 10.0855 | 1.34E-03 | 106.792 | 4.77E-03 | 531.552 | 2.85E-02 |
| 200 | 11.25 | 15.1993 | 2.18E-03 | 3.62270 | 8.64777E-05 | 10.0863 | 1.33E-03 | 106.803 | 4.74E-03 | 531.663 | 2.82E-02 |
| 205 | 11.16 | 15.2027 | 2.16E-03 | 3.62261 | 8.59518E-05 | 10.0872 | 1.32E-03 | 106.814 | 4.70E-03 | 531.788 | 2.79E-02 |
| 210 | 11.05 | 15.2055 | 2.14E-03 | 3.62257 | 8.50959E-05 | 10.0880 | 1.31E-03 | 106.824 | 4.66E-03 | 531.893 | 2.75E-02 |
| 215 | 10.95 | 15.2083 | 2.13E-03 | 3.62253 | 8.43057E-05 | 10.0886 | 1.30E-03 | 106.834 | 4.63E-03 | 531.988 | 2.72E-02 |
| 220 | 10.90 | 15.2135 | 2.13E-03 | 3.62247 | 8.37999E-05 | 10.0899 | 1.31E-03 | 106.854 | 4.64E-03 | 532.174 | 2.69E-02 |
| 225 | 10.84 | 15.2153 | 2.13E-03 | 3.62244 | 8.37219E-05 | 10.0903 | 1.31E-03 | 106.861 | 4.64E-03 | 532.232 | 2.68E-02 |
| 230 | 10.83 | 15.2171 | 2.13E-03 | 3.62238 | 8.36321E-05 | 10.0906 | 1.31E-03 | 106.868 | 4.64E-03 | 532.287 | 2.67E-02 |
| 235 | 10.72 | 15.2195 | 2.11E-03 | 3.62236 | 8.24871E-05 | 10.0911 | 1.30E-03 | 106.876 | 4.60E-03 | 532.371 | 2.64E-02 |
| 240 | 10.66 | 15.2221 | 2.10E-03 | 3.62233 | 8.2278E-05 | 10.0916 | 1.29E-03 | 106.886 | 4.58E-03 | 532.457 | 2.62E-02 |
| 245 | 10.59 | 15.2245 | 2.09E-03 | 3.62228 | 8.17984E-05 | 10.0923 | 1.29E-03 | 106.896 | 4.56E-03 | 532.540 | 2.60E-02 |
| 250 | 10.47 | 15.2272 | 2.06E-03 | 3.62219 | 8.11396E-05 | 10.0930 | 1.28E-03 | 106.905 | 4.51E-03 | 532.632 | 2.56E-02 |
| 255 | 10.36 | 15.2297 | 2.04E-03 | 3.62214 | 8.0109E-05 | 10.0937 | 1.26E-03 | 106.915 | 4.46E-03 | 532.719 | 2.52E-02 |
| 260 | 10.33 | 15.2319 | 2.03E-03 | 3.62212 | 8.02793E-05 | 10.0941 | 1.26E-03 | 106.922 | 4.46E-03 | 532.794 | 2.52E-02 |
| 265 | 10.30 | 15.2346 | 2.02E-03 | 3.62206 | 8.01559E-05 | 10.0946 | 1.26E-03 | 106.932 | 4.44E-03 | 532.879 | 2.50E-02 |
| 270 | 10.24 | 15.2370 | 2.01E-03 | 3.62198 | 7.9508E-05 | 10.0952 | 1.25E-03 | 106.941 | 4.41E-03 | 532.955 | 2.48E-02 |
| 275 | 10.16 | 15.2394 | 2.00E-03 | 3.62194 | 7.9015E-05 | 10.0958 | 1.24E-03 | 106.950 | 4.38E-03 | 533.043 | 2.46E-02 |
| 280 | 10.13 | 15.2417 | 1.99E-03 | 3.62191 | 7.84102E-05 | 10.0964 | 1.24E-03 | 106.959 | 4.36E-03 | 533.125 | 2.44E-02 |
| 285 | 10.06 | 15.2441 | 1.97E-03 | 3.62187 | 7.80269E-05 | 10.0970 | 1.23E-03 | 106.968 | 4.32E-03 | 533.212 | 2.42E-02 |
| 290 | 10.01 | 15.2465 | 1.96E-03 | 3.62185 | 7.77633E-05 | 10.0976 | 1.22E-03 | 106.976 | 4.29E-03 | 533.298 | 2.40E-02 |
| 295 | 9.96 | 15.2487 | 1.94E-03 | 3.62182 | 7.75431E-05 | 10.0981 | 1.21E-03 | 106.984 | 4.25E-03 | 533.378 | 2.38E-02 |
| 300 | 9.84 | 15.2510 | 1.92E-03 | 3.62178 | 7.6709E-05 | 10.0988 | 1.19E-03 | 106.992 | 4.20E-03 | 533.463 | 2.35E-02 |
| 305 | 9.74 | 15.2534 | 1.89E-03 | 3.62173 | 7.61152E-05 | 10.0992 | 1.18E-03 | 107.000 | 4.15E-03 | 533.540 | 2.32E-02 |
| 310 | 9.66 | 15.2559 | 1.87E-03 | 3.62169 | 7.57013E-05 | 10.0999 | 1.17E-03 | 107.007 | 4.10E-03 | 533.635 | 2.29E-02 |
| 315 | 9.74 | 15.2572 | 1.90E-03 | 3.62168 | 7.63171E-05 | 10.1004 | 1.19E-03 | 107.014 | 4.16E-03 | 533.689 | 2.33E-02 |
| 320 | 9.70 | 15.2597 | 1.89E-03 | 3.62165 | 7.63349E-05 | 10.1010 | 1.18E-03 | 107.023 | 4.15E-03 | 533.778 | 2.32E-02 |
| 325 | 9.64 | 15.2617 | 1.88E-03 | 3.62163 | 7.57723E-05 | 10.1016 | 1.17E-03 | 107.030 | 4.12E-03 | 533.856 | 2.31E-02 |
| 330 | 9.60 | 15.2638 | 1.87E-03 | 3.62160 | 7.54422E-05 | 10.1022 | 1.17E-03 | 107.037 | 4.09E-03 | 533.937 | 2.29E-02 |
| 335 | 9.54 | 15.2660 | 1.85E-03 | 3.62158 | 7.52355E-05 | 10.1028 | 1.16E-03 | 107.043 | 4.05E-03 | 534.022 | 2.27E-02 |
| 340 | 9.51 | 15.2680 | 1.84E-03 | 3.62154 | 7.51091E-05 | 10.1033 | 1.15E-03 | 107.050 | 4.02E-03 | 534.094 | 2.26E-02 |
| 345 | 9.49 | 15.2702 | 1.84E-03 | 3.62150 | 7.51336E-05 | 10.1039 | 1.15E-03 | 107.057 | 4.01E-03 | 534.178 | 2.26E-02 |
| 350 | 9.44 | 15.2726 | 1.82E-03 | 3.62144 | 7.48139E-05 | 10.1045 | 1.14E-03 | 107.065 | 3.99E-03 | 534.262 | 2.24E-02 |
| 355 | 9.41 | 15.2746 | 1.82E-03 | 3.62143 | 7.48047E-05 | 10.1050 | 1.14E-03 | 107.072 | 3.97E-03 | 534.337 | 2.24E-02 |
| 360 | 9.34 | 15.2769 | 1.80E-03 | 3.62139 | 7.43713E-05 | 10.1057 | 1.13E-03 | 107.081 | 3.94E-03 | 534.421 | 2.22E-02 |
| 365 | 9.35 | 15.2788 | 1.80E-03 | 3.62136 | 7.45439E-05 | 10.1062 | 1.13E-03 | 107.087 | 3.94E-03 | 534.497 | 2.22E-02 |
| 370 | 9.26 | 15.2813 | 1.78E-03 | 3.62131 | 7.40081E-05 | 10.1069 | 1.12E-03 | 107.094 | 3.89E-03 | 534.588 | 2.20E-02 |
| 375 | 9.19 | 15.2837 | 1.76E-03 | 3.62129 | 7.36038E-05 | 10.1076 | 1.10E-03 | 107.102 | 3.85E-03 | 534.686 | 2.18E-02 |
| 380 | 9.20 | 15.2852 | 1.77E-03 | 3.62127 | 7.37736E-05 | 10.1079 | 1.11E-03 | 107.107 | 3.86E-03 | 534.740 | 2.19E-02 |
| 385 | 9.12 | 15.2881 | 1.75E-03 | 3.62120 | 7.34673E-05 | 10.1087 | 1.10E-03 | 107.117 | 3.83E-03 | 534.844 | 2.17E-02 |
| 390 | 9.10 | 15.2895 | 1.75E-03 | 3.62117 | 7.34721E-05 | 10.1091 | 1.10E-03 | 107.122 | 3.82E-03 | 534.894 | 2.17E-02 |
| 395 | 9.05 | 15.2904 | 1.74E-03 | 3.62115 | 7.31004E-05 | 10.1093 | 1.09E-03 | 107.124 | 3.80E-03 | 534.928 | 2.16E-02 |

**Table S39.** Refined atomic positions, thermal parameters and occupancies for β’-$Cu_{0.259(13)}$/β-$Pb_{0.040(2)}V_2O_5$

| Atom | Wyck. Pos. | *x* | *y* | *z* | Occ. | $U_{iso}$ (Å$^2$) |
|---|---|---|---|---|---|---|
| Cu(1) | 4*i* | 0.55789 | 0 | 0.32404 | 0.104(12) | 0.053(21) |
| Cu(2) | 8*j* | 0.5286(10) | -0.080(4) | 0.3627(14) | 0.142(4) | 0.01 |
| Pb(1) | 4*i* | 0.5263(29) | 0.5 | 0.417(3) | 0.060(3) | 0.148(21) |
| V(1) | 4*i* | 0.1149(3) | 0 | 0.1206(5) | 1 | 0.0169(16) |
| V(2) | 4*i* | 0.3376(4) | 0 | 0.0966(5) | 1 | 0.0172(16) |
| V(3) | 4*i* | 0.2878(4) | 0 | 0.4058(6) | 1 | 0.0163(15) |
| O(1) | 4*i* | 0.0799(14) | 0 | 0.2682(20) | 1 | 0.094(8) |
| O(2) | 2*a* | 0 | 0 | 0 | 1 | 0.0178 |
| O(3) | 4*i* | 0.2671(9) | 0 | 0.2238(13) | 1 | 0.001(4) |
| O(4) | 4*i* | 0.1874(11) | 0 | -0.0473(18) | 1 | 0.044(6) |
| O(5) | 4*i* | 0.4328(11) | 0 | 0.1985(17) | 1 | 0.058(7) |
| O(6) | 4*i* | 0.3589(10) | 0 | -0.0785(15) | 1 | 0.019(4) |
| O(7) | 4*i* | 0.3888(12) | 0 | 0.4685(18) | 1 | 0.053(7) |
| O(8) | 4*i* | 0.2448(11) | 0 | 0.5766(13) | 1 | 0.015(5) |

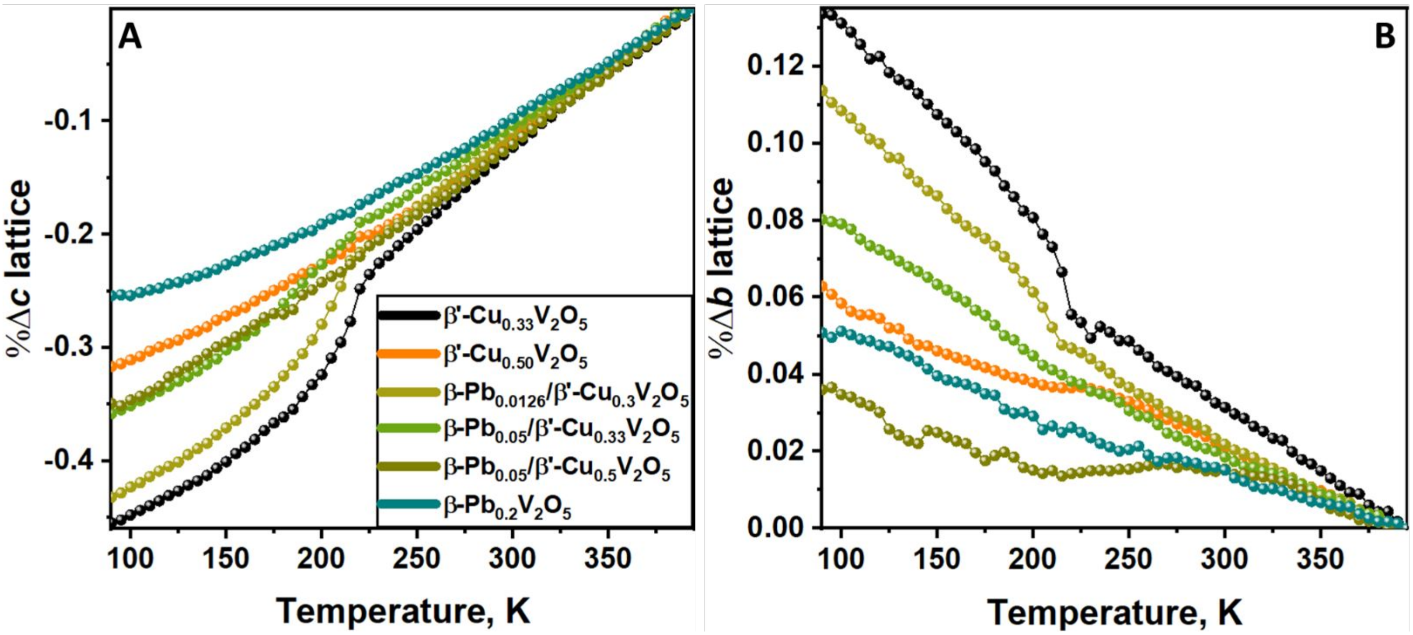


***Figure S13**. **Structural origins of conductance nonlinearities in single crystals of β-$Pb_x$/β'-$Cu_yV_2O_5$.*** Evolution of **(A)** the c lattice parameter, and **(B)** b lattice parameter as a function of temperature for β'-$Cu_{0.33}V_2O_5$, β'-$Cu_{0.50}V_2O_5$, β-$Pb_{0.0126}$/β'-$Cu_{0.3}V_2O_5$, β-$Pb_{0.05}$/β'-$Cu_{0.33}V_2O_5$, β-$Pb_{0.05}$/β'-$Cu_{0.5}V_2O_5$ and β-$Pb_xV_2O_5$.

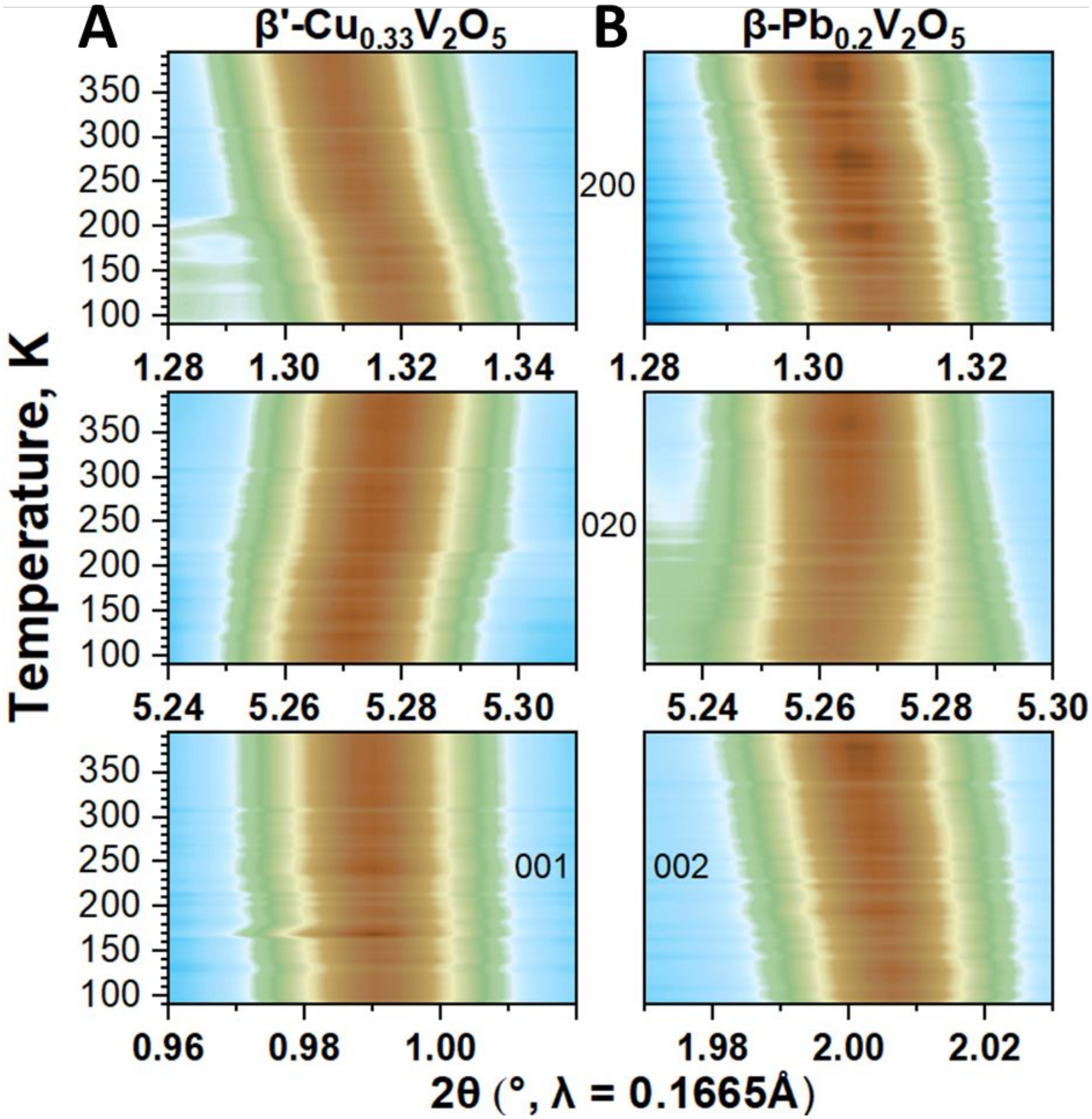


***Figure S14. Evolution of lattice parameters of end-member $M_xV_2O_5$ (M = Pb, Cu)***. Synchrotron X-ray diffraction contour color plot of a selected 2θ region, as a function of temperature (90-395K) collected using a ramp rate of 1K for end-members **(A)** β'-$Cu_{033}V_2O_5$ and **(B)** β-$Pb_{0.2}V_2O_5$.

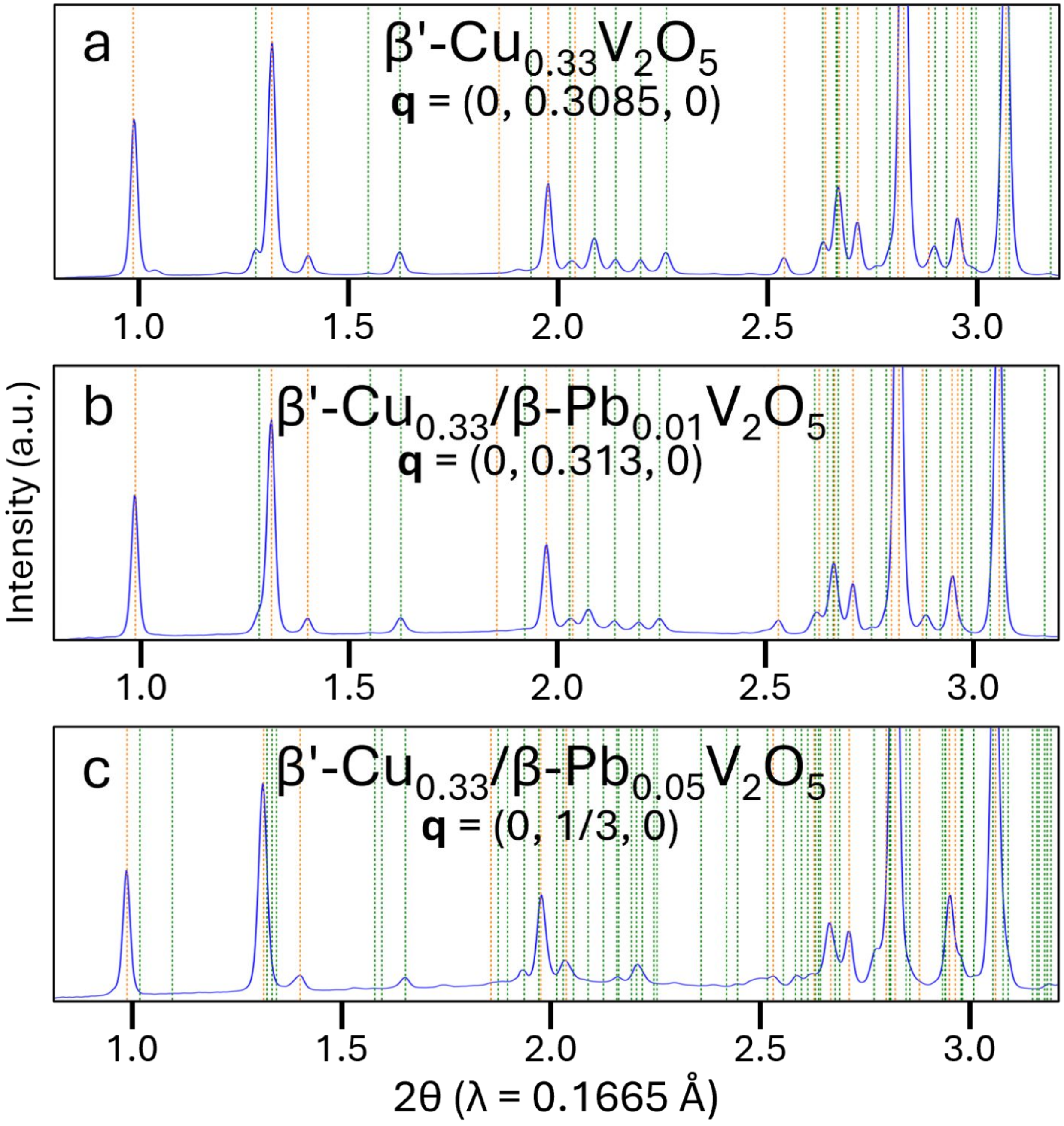


***Figure S15.*** Indexing low-temperature structure modulations. Several samples exhibit structural modulations (assigned by a modulation vector **q**), evidenced by the appearance of satellites (green) between parent reflections (orange). **(a)** β'-$Cu_{0.33}V_2O_5$ at 90K and **(b)** β'-$Cu_{0.33}$/β-$Pb_{0.01}V_2O_5$ at 115K, both show primarily first-order incommensurate modulations. **(c)** β'-$Cu_{0.33}$/β-$Pb_{0.05}V_2O_5$ at 90K, showing up to third-order commensurate modulation (supercell) satellites. As seen in **Figure S12**, β'-$Cu_{0.50}V_2O_5$ also exhibits modulation satellites below 215K but which are weak and too sparse in number for reliable indexing.

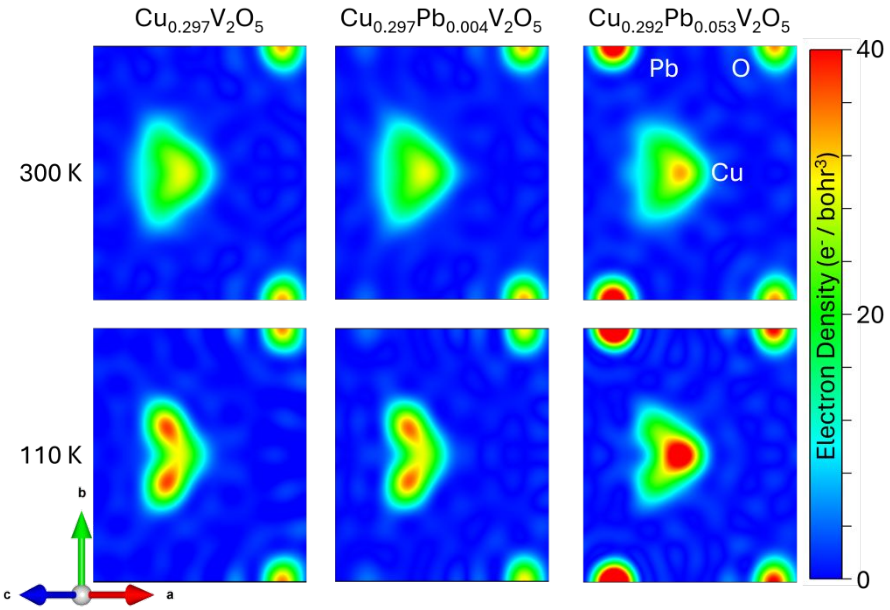


***Figure S16:*** Electron density maps in the plane containing Cu(1) and Cu(2) sites for several β-$Pb_x$/β'-$Cu_yV_2O_5$ compositions . In β'-$Cu_{0.297}V_2O_5$, Cu occupies the split sites preferentially at low temperature. Pb co-insertion suppresses this preference, likely by interrupting Cu interactions along the crystallographic b direction.

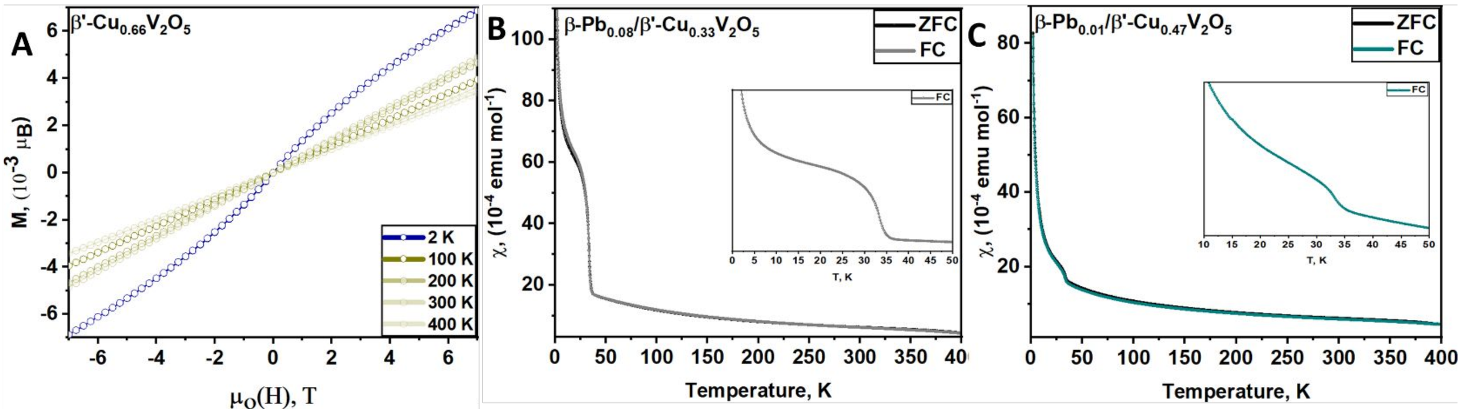


***Figure S17. Magnetic transitions in β-$Pb_x$/β'-$Cu_yV_2O_5$.*** **(A)** Magnetization versus magnetic field curve plotted at varied temperatures for β'-$Cu_{0.66}V_2O_5$, under an applied magnetic field ranging from –7 to +7 T. Temperature dependence of magnetic susceptibility of **(B)** β-$Pb_{0.08}$/β'-$Cu_{0.33}V_2O_5$, and **(C)** β-$Pb_{0.01}$/β'-$Cu_{0.47}V_2O_5$ between 2–400K at an applied field of 0.1 T. The positive value of Θ further corroborates predominantly ferromagnetic exchange interactions at 0.1 T between nearest-neighbor $V^{n+}$ ions (**Figure S18D-F**).

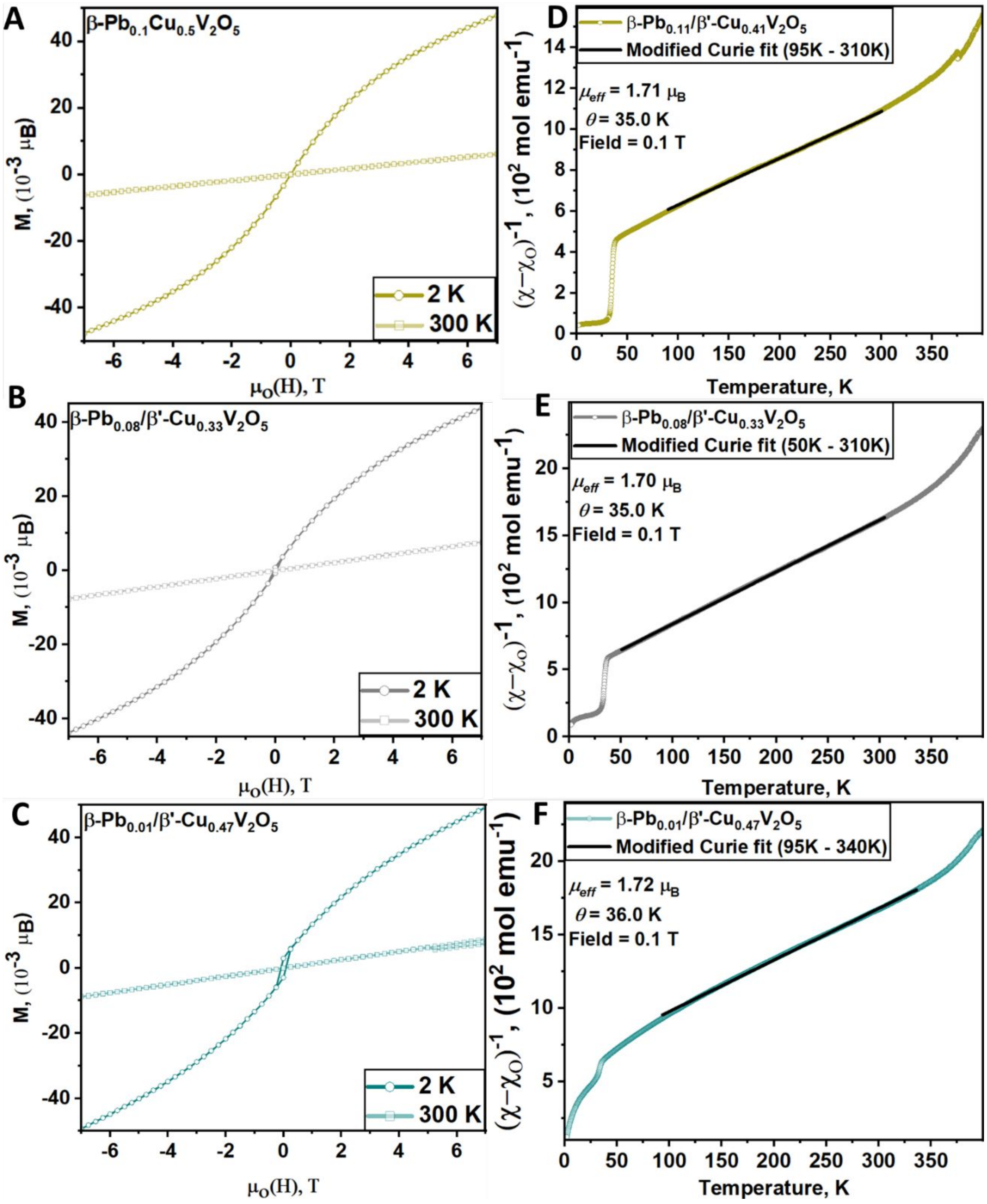


***Figure S18. Magnetization and inverse magnetic susceptibility in β-$Pb_X$/β'-$Cu_YV_2O_5$.*** Magnetization versus magnetic field curve plotted at 2 and 300 K for **(A)** β-$Pb_{0.11}$/β'-$Cu_{0.41}V_2O_5$, **(B)** β-$Pb_{0.08}$/β'-$Cu_{0.33}V_2O_5$, and **(C)** β-$Pb_{0.01}$/β'-$Cu_{0.47}V_2O_5$ under an applied magnetic field ranging from –7 to +7 T. Inverse magnetic susceptibility plot in the field-cooled state and results of the Curie−Weiss fitting for **(D)** β-$Pb_{0.11}$/β'-$Cu_{0.41}V_2O_5$, **(E)** β-$Pb_{0.08}$/β'-$Cu_{0.33}V_2O_5$, and **(F)** β-$Pb_{0.01}$/β'-$Cu_{0.47}V_2O_5$. The inverse magnetic susceptibility has been fit to the modified Curie−Weiss law; the relevant fit parameters obtained are presented within the panel. In A—C, all three compounds exhibit sharp magnetic switching at low field, and then undergo an abrupt metamagnetic transition with increasing applied fields, reaching a maximum value of the magnetization of ∼0.045 μB/fu at 7 T. Based on fittings (**Figure D-F**) to the modified Curie−Weiss law ($\chi = \chi_0 + C/(T-\vartheta)$), where $\Theta$ is the Weiss temperature and C is the Curie constant, Weiss constants $\Theta$ of 35, 35, and 36 K, with derived effective moment ( $\mu_{eff}$) of 1.71, 1.70, and 1.72 $\mu_B$ are measured for β-$Pb_{0.01}$/β'-$Cu_{0.47}V_2O_5$, β-$Pb_{0.08}$/β'-$Cu_{0.33}V_2O_5$, and β-

$Pb_{0.11}$/β'-$Cu_{0.41}V_2O_5$ respectively, which are close to the expected value for tetravalent vanadium (*S* = ½ ; $\mu_{eff}$ = 1.73 $\mu_B$). Evidence of ferromagnetic ordering of V $3d^1$ centers comes from measurements of magnetization as a function of magnetic field (**Figure S18A-C**) where the magnetization curve at 2K under an applied magnetic field ranging from –7 to +7 T displays a S-shaped profile. Stabilization of a ferromagnetic insulating state at 35K in β-$Pb_x$/β'-$Cu_yV_2O_5$ can be ascribed to shorter V—V bond distances enforced by local structure distortions arising from the presence of the $6s^2$ lone-pairs of $Pb^{2+}$ cations. The off-centering induced by Pb $6s^2$ lone pair states (**Figure 2**) elongates a pair of Pb—O bonds, concurrently strengthening Pb—O interactions on the opposite side of the interstitial cation site. The directional off-centering of Pb ions weakens V—O interactions, bringing adjacent vanadium atoms into closer proximity (**Figure 4J**).

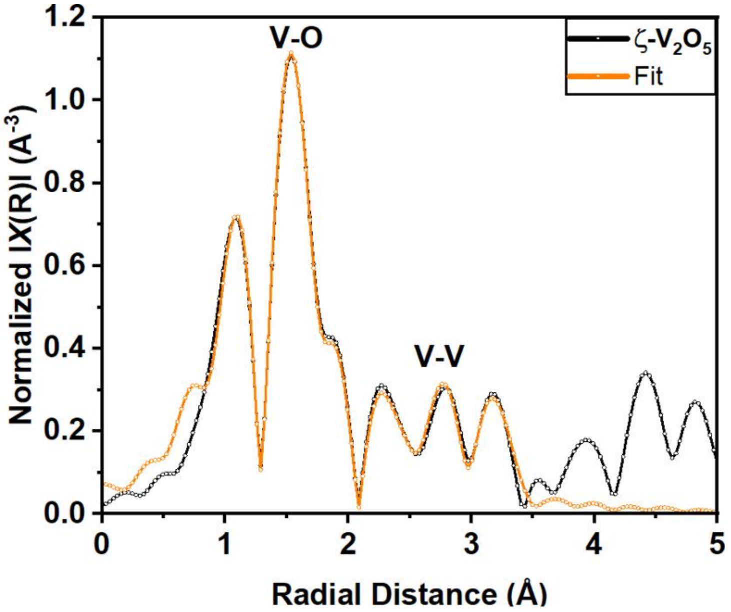


***Figure S19. Fitting results of the V K-edge EXAFS spectra of*** **ζ-$V_2O_5$.** The fitted R-space V K-edge EXAFS spectra of ζ-$V_2O_5$.

**Table S40. Tabulated V K-edge EXAFS fitting parameters of β-$Pb_x$/β'-$Cu_yV_2O_5$.** Atomic positions obtained from high-resolution single-crystal X-ray diffraction (Tables S13-S38) were used as initial models to fit the EXAFS data.

| Sample | Path | N | R(Å) | $\sigma^2$(Å)$^2$ | $S_0^2$ | $E_0$ (eV) | R-factor (%) |
|---|---|---|---|---|---|---|---|
| **ζ-$V_2O_5$** | V=O | 1 | 1.556 | 0.004 | 1.0 | 4.101 ± 1.011 | 0.3 |
| | V—$O_{trans}$ | 1 | 2.512 | | | | |
| | V—V | 2 | 3.025 | | | | |
| **β-$Pb_{0.01}$/β'-$Cu_{0.47}V_2O_5$** | V=O | 1 | 1.598 | 0.001 | 1.0 | 1.076 ± 0.072 | 0.7 |
| | V—$O_{trans}$ | 1 | 2.298 | | | | |
| | V—V | 2 | 2.961 | | | | |
| **β-$Pb_{0.08}$/β'-$Cu_{0.33}V_2O_5$** | V=O | 1 | 1.626 | 0.006 | 1.0 | 2.240 ± 0.217 | 0.9 |
| | V—$O_{trans}$ | 1 | 2.285 | | | | |
| | V—V | 2 | 2.964 | | | | |
| **β-$Pb_{0.11}$/β'-$Cu_{0.41}V_2O_5$** | V=O | 1 | 1.597 | 0.010 | 1.0 | 1.457 ± 3.275 | 0.5 |
| | V—$O_{trans}$ | 1 | 2.289 | | | | |
| | V—V | 2 | 2.944 | | | | |

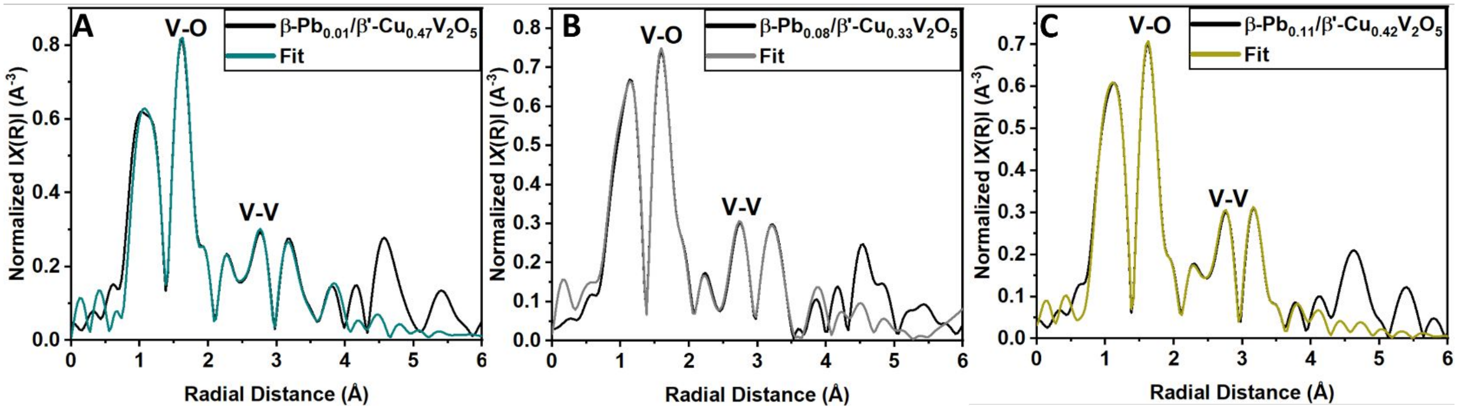


***Figure S20. Fitting results of the V K-edge EXAFS spectra of β-Pb$_x$/β'-Cu$_y$V$_2$O$_5$.*** The fitted R-space V K-edge EXAFS spectra for **(A)** β-Pb$_{0.01}$/β'-Cu$_{0.47}$V$_2$O$_5$; **( B)** β-Pb$_{0.08}$/β'-Cu$_{0.33}$V$_2$O$_5$; and **(C)** β-Pb$_{0.11}$/β'-Cu$_{0.41}$V$_2$O$_5$.

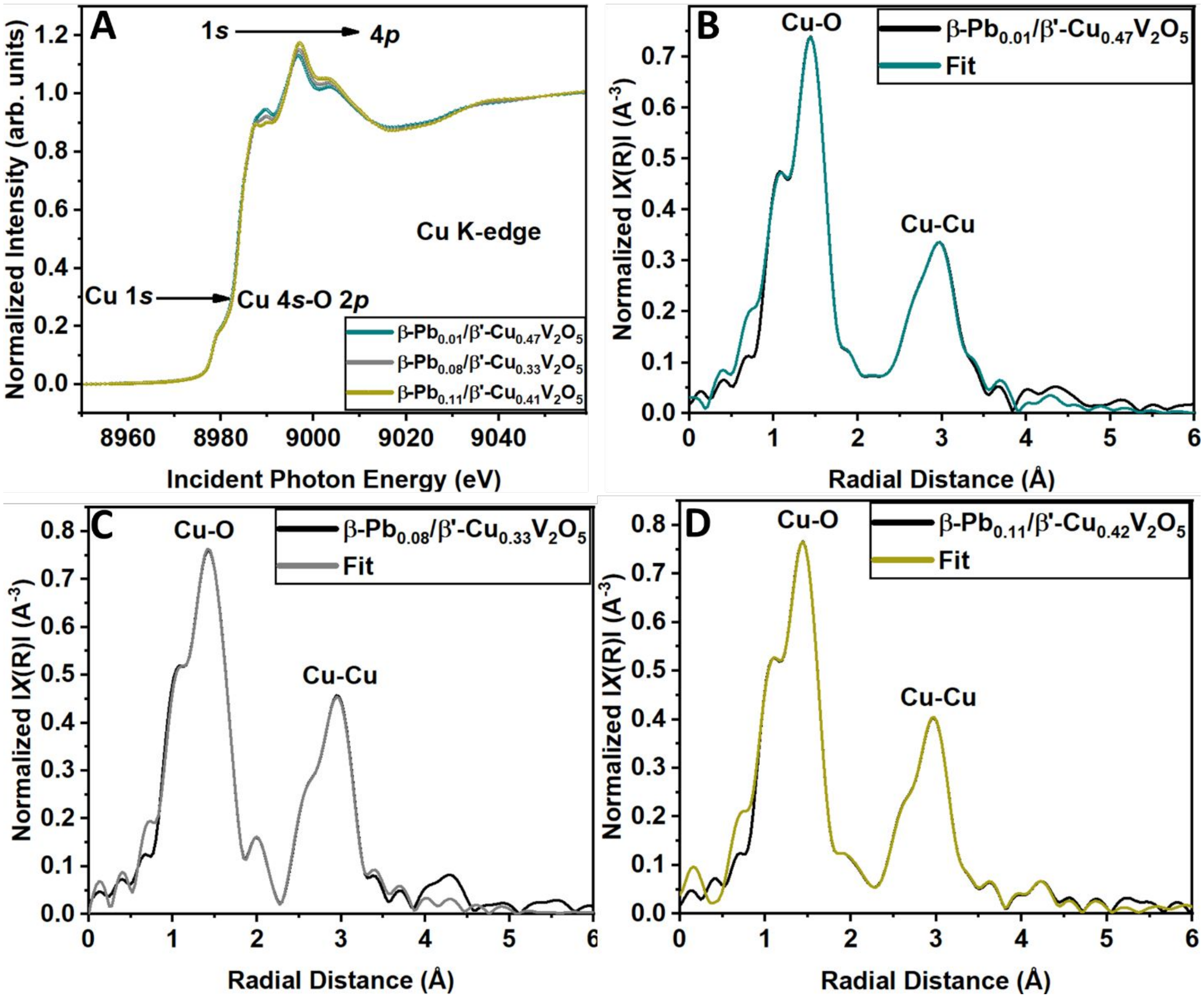


***Figure S21**. **Cu K-edge XANES and Fitting results of the Cu K-edge EXAFS spectra of β-Pb$_x$/β'-Cu$_y$V$_2$O$_5$.*** **(A)** Cu K-edge XANES spectra of β-Pb$_x$/β'-Cu$_y$V$_2$O$_5$. β-Pb$_{0.08}$/β'-Cu$_{0.33}$V$_2$O$_5$, β-Pb$_{0.11}$/β'-Cu$_{0.41}$V$_2$O$_5$ and β-Pb$_{0.01}$/β'-Cu$_{0.47}$V$_2$O$_5$. The rising edge centered at ca 8,979.4 eV derives from transitions from Cu 1s core levels to 4s states hybridized with O 2p states; whereas the white-line absorption centered at approximately 8,997.2 eV is ascribed to dipole-allowed Cu 1s → 4p transitions. Fitted R-space Cu K-edge EXAFS spectra for **(B)** β-Pb$_{0.01}$/β'-Cu$_{0.47}$V$_2$O$_5$ **( C)** β-Pb$_{0.08}$/β'-Cu$_{0.33}$V$_2$O$_5$ and **(D)** β-Pb$_{0.11}$/β'-Cu$_{0.41}$V$_2$O$_5$. The Cu—O fitting results for Cu(1)−O bond lengths are 1.91 and 2.86Å for β'-Cu$_{0.45}$V$_2$O$_5$;[2]

1.92 and 2.84Å for β-$Pb_{0.01}$/β'-$Cu_{0.47}V_2O_5$; 1.94 and 2.82Å for β-$Pb_{0.08}$/β'-$Cu_{0.33}V_2O_5$; and 1.95 and 2.81Å) for β-$Pb_{0.11}$/β'-$Cu_{0.41}V_2O_5$.

**Table S41. Tabulated Cu K-edge EXAFS fitting parameters of β-$Pb_x$/β'-$Cu_yV_2O_5$.** Atomic positions obtained from high-resolution single-crystal X-ray diffraction (Tables S13-S38) were used as initial models to fit the EXAFS data.

| Sample | Path | N | R(Å) | σ²(Å)² | $S_0^2$ | $E_0$ (eV) | R-factor (%) |
|---|---|---|---|---|---|---|---|
| **β'-$Cu_{0.45}V_2O_5$** | Cu(1)—O(4.1) | 1 | 1.91 | | 0.28 | | |
| | Cu(1)—O(8.1) | 1 | 1.78 | | | | |
| | Cu(1)—O(6.1) | 2 | 2.00 | | | | |
| | Cu(1)—O(8.2) | 1 | 2.50 | | | | |
| | Cu(1)—O(3.1) | 1 | 2.82 | | | | |
| | Cu(1)—O(7.1) | 1 | 2.86 | | | | |
| | Cu(2)—O(4.1) | 2 | 1.92 | | 0.79 | -0.673 ± 1.519 | 2.9 |
| | Cu(2)—O(6.1) | 1 | 2.08 | | | | |
| | Cu(2)—O(8.2) | 1 | 2.33 | | | | |
| | Cu(2)—O(6.2) | 1 | 2.57 | | | | |
| | Cu(2)—V(3.1) | 1 | 2.99 | | | | |
| | Cu(2)—Cu(2) | 2 | 3.00 | | 0.13 | | |
| **β-$Pb_{0.01}$/β'-$Cu_{0.47}V_2O_5$** | Cu(1)—O(4.1) | 1 | 1.923 | | | | |
| | Cu(1)—O(2.1) | 1 | 1.625 | | | | |
| | Cu(1)—O(8.1) | 2 | 2.259 | | | | |
| | Cu(1)—O(1.1) | 1 | 2.714 | 0.004 | | 3.543 ± 2.284 | |
| | Cu(1)—O(2.2) | 1 | 2.807 | | | | |
| | Cu(1)—O(7.1) | 1 | 2.841 | | | | |
| | Cu(2)—O(4.1) | 1 | 1.930 | | 1.00 | | 0.2 |
| | Cu(2)—O(2.1) | 1 | 2.145 | | | | |
| | Cu(2)—O(8.1) | 1 | 2.454 | | | | |
| | Cu(2)—O(8.2) | 1 | 2.645 | 0.003 | | 7.855 ± 1.056 | |
| | Cu(2)—O(2.2) | 1 | 2.962 | | | | |
| | Cu(2)—V(1.1) | 1 | 2.983 | | | | |
| **β-$Pb_{0.08}$/β'-$Cu_{0.33}V_2O_5$** | Cu(1)—O(4.1) | 1 | 1.937 | | | | |
| | Cu(1)—O(2.1) | 1 | 1.672 | | | | |
| | Cu(1)—O(8.1) | 2 | 2.105 | | | | |
| | Cu(1)—O(1.1) | 1 | 2.398 | | | | |
| | Cu(1)—O(2.2) | 1 | 2.791 | 0.005 | | 9.380 ± 0.634 | 0.1 |
| | Cu(1)—O(7.1) | 1 | 2.822 | | | | |
| | Cu(2)—O(4.1) | 1 | 1.939 | | 1.00 | | |
| | Cu(2)—O(2.1) | 1 | 2.137 | | | | |
| | Cu(2)—O(8.1) | 1 | 2.428 | | | | |
| | Cu(2)—O(8.2) | 1 | 2.695 | 0.004 | | | 0.2 |
| | Cu(2)—O(2.2) | 1 | 2.961 | | | 10.401 ± 5.635 | |
| | Cu(2)—V(1.1) | 1 | 2.981 | | | | |
| **β-$Pb_{0.11}$/β'-$Cu_{0.41}V_2O_5$** | Cu(1)—O(4.1) | 1 | 1.951 | | | | |
| | Cu(1)—O(2.1) | 1 | 1.693 | | | | |
| | Cu(1)—O(8.1) | 2 | 2.127 | | | | |
| | Cu(1)—O(1.1) | 1 | 2.427 | 0.002 | | 12.382 ± 1.713 | 0.4 |
| | Cu(1)—O(2.2) | 1 | 2.747 | | | | |
| | Cu(1)—O(7.1) | 1 | 2.814 | | | | |

| | | | | | | | |
|---|---|---|---|---|---|---|---|
| | Cu(2)—O(4.1) | 1 | 1.945 | 0.002 | 1.00 | 5.479 ± 2.787 | 0.1 |
| | Cu(2)—O(2.1) | 1 | 2.048 | | | | |
| | Cu(2)—O(8.1) | 1 | 2.273 | | | | |
| | Cu(2)—O(8.2) | 1 | 2.398 | | | | |
| | Cu(2)—O(2.2) | 1 | 2.954 | | | | |
| | Cu(2)—V(1.1) | 1 | 2.991 | | | | |

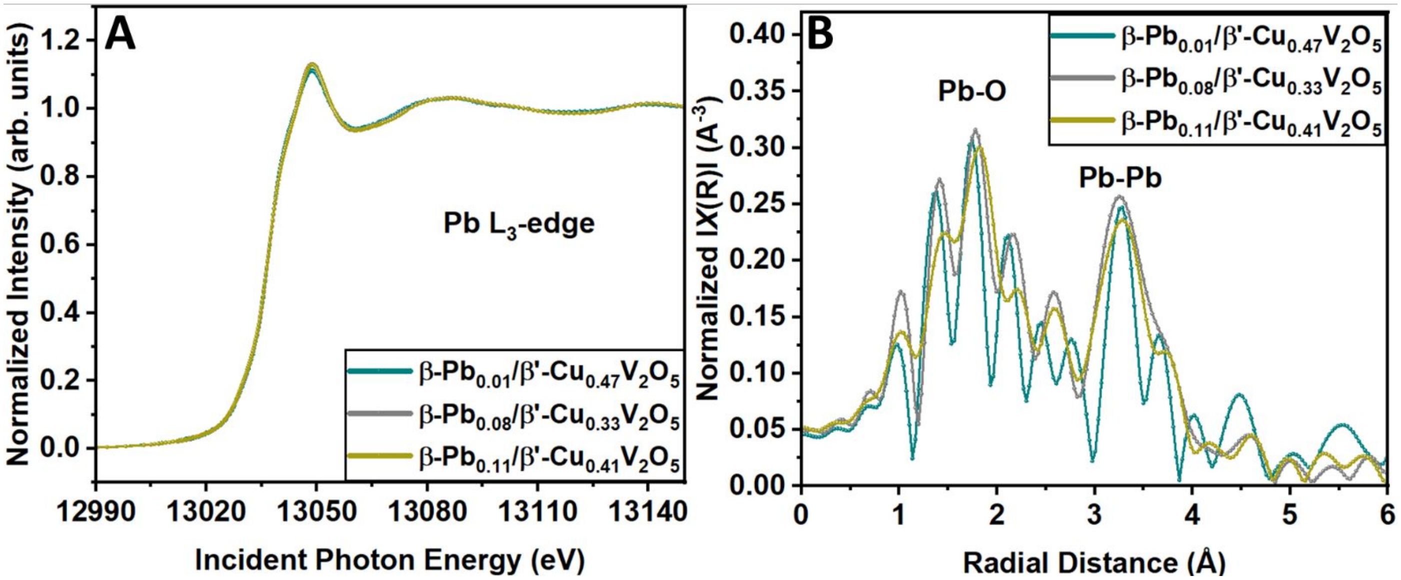


***Figure S22. Pb $L_3$-edge X-ray Absorption Fine Structure Spectra Acquired for β-$Pb_x$/β'-$Cu_yV_2O_5$.*** **(A)** Pb $L_3$-edge XANES spectra of β-$Pb_x$/β'-$Cu_yV_2O_5$. The Pb L3 edge XANES spectrum of β-$Pb_x$/β'-$Cu_yV_2O_5$ does not have a pre-edge feature, which is an indication that the Pb 6s orbitals are fully occupied, as expected for divalent lead. **(B)** $k^3$-weighted Fourier transforms of the Pb $L_3$-edge EXAFS spectra. Analysis of the R-space Pb $L_3$-edge EXAFS spectra indicates that the first and second coordination shells at approximately 1.90 and 3.40Å correspond to the contributions from Pb−O and Pb−Pb scattering paths respectively. The fitted R-space EXAFS spectra are shown in **Figure S23**; fitting parameters related to the major scattering paths are provided in **Table S42**. The inferred Pb−O distances for β-$Pb_x$/β'-$Cu_yV_2O_5$ (**Figure S23** and **Table S42**) are consistent with values reported for other divalent lead oxides with stereochemically active lone pairs such as PbO,[3,4] and $PbGa_2O_4$.

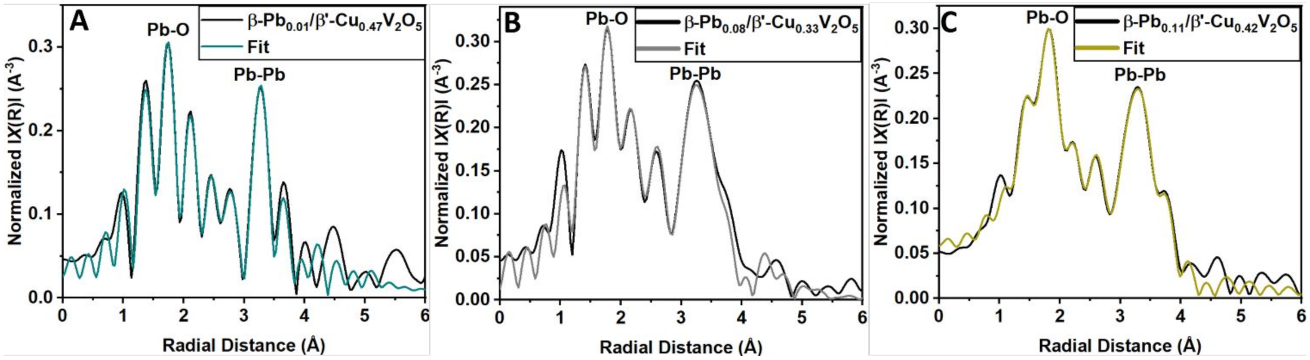


***Figure S23. Fitting results of the Pb $L_3$ EXAFS spectra of β-$Pb_x$/β'-$Cu_yV_2O_5$.*** The fitted R-space Pb $L_3$-edge EXAFS spectra for **(A)** β-$Pb_{0.01}$/β'-$Cu_{0.47}V_2O_5$ **( B)** β-$Pb_{0.08}$/β'-$Cu_{0.33}V_2O_5$, and **(C)** β-$Pb_{0.11}$/β'-$Cu_{0.41}V_2O_5$. The fitting of the features at 1.90 and 3.4 yields Pb−O and Pb−Pb bond lengths of 2.31 and 3.64Å for β-$Pb_{0.01}$/β'-$Cu_{0.47}V_2O_5$; 2.21 and 3.65Å for β-$Pb_{0.08}$/β'-$Cu_{0.33}V_2O_5$; and 2.26 and 3.67Å for β-$Pb_{0.11}$/β'-$Cu_{0.41}V_2O_5$. The derived Pb−O and Pb-

Pb distances of β-$Pb_x$/β'-$Cu_yV_2O_5$ (Table S42) are consistent with $Pb^{2+}$–O and Pb—Pb distances reported for PbO (2.285, 3.62 Å),[3,4] $PbSiO_3$ (2.27 Å),[4,5] and $PbGa_2O_4$ (2.31 Å).[4]

**Table S42. Tabulated Pb $L_3$-edge EXAFS fitting parameters of β-$Pb_x$/β'-$Cu_yV_2O_5$.** Atomic positions obtained from high-resolution single-crystal X-ray diffraction (Tables S13-S38) were used as initial models to fit the EXAFS data.

| Sample | Path | N | R(Å) | $\sigma^2$(Å)$^2$ | $S_0^2$ | $E_0$ (eV) | R-factor (%) |
|---|---|---|---|---|---|---|---|
| **β-$Pb_{0.01}$/β'-$Cu_{0.47}V_2O_5$** | Pb—Cu | 2 | 2.181 | 0.004 | 1.0 | 9.474 ± 1.013 | 0.3 |
| | Pb—O | 1 | 2.308 | | | | |
| | Pb—Pb | 2 | 3.639 | | | | |
| **β-$Pb_{0.08}$/β'-$Cu_{0.33}V_2O_5$** | Pb—Cu | 2 | 2.120 | 0.003 | 1.0 | 5.150 ± 3.155 | 0.2 |
| | Pb—O | 1 | 2.213 | | | | |
| | Pb—Pb | 2 | 3.649 | | | | |
| **β-$Pb_{0.11}$/β'-$Cu_{0.41}V_2O_5$** | Pb—Cu | 2 | 2.083 | 0.006 | 1.0 | 6.744 ± 0.209 | 0.9 |
| | Pb—O | 1 | 2.261 | | | | |
| | Pb—Pb | 2 | 3.673 | | | | |

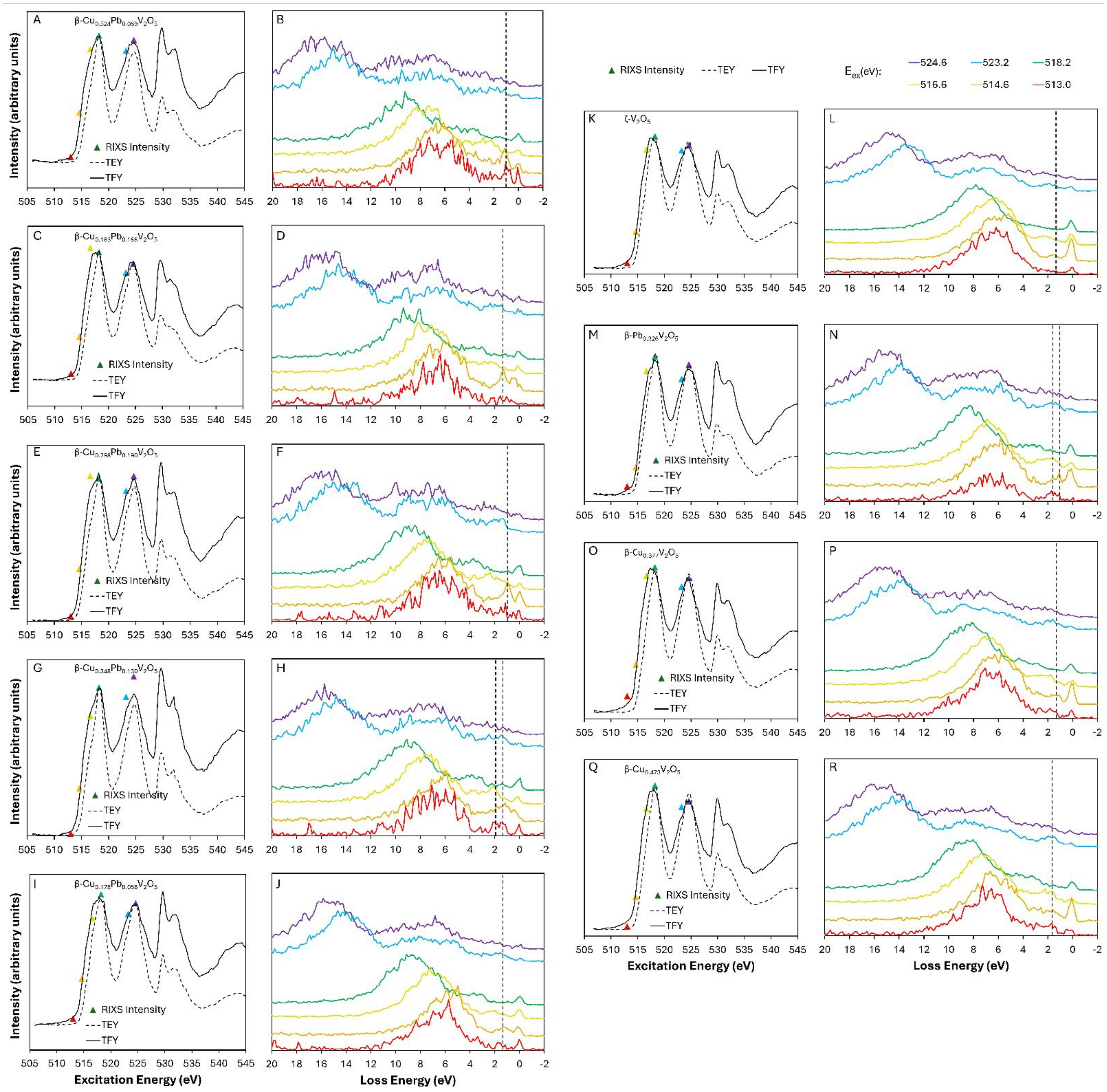


***Figure S24**:* V L2,3-edge XAS spectra and corresponding RIXS slices, respectively, for **(A,B)** β-$Pb_{0.069}$/β'-$Cu_{0.524}V_2O_5$, **(C,D)** β-$Pb_{0.188}$/β'-$Cu_{0.183}V_2O_5$, **(E,F)** β-$Pb_{0.190}$/β'-$Cu_{0.298}V_2O_5$, **(G,H)** β-$Pb_{0.130}$/β'-$Cu_{0.348}V_2O_5$, **(I,J)** β-$Pb_{0.056}$/β'-$Cu_{0.178}V_2O_5$, **(K,L)** ζ-$V_2O_5$, **(M,N)** β-$Pb_{0.326}V_2O_5$, **(O,P)** β'-$Cu_{0.377}V_2O_5$, **(Q,R)** β'-$Cu_{0.473}V_2O_5$. TEY in XANES plots corresponds to total electron yield, and TFY to total fluorescence yield. RIXS excitation energies and integrated emission intensities indicated by triangles. Although the RIXS spectra are dominated by a large fluorescence feature (shifting from ca. 6 eV to 10 eV loss energy with increasing excitation energy), d-d excitation features are visible in the 0-2 eV loss energy range, indicated by vertical dashed lines.

**Table S43.** Comparison of bonding–antibonding (B–AB) energy separations for β-$Pb_x$/β'-$Cu_yV_2O_5$ and selected compounds with known stereochemical expression of 5/6$s^2$ lone pair states, illustrating the relative degree of lone-pair stereoactivity.

| Compound | Δ$E$ | References |
|---|---|---|
| δ-$Pb_{0.5}V_2O_5$ | 7.6 | [6,7] |
| δ-$Tl_{0.5}V_2O_5$ | 5.4 | [6] |
| β-$Pb_{0.33}V_2O_5$ | 7.6 | [6,8] |
| β-$Sn_{0.33}V_2O_5$ | 9.3 | [6] |
| $PbVO_3Cl$ | 8.5 | [9] |
| β-$Pb_x$/β'-$Cu_yV_2O_5$ | 8.1 | This work |

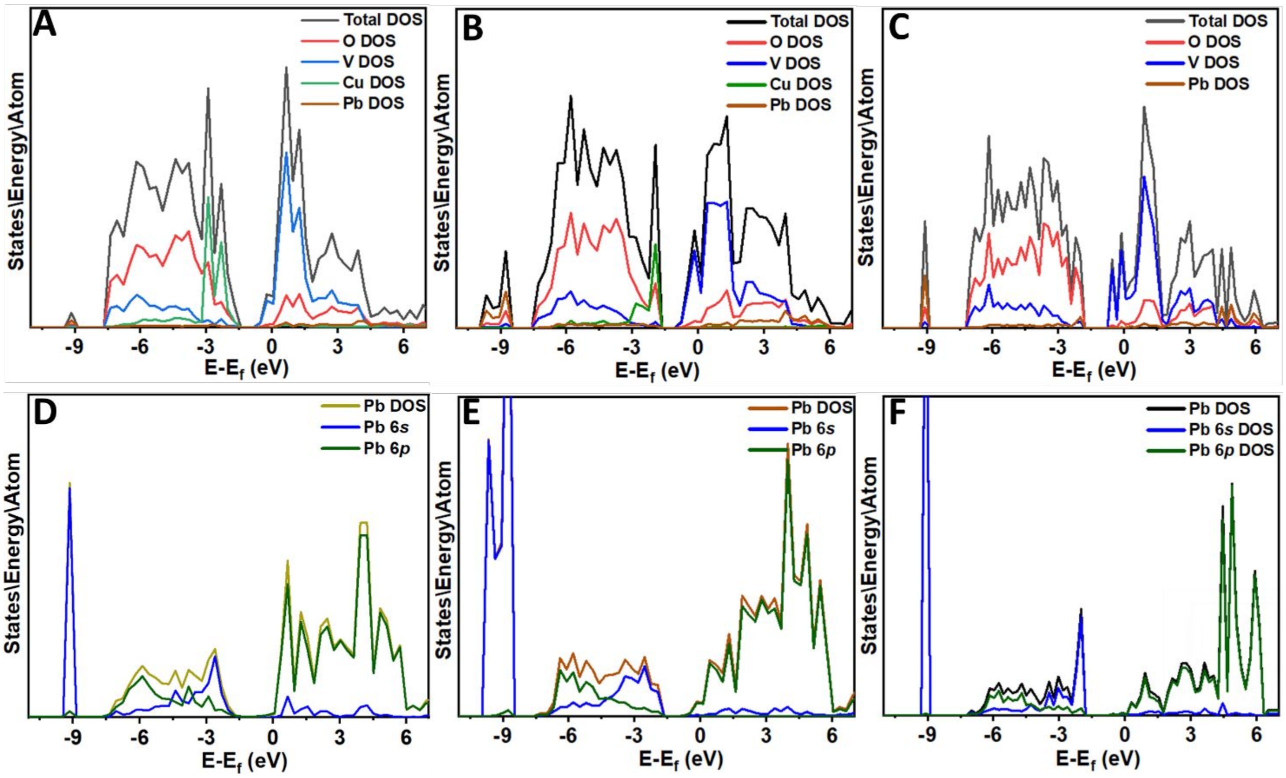


***Figure S25. Density of States as calculated from ground state DFT+U calculations.*** Total and atom-projected density of states for **(A)** β-$Pb_{0.17}$/β'-$Cu_{0.33}V_2O_5$, **(B)** β-$Pb_{0.33}$/β'-$Cu_{0.17}V_2O_5$ , and **(C)** β-$Pb_{0.33}V_2O_5$. Orbital-projected density of states for Pb 6s and 6p in **(D)** β-$Pb_{0.17}$/β'-$Cu_{0.33}V_2O_5$, **(E)** β-$Pb_{0.33}$/β'-$Cu_{0.17}V_2O_5$, and **(F)** β-$Pb_{0.33}V_2O_5$.

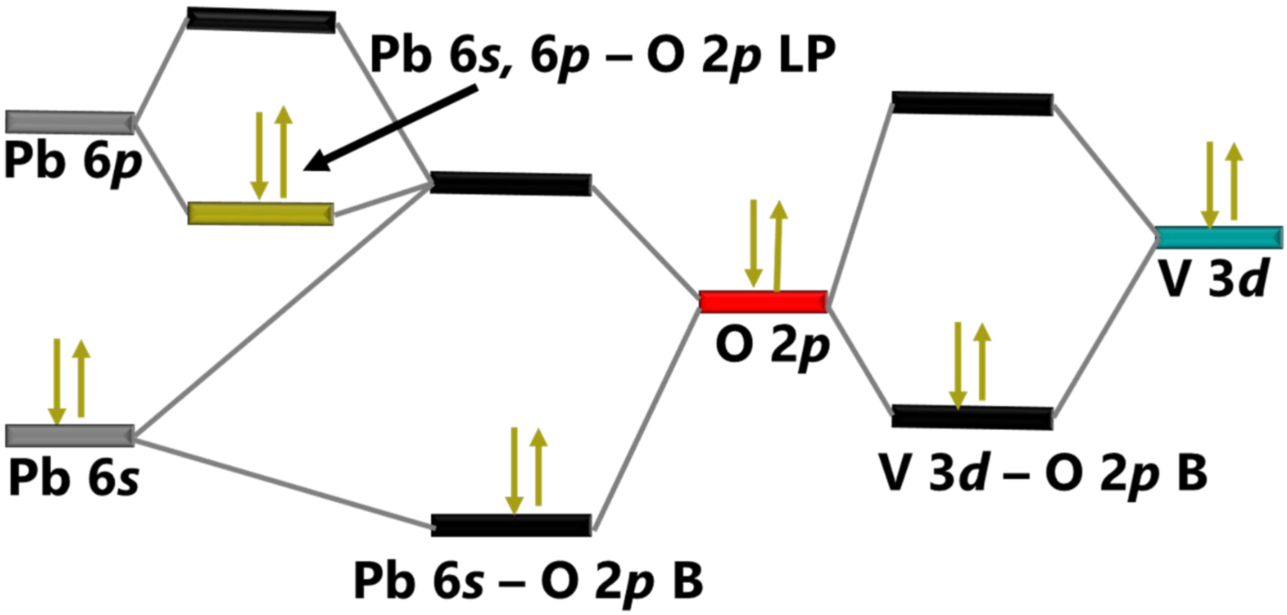


***Figure S26. A molecular orbital perspective of electronic structure modulation of β-$Pb_x$/β'-$Cu_yV_2O_5$.*** Molecular orbital diagram illustrating the hybridization between different atomic orbitals in β-$Pb_x$/β'-$Cu_yV_2O_5$. Cu hybridization is excluded for clarity. The highest occupied molecular orbital (HOMO) states highlighted in dark gold correspond to Pb 6s—O 2p AB state (with weak V 3d character; V-O-Pb hybridized "in-gap" state) positioned above the non-bonding O 2p states and ~0.9 eV below the Fermi level. Furthermore, the V 3d states hybridize with O 2p states giving rise to V 3*d* − O 2*p* B and AB states. The unoccupied V 3d − O 2p AB states in the conduction band are the lowest unoccupied molecular orbital (LUMO) states in the electronic structure of β-$Pb_x$/β'-$Cu_yV_2O_5$.

*Acknowledgement*
*This research used resources of the National Synchrotron Light Source II, a U.S. Department of Energy (DOE) Office of Science User Facility operated for the DOE Office of Science by Brookhaven National Laboratory under contract DE-SC0012704.*

*Disclaimer*
*Commercial equipment, instruments, or materials are identified in this paper to describe the experimental procedure and conditions adequately. Such an identification is not intended to imply recommendation or endorsement by the National Institute of Standards and Technology, nor is it intended to imply that the materials or equipment identified are necessarily the best available for the purpose.*

## References

(1) Ponis, J.; Hariyani, S.; Agbeworvi, G.; Chakraborty, S.; Balcorta, V.; Pérez-Vázquez, J.; Rogers, B. L.; Chiang, Y.-H.; Jessel, A.; Brown, T. D.; Williams, R. S.; Pharr, M.; Qian, X.; Banerjee, S. Single Crystals of Vanadium Oxides as a Lens for Understanding Structural and Electronic Phase Transformations, Ion Transport, Chemo-Mechanical Coupling, and Electrothermal Neuronal Emulation. *Chem. Rev.* **2025**, *125* (21), 10657–10764. https://doi.org/10.1021/acs.chemrev.5c00413.

(2) Parija, A.; Handy, J. V.; Andrews, J. L.; Wu, J.; Wangoh, L.; Singh, S.; Jozwiak, C.; Bostwick, A.; Rotenberg, E.; Yang, W.; Fakra, S. C.; Al-Hashimi, M.; Sambandamurthy, G.; Piper, L. F. J.; Williams, R. S.; Prendergast, D.; Banerjee, S. Metal-Insulator Transitions in Β′-CuxV2O5 Mediated by Polaron Oscillation

and Cation Shuttling. *Matter* **2020**, *2* (5), 1166–1186. https://doi.org/10.1016/J.MATT.2020.01.027.
(3) Yu, Y. I. X.; Tyliszczak, T.; Hitchcock, A. P. Pb L3 EXAFS and Near-Edge Studies of Pb Metal and Lead Oxides. **1990**, *51* (3).
(4) Gyu, Y.; Hon, K.; Chernov, V. A.; Heo, J. Pb LIII-Edge EXAFS and XANES Analyses on the Structural Environment of Lead in PbO ± Ga 2 O 3 Glasses. **1999**, *246*, 128–135.
(5) Witkowska, A.; Rybicki, J.; Trzebiatowski, K.; Di, A.; Minicucci, M. In ¯ Uence of Hydrogen Reduction on the Structure of PbSiO 3 Glass : An EXAFS Study. **2000**, *276*, 22–29.
(6) Razek, S. A.; Popeil, M. R.; Wangoh, L.; Rana, J.; Suwandaratne, N.; Andrews, J. L.; Fwatson, D.; Banerjee, S.; Piper, L. F. J. Designing Catalysts for Water Splitting Based on Electronic Structure Considerations. *Electron. Struct.* **2020**, *2* (2), 023001. https://doi.org/10.1088/2516-1075/ab7d86.
(7) Agbeworvi, G.; Zaheer, W.; Handy, J. V.; Andrews, J. L.; Perez-Beltran, S.; Jaye, C.; Weiland, C.; Fischer, D. A.; Balbuena, P. B.; Banerjee, S. Toggling Stereochemical Activity through Interstitial Positioning of Cations between 2D V2O5 Double Layers. *Chem. Mater.* **2023**, *35* (17), 7175–7188. https://doi.org/10.1021/acs.chemmater.3c01463.
(8) Wangoh, L.; Marley, P. M.; Quackenbush, N. F.; Sallis, S.; Fischer, D. A.; Woicik, J. C.; Banerjee, S.; Piper, L. F. J. Electron Lone Pair Distortion Facilitated Metal-Insulator Transition in β-Pb0.33V2O5 Nanowires. *Appl. Phys. Lett.* **2014**, *104* (18), 7–11. https://doi.org/10.1063/1.4875747.
(9) Dang, U.; Zaheer, W.; Zhou, W.; Kandel, A.; Orr, M.; Schwenz, R. W.; Laurita, G.; Banerjee, S.; MacAluso, R. T. Lattice Anharmonicity of Stereochemically Active Lone Pairs Controls Thermochromic Band Gap Reduction of PbVO3Cl. *Chem. Mater.* **2020**, *32* (17), 7404–7412. https://doi.org/10.1021/acs.chemmater.0c02342.
